# Hemp and buckwheat are valuable sources of dietary amino acids, beneficially modulating gastrointestinal hormones and promoting satiety in healthy volunteers

**DOI:** 10.1007/s00394-021-02711-z

**Published:** 2021-10-30

**Authors:** Madalina Neacsu, Nicholas J. Vaughan, Salvatore Multari, Elisabeth Haljas, Lorraine Scobbie, Gary J. Duncan, Louise Cantlay, Claire Fyfe, Susan Anderson, Graham Horgan, Alexandra M. Johnstone, Wendy R. Russell

**Affiliations:** 1 The Rowett Institute, University of Aberdeen, Aberdeen, AB25 2ZD Scotland, UK; 2 Biomathematics and Statistics Scotland, Aberdeen, AB25 2ZD Scotland, UK

**Keywords:** Plant foods, Plant protein, Gut hormones, Hunger, Phytochemicals bioavailability, Amino acids

## Abstract

**Purpose:**

This study evaluated the postprandial effects following consumption of buckwheat, fava bean, pea, hemp and lupin compared to meat (beef); focussing on biomarkers of satiety, gut hormones, aminoacids and plant metabolites bioavailability and metabolism.

**Methods:**

Ten subjects (*n* = 3 men; *n* = 7 women; 42 ± 11.8 years of age; BMI 26 ± 5.8 kg/m^2^) participated in six 1-day independent acute interventions, each meal containing 30 g of protein from buckwheat, fava bean, pea, hemp, lupin and meat (beef). Blood samples were collected during 24-h and VAS questionnaires over 5-h.

**Results:**

Volunteers consumed significantly higher amounts of most amino acids from the meat meal, and with few exceptions, postprandial composition of plasma amino acids was not significantly different after consuming the plant-based meals. Buckwheat meal was the most satious (300 min hunger scores, *p* < 0.05).Significant increase in GLP-1 plasma (AUC, iAUC *p* = 0.01) found after hemp compared with the other plant-based meals. Decreased plasma ghrelin concentrations (iAUC *p* < 0.05) found on plant (hemp) vs. meat meal. Several plasma metabolites after hemp meal consumption were associated with hormone trends (partial least squares-discriminant analysis (PLS-DA): 4-hydroxyphenylpyruvic acid, indole 3-pyruvic acid, 5-hydoxytryptophan, genistein and biochanin A with GLP-1, PYY and insulin; 3-hydroxymandelic acid and luteolidin with GLP-1 and ghrelin and 4-hydroxymandelic acid, benzoic acid and secoisolariciresinol with insulin and ghrelin. Plasma branched-chain amino acids (BCAAs), (iAUC, *p* < 0.001); and phenylalanine and tyrosine (iAUC, *p* < 0.05) were lower after buckwheat comparison with meat meal.

**Conclusion:**

Plants are valuable sources of amino acids which are promoting satiety. The impact of hemp and buckwheat on GLP-1 and, respectively, BCAAs should be explored further as could be relevant for aid and prevention of chronic diseases such as type 2 diabetes.

Study registered with clinicaltrial.gov on 12th July 2013, study ID number: NCT01898351.

**Supplementary Information:**

The online version contains supplementary material available at 10.1007/s00394-021-02711-z.

## Introduction

With an increase in the world population comes a requirement to produce more food, along with observed higher rates in consumption of animal-based protein. This demand is also driven by socio-economic factors such as rising incomes and increased urbanisation [1]. Maintaining these current consumption trends will further deplete natural resources and livestock production is known to be a major contributor to greenhouse gas (GHG) emissions [2]. At the same time, infectious diseases are being increasingly replaced by diet-related non-communicable diseases such as type 2 diabetes mellitus (T2D), cardiovascular disease (CVD) and cancer as the major causes of death. The global population needs affordable, sustainable, and healthy dietary protein. Thus, access to plant-based protein sources could be a viable option to feed the rising population providing they can meet amino acid requirements, are satiating and acceptable.

Certain amino acids are considered to be detrimental and high circulating concentrations of branched-chain (BCAAs) and aromatic amino acids (AAAs) are associated with several characteristics of T2D, such as increased glycaemia and insulin resistance [3–6] BCAAs (isoleucine, leucine, valine) and AAAs (tyrosine and phenylalanine) were shown to have highly significant associations with risk of future diabetes development [4]. Microbial-derived metabolites of AAAs found in plasma such as indole-3-propionic acid (IPA) was also associated with a lower likelihood of developing T2D [7] in people with impaired glucose tolerance. Furthermore, the indoles were also recognised as possessing anti-oxidative and anti-inflammatory properties [8–10], as well as being potent stimulators of the gut hormone GLP-1 [11]. Satiety hormones consisting of ghrelin, peptide YY (PYY) and glucagon-like peptide-1 (GLP-1), regulate calorie intake through their appetite-stimulating (orexigenic) or appetite-inhibiting (anorexigenic) effects [12]. Ghrelin, is secreted by endocrine cells in stomach, increases during the pre-prandial state leading to hunger and desire for food intake [13]. PYY levels are low in the fasting state and is released from the gut into the circulation in a nutrient-dependent manner, rapidly increasing in response to food intake [14]. The PYY levels are influenced by caloric load, macronutrient composition and food consistency [14–16]. GLP-1 is a potent incretin hormone produced in the L cells of the distal ileum and colon and acts as a neurotransmitter (satiety and loss appetite) [17]. It delays gastric emptying and inhibits pentagastrin and acid secretion stimulated by food ingestion [17]. Also has cardiovascular benefits on blood pressure, the vascular endothelium, atherosclerosis progression and inflammation, myocardial ischaemia, heart failure as reviewed by Olmo Garcia et al. [17]. Nutrients, including glucose, fatty acids, and dietary fiber [18], are all known to upregulate the transcription of the gene encoding GLP-1, and they can stimulate the release of this hormone, the levels of GLP-1 rise rapidly upon food ingestion. Since gut-derived hormones therefore influence a range of physiological processes, including metabolic pathways performing regulatory roles in glucose homeostasis, centrally mediated appetite control and adiposity they could be targets for novel obesity and diabetes therapies. It is important to understand the effect of plant-based foods on the modulation of these hormones as diets rich in high-protein crops are inversely associated with the risk of metabolic syndrome [19], with lower incidence of T2D [20], and with the improvement of several biomarkers of CVD [21].

The observed protective effect of consuming plant-based foods on chronic diseases is often attributed to the presence of bioactive components [22–24]. Abundant plant-derived bioactive compounds include phytosterols, phytoestrogens, flavonoids, carotenoids and other phytophenols. These, along with plant macronutrients and their circulatory metabolites are the focus of a vast amount of research in animal models and human studies and several literature reviews summarise the effects of these on CVD and T2D [25–28]. These findings underscore the potential key role of amino acid metabolism, as well as non-nutrient phytochemicals in the early pathogenesis of T2D and CVD and suggest that these profiles could aid in risk assessment [4].

The objectives of the current work was to assess the postprandial events (biomarkers of satiety and gut hormone response) as well as, amino acid and bioactive phytochemicals bioavailability following acute consumption of meals prepared from buckwheat, fava bean, green pea, hemp, lupin, compared to meat (beef). This study provides important nutritional information that support plants as viable sources of dietary protein and other nutrients.

## Subjects and methods

### Subjects

For this study, ten healthy volunteers (males and females), 18–65 years old, with BMI 18–35 kg m^−2^, non-vegetarian, non-smokers, with no known allergies and taking no prescribed medication were recruited. The study started in March 2013. All volunteers completed a medical questionnaire at the Human Nutrition Unit (HNU) assisted by the general practitioner (GP). Volunteers’ height was measured to the nearest 0.1 cm with the use of a stadiometer (Holtain Ltd, Crymych, Dyfed, UK) and the weight was measured to the nearest 100 g on a digital scale (DIGI DS-410; CMS Weighing Equipment, London, UK). The screening included a Skin Prick Test (SPT), and in vitro specific Immunoglobulin E (IgE) test (for all the flours used) performed at Aberdeen Royal Infirmary Hospital Foresterhill. The study was approved by the North of Scotland Research Ethics Service, with REC reference number: 13/NS/0006. After signing a consent form, the volunteers participated in seven visits to the HNU at The Rowett Institute (RI), Aberdeen, UK, from which, one was the screening visit and six were the 1-day dietary interventions (with a washout of minimum 2 weeks between interventions). The intervention meals were served in the following order: fava bean, lupin, meat, green pea, buckwheat and hemp.

### Study intervention visits

**Fig. 1:**
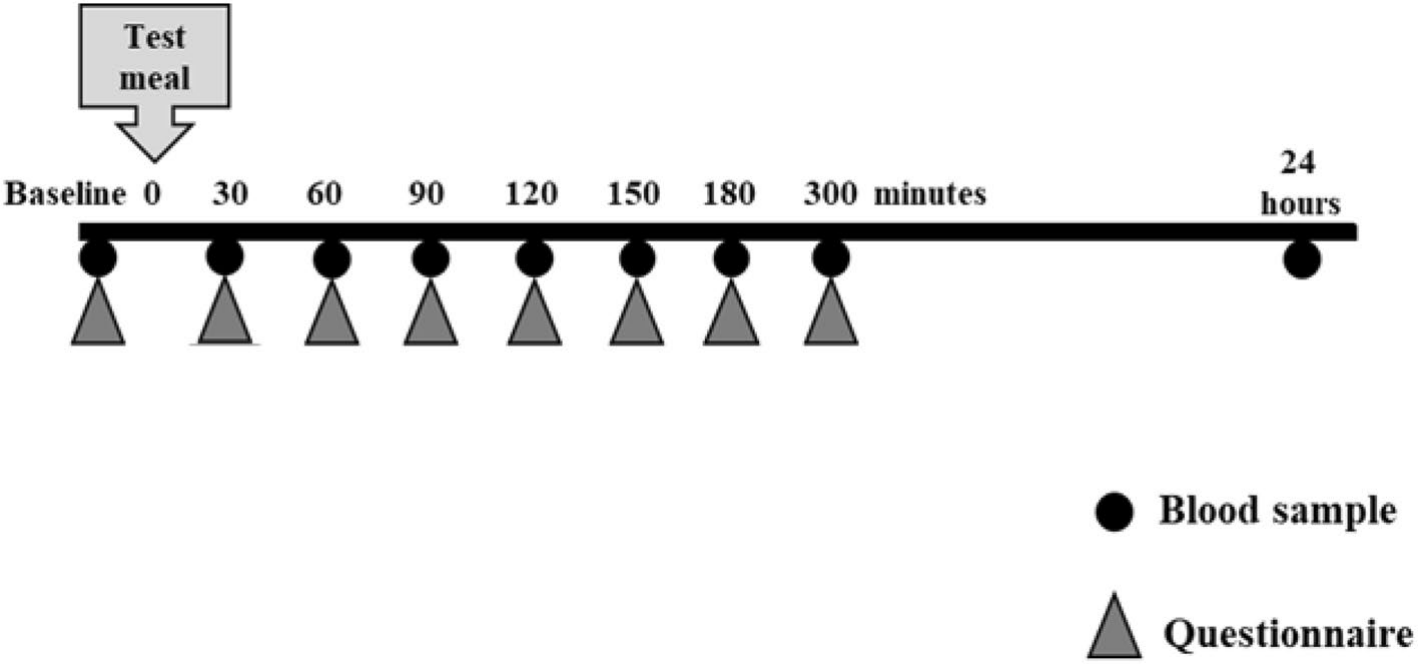
Intervention day sampling diagram for bloods and questionnaires

Prior to each interventions the volunteers recorded their habitual diet in a 7-day weighed food diary. The volunteers were instructed to come after an overnight fast, and instructed to consume the test meal within 30 min. Five hours later, an ad libitum lunch meal was offered. Besides visual analogue scales (VAS) questionnaires, blood samples were taken throughout a 24-h period. Blood samples were collected by using a cannula into lithium heparin tubes at baseline (0 min) and after 30, 60, 90, 120, 150, 180, 300 min and 24 h from the commencement of the intervention meal (Fig. 1). The plasma was separated (3300×*g*; 15 min; 4 °C) within 5 min of collection and stored at − 70 °C until analysis. Six different 10-cm VAS were used to assess satiety before the test meal and at 30, 60, 90, 120, 150, 180 and 300 min after the test meal, as well as after the consumption of the ad libitum lunch. The VAS questionnaires included the following six common questions [29] as previously described [21].

### Test meals

Each test meal (bread roll weight) was designed to deliver 30 g protein from the five different plant-based sources (in flour form of green pea, lupin, hemp, buckwheat or fava beans) and, respectively, meat and used same amount of wheat flour (125 g) for each breadroll including the meat meal. Each test meal was served with 30 g butter. Commercially available flours were obtained as follows: strong wheat flour was purchased from Tesco Stores Ltd. (Cheshunt, UK); buckwheat flour was purchased from Arrowhead Millls, Inc. (Melville, USA); hemp flour was purchased from Yorkshire Hemp Ltd. (Driffield UK); fava bean flour was purchased from The Barry Farm (Wapakoneta USA); green pea flour was purchased from Bob’s Red Mill Natural Foods (Milkwaukie, USA); lupin flour was purchased from Terrena Lup’ Ingredients (Martigne Ferchaud, France) and the lean beef frying steak Tesco Stores Ltd. The number of rolls served for each intervention meal varied: two rolls (lupin, hemp), and three rolls (fava bean, green pea, buckwheat). The meat meal consisted in a fried meat (containing 30 g protein) and a wheat bread roll served with 30 g butter. The protein content was measured as crude protein content, %N and multiplied with a 6.25 factor. Same quantity and composition of ad libitum lunch (pasta Bolognese) containing 15% protein, 30% fat and 55% carbohydrate was provided 5 h after the test meal, and instructed to “eat until comfortably full.” The test meals and ad libitum ingredients are presented in Tables S 1 A and B from Online Resource ESM_1.

### Composition of test meals

Macronutrients (protein, including amino acid composition (excluding Tryptophan); fibre measured as soluble and insoluble non-starch polysaccharide and fat), micronutrient minerals and phytochemical metabolites were measured by standard published protocols as described in our previous publication [30].

### Compliance and metabolic profile

The volunteers attended the HNU on 6 intervention days, where they consumed the individual test meals. The blood sampling was performed as described in Fig. 1. Blood samples were used to measure the plasma amino acids by isotope dilution (different methodology from the food amino acid determination), with a gravimetric approach (to give μM) described previously [31], for homocysteine (HCys) [32] and arginine [33]. Total amino acid concentrations were used as a biomarker for protein metabolism. All the plasma metabolites lipid profile, glucose, insulin, other phytochemicals, were measured as described before [34–36]. Based on preliminary VAS scores, the total PYY, total ghrelin, and active GLP-1 were analysed from the plasma samples collected (enzyme inhibitors were added to the plasma) from fava bean, meat, buckwheat and hemp meals using ELISA (Millipore) kits as previously described [21].

### Statistical analysis

The sample size of ten volunteers was predicted to detect a treatment effect 1.4 times the magnitude of the within volunteer spread, with 80% power at the 5% significance level. For glucose and insulin, the within volunteer spread was approximately 10% (based on previous studies), which gave the study power to detect a treatment shift of ca. 14%. Data were analysed by analysis of variance (ANOVA) of area under the curve (AUC) and iAUC of data collected over multiple time points following consumption of the test meal, as well as the data at each time point individually. The ANOVA included terms for volunteer, baseline value and diet. A version of the ANOVA in which the diet term was split into a contrast between the meat diet and the plant diet, and the variation among the plant diets was also examined. Differences among the diets were assessed by post hoc tests with Tukey's adjustment for multiple comparisons. Data were checked for the assumptions of normality and constant variance, and were log transformed before analysis where appropriate. All analyses were carried out using Genstat 17 (Lawes Agricultural Trust, VSN international Ltd, Hemel Hempstead, UK). The plant metabolites (non-nutrients) from test meals, plasma phenylpropanoid pathway and products of protein and carbohydrate metabolism data was analysed by principal component analysis (PCA), unit variance (UV)-scaled using SIMCA 14.1 (Umetrics, Cambridge). The correlation between plasma metabolites and gastrointestinal hormones was analysed by partial least squares-discriminant analysis (PLS-DA) SIMCA 14.1 (Umetrics, Cambridge). The effect of test meals over time and between test meals (plasma) as well as the differences between the amounts of amino acids eaten for each interventions were assessed by two‐sided post hoc *t* tests. A version of the analysis which accounted for the varying protein intake was also conducted, by including a term for amount consumed and its interaction with the protein type.

## Results

### Subjects baseline characteristics

Ten subjects (*n* = 3 men; *n* = 7 women) with a mean age of 42 ± 11.8 (years) and BMI of 26 ± 5.8 (kg m^−2^) (reflecting the average BMI of the Scottish adult population) were recruited for this study.

### Composition of the study test meals

#### Macronutrients

The protein, fat, soluble and insoluble NSP content of the meals are presented in Fig S1A–D (ESM_1).

#### Amino acids

Amino acid composition of the test meals is presented in Table 1 including ANOVA Fisher’s test values for each amino acid for each test meal. The concentrations of histidine (His), serine (Ser), arginine (Arg), glycine (Gly), aspartic acid (Asp), glutamic acid (Glu), threonine (Thr), alanine (Ala), proline (Pro), lysine (Lys), tyrosine (Tyr), valine (Val), isoleucine (ILeu), leucine (Leu), phenylalanine (Phe), methionine (Met) and cysteine (Cys) are presented in mmoles/ portion test meal. Overall, the quantity of individual amino acids was significantly different across the test meals: His, Ser, Arg, Asp, Thr, Ala, Pro, Lys, Tyr, Val, Ile, Leu, Phe, Met (*p* < 0.001), Gly, Cys (*p* = 0.002) and Glu (*p* = 0.005). The meat meal had significantly higher levels of Lys, Val, Ile and Met and was richest in Ala, Thr and His compared with the other plant-based meals The buckwheat meal was richest in Cys; the fava bean meal significantly higher in Pro and richest in Tyr; the green pea meal significantly higher in Gly, Ser, Asp, and richest in Phe and Glu and the hemp meal significantly higher in Arg.

**Table 1:** The amino acid concentrations of the test meals in mmoles/portion test meal ± SD (Fisher's test) for histidine (His), serine (Ser), arginine (Arg), glycine (Gly), aspartic acid (Asp), glutamic acid (Glu), threonine (Thr), alanine (Ala), proline (Pro), lysine (Lys), tyrosine (Tyr), valine (Val), isoleucine (ILeu), leucine (Leu), phenylalanine (Phe), methionine (Met) and cysteine (Cys)

Amino acid	mmoles/portion test meal ± SD (Fisher's test)					
	Buckwheat	Fava bean	Green pea	Hemp	Lupin	Meat
Ala	15.19 ± 0.55(a)	20.62 ± 1.03(cd)	19.17 ± 0.67(bc)	17.43 ± 0.45(b)	14.7 ± 0.11(a)	22.38 ± 0.92(d)
Gly	24.26 ± 0.87(ab)	24.98 ± 1.36(b)	25.72 ± 0.71(b)	24.06 ± 0.61(ab)	21.77 ± 0.41(a)	25.65 ± 1.28(b)
Val	14.67 ± 0.57(ab)	14.55 ± 0.59(ab)	15.76 ± 0.4(b)	14.44 ± 0.11(a)	14.41 ± 0.36(a)	17.59 ± 0.61(c)
Leu	18.01 ± 0.72(a)	24.91 ± 1.19(c)	24.29 ± 0.45(c)	20.88 ± 0.29(b)	22 ± 0.41(b)	24.92 ± 0.83(c)
Ile	9.72 ± 0.38(a)	9.87 ± 0.34(a)	11.37 ± 0.24(b)	9.74 ± 0.09(a)	11.88 ± 0.42(b)	13.57 ± 0.5(c)
Pro	21.8 ± 0.91(a)	28.61 ± 1.48(d)	26.29 ± 0.54(c)	26.26 ± 0.41(c)	23.75 ± 0.18(ab)	24.99 ± 0.66(bc)
Met	4.83 ± 0.37(c)	4.19 ± 0.32(bc)	3.6 ± 0.09(ab)	5.94 ± 0.22(d)	3.07 ± 0.18(a)	6.75 ± 0.24(e)
Ser	16.89 ± 0.41(a)	22.72 ± 1.12(d)	23.12 ± 0.66(d)	20.98 ± 0.33(c)	19.56 ± 0.29(bc)	17.98 ± 0.52(ab)
Thr	10.2 ± 0.33(a)	12.9 ± 0.64(c)	13.25 ± 0.25(cd)	11.42 ± 0.14(b)	11.59 ± 0.24(b)	14.09 ± 0.51(d)
Phe	10.05 ± 0.55(a)	11.76 ± 0.87(cd)	12.97 ± 0.19(d)	11.44 ± 0.38(bc)	10.19 ± 0.29(ab)	11.27 ± 0.33(abc)
Asp	21.06 ± 0.78(a)	28.4 ± 1.48(cd)	31.22 ± 1.74(d)	25.86 ± 1.32(bc)	24.18 ± 0.65(ab)	25.87 ± 1.39(bc)
Cys	9.63 ± 0.67(b)	7.3 ± 0.48(a)	7.11 ± 0.55(a)	7.69 ± 0.4(a)	8.11 ± 0.7(ab)	8.28 ± 0.62(ab)
Glu	57.82 ± 1.86(a)	65.72 ± 3.49(b)	67.78 ± 3.1(b)	61.51 ± 2.75(ab)	63.56 ± 0.79(ab)	62.88 ± 2.16(ab)
Lys	9.94 ± 0.31(bc)	11.18 ± 0.84(c)	13.79 ± 0.47(d)	6.85 ± 0.26(a)	8.9 ± 0.09(b)	17.23 ± 0.84(e)
Arg	14.59 ± 0.6(a)	17.01 ± 0.96(a)	17.82 ± 0.29(b)	19.63 ± 0.54(c)	17.42 ± 0.65(b)	13.49 ± 0.47(a)
His	4.99 ± 0.2(a)	6.47 ± 0.37(cd)	6.26 ± 0.14(cd)	5.85 ± 0.2(bc)	5.25 ± 0.16(ab)	6.78 ± 0.36(d)
Tyr	5.55 ± 0.32(a)	8.32 ± 0.64(c)	7.86 ± 0.07(bc)	7.02 ± 0.28(b)	8.17 ± 0.6(c)	8.12 ± 0.34(bc)

Where a, b, c, d represents ANOVA Fisher’s test values for each amino acid

#### Micronutrients

The micronutrients content of the meals are presented in Fig S 2A–C.

#### Non-nutrient plant metabolites

**Fig. 2:**
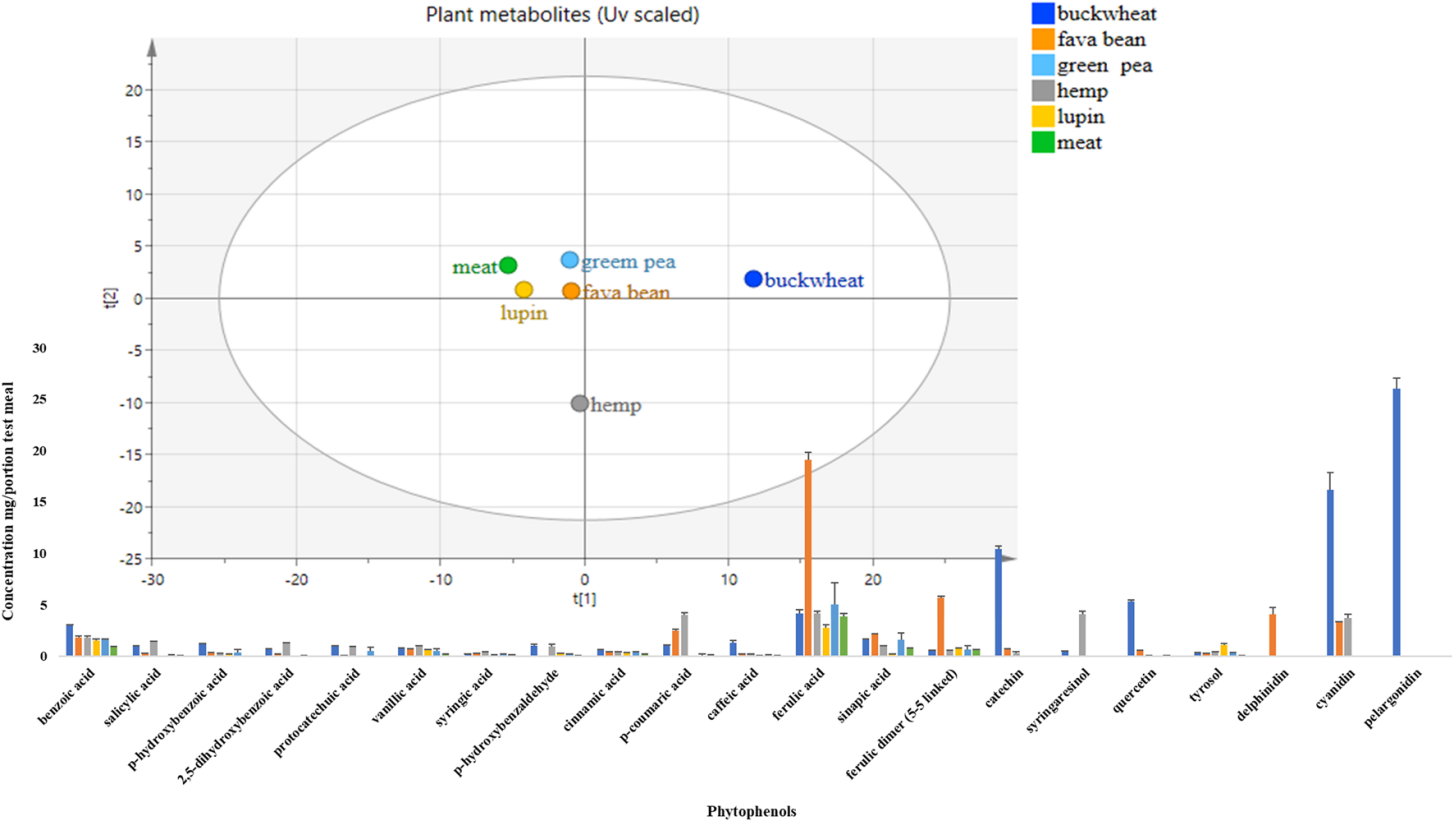
Principal component analysis (PCA), (unit variance (UV) scaled) of plant non-nutrients (measured in triplicate) from test meals was carried out and the first two component scores were plotted. Test meal plant metabolites composition (10 most abundant phytophenols) in mg/test meal ± SD, *n* = 3

The ten most abundant phytophenols found in each test meal are presented in Fig. 2. Buckwheat meal had the highest amount of total plant metabolites measured (164.6 mg/portion), followed by hemp and fava bean meals (82.6 and 81.1 mg/portion, respectively) and PCA (Fig. 2) of all metabolites showed clear discriminations of buckwheat and hemp from the rest of the meals. The clustering of meat with the green pea, lupin and fava bean meals in the top left quadrant is likely to be due to the similarity of the profiles with the wheat portion of the meals; specifically the presence of ferulic, benzoic and sinapic acid. However, ferulic acid was the most abundant phytophenol in the fava bean meal and was significantly higher (*p* < 0.001) in comparison with the meat meal. The clear segregation of buckwheat and hemp meal is likely to be due to the presence of anthocyanins (pelargonidin) being present only in the buckwheat meal and syringaresinol being significantly higher in the hemp meal (*p* < 0.001). The complete profile of plant metabolites is presented in Table S2 A-D from supplementary data ESM_1.

### Plasma metabolites

For the meat meal, a higher average intake (93.5% of the meal served) was recorded. This was followed by lupin (80.9%) and hemp (69.7%). The least quantity consumed of all the intervention meals was fava bean, with an average intake of 65.0% of the meal served; green pea and buckwheat meals had similar (*p* > 0.05) average intakes of 67.1 and 69.7%, respectively.

**Fig. 3:**
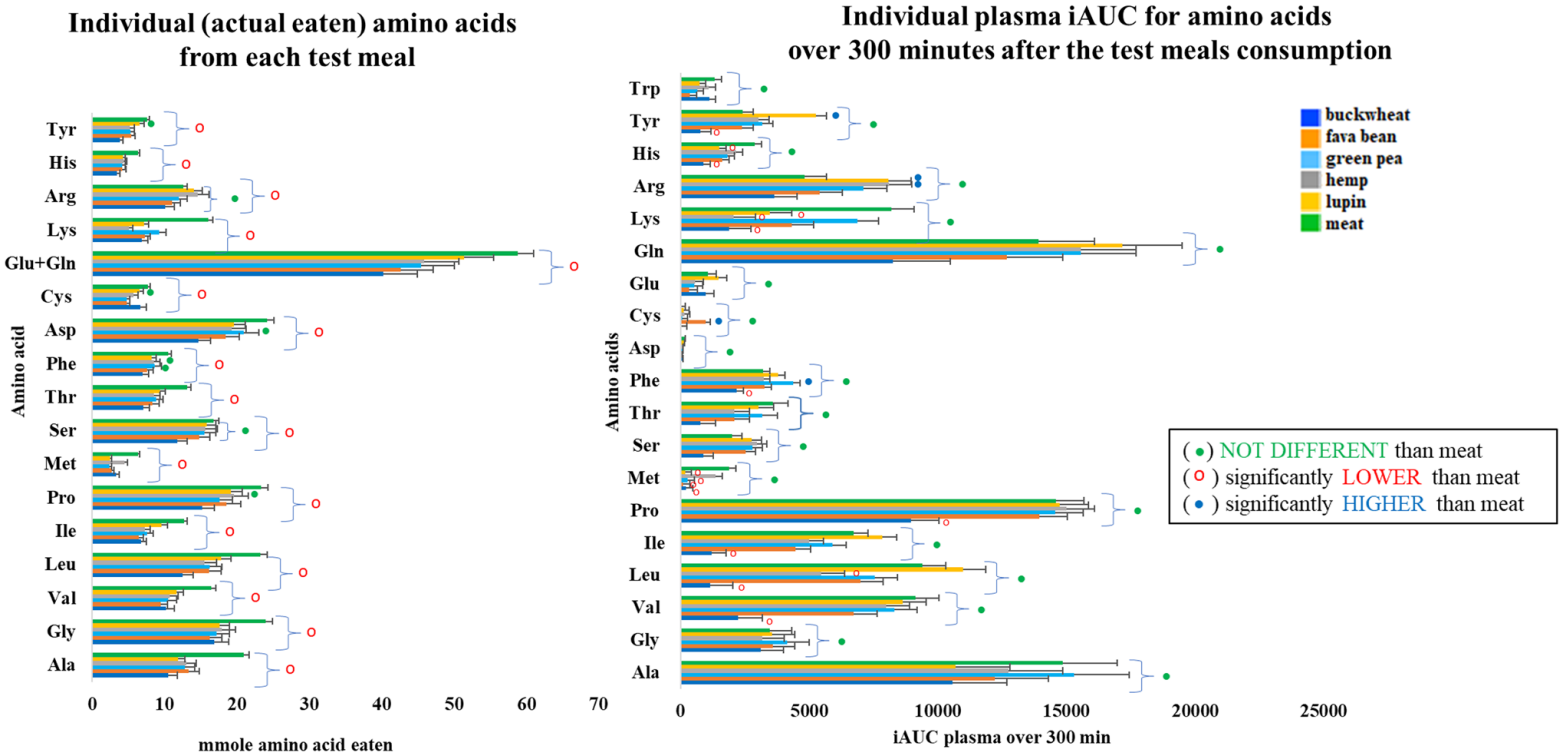
The average (*n* = 10 ± SEM) of individual amino acid eaten by the volunteers from the intervention meals and a statistical comparison (t test) of each amino acid from the individual plant-based meals vs meat meal (LEFT side). The plasma iAUC over 300 min for individual amino acids following consumption of test meals and a statistical comparison (ANOVA) of each amino acid from the individual plant-based meals vs meat meal (RIGHT side)

Overall, the quantity of individual amino acids consumed is significantly higher in the case of the meat meal in comparison with other meals, with very few exceptions where there is no significant difference (Fig. 3). Despite the significantly higher quantity of amino acids being consumed, the amount of individual amino acids measured in plasma over 300 min following meals consumption was not significantly higher for the meat meal compared to the plant-based meals, with few exceptions (see Fig. 3). These included significantly lower levels of His for the fava bean and buckwheat meals, Lys for the fava bean, hemp and buckwheat meals, Tyr, Ile, leu, Phe and Pro for the buckwheat and Met for all meals except hemp (see Fig. 3).

**Fig. 4:**
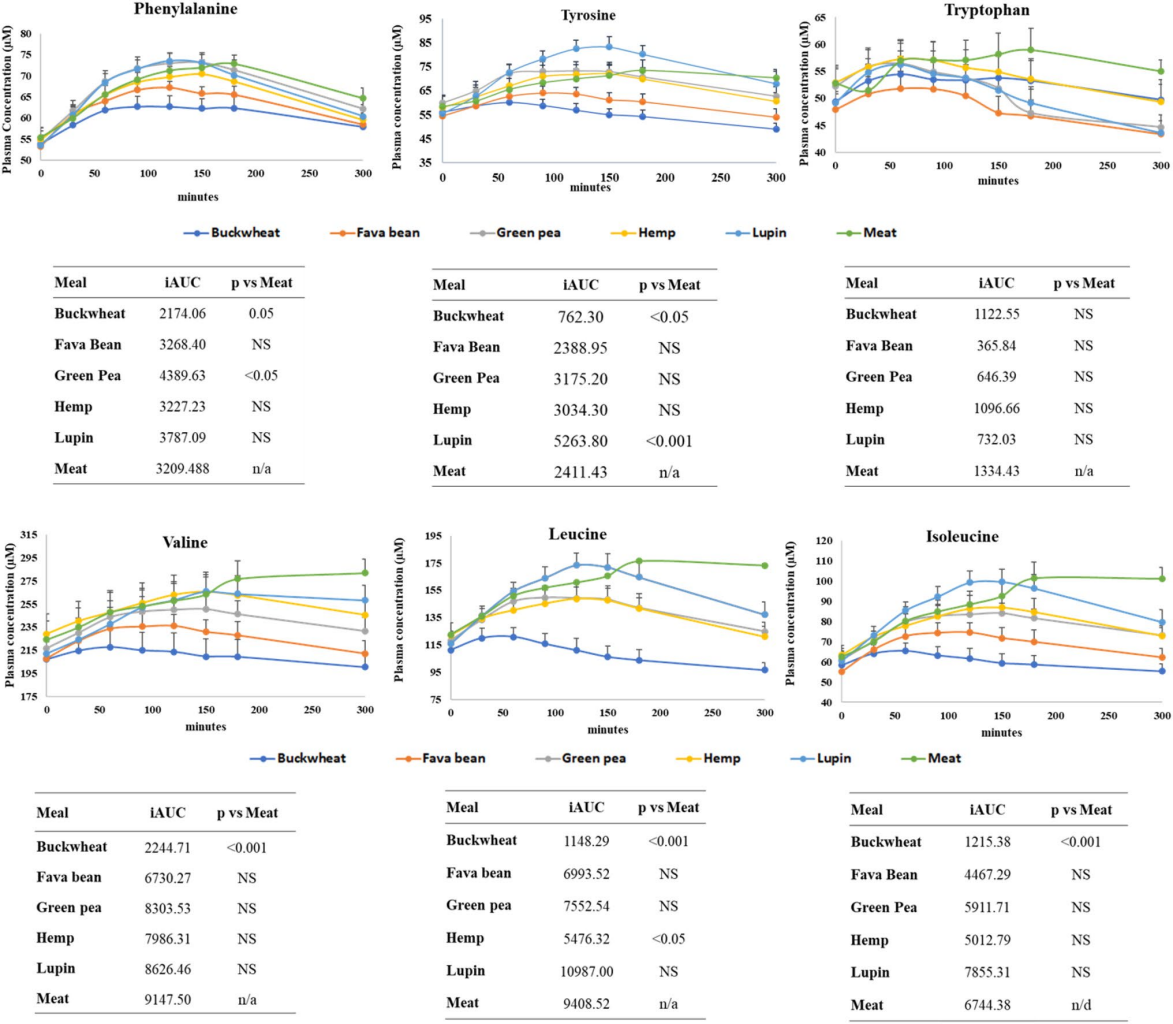
Aromatic amino acids (phenylalanine, tyrosine and tryptophan) and branch chain amino acids (valine, leucine and isoleucine) post-prandial destitutions over 300 min following the consumption of test meals, as average *n* = 10 ± SEM and iAUC of each amino acid from the test meals vs meat meals (ANOVA analysis)

The average (*n* = 10) plasma concentration for each of the amino acids (in nmoles/g plasma) following consumption of test meals at 30, 60, 90, 120, 150, 180 and 300 min together with the AUC and iAUC as well as the plasma amino acid concentrations over 300 min are presented in Table S3 from Online Resource ESM_1. Overall ANOVA found a significant effect of the diet on iAUC for the plasma amino acids with few exceptions such as Ala, Gly, Asp, Glu, Gln, and Trp where no significant effect was found. When ANOVA compared iAUC for meat vs the other plant-based meals, no significant difference was found for the amino acid plasma concentrations over 300 min with the exception of Leu, Met, Asp, Lys, Arg, His and Tyr, where significant differences were found. When ANOVA compared iAUC between the plant-based meals, again significant differences were found in the amino acid plasma concentrations over 300 min with the exception of Ala, Gly, Thr, Asp, Glu, Gln and Trp where no significant differences were found. ANOVA analysis showed that iAUC for the plasma concentrations of all BCAAs (Val, Leu Ile) over 300 min only after consumption of buckwheat meal from the plant-based meals was significantly lower (*p* < 0.001) when compared with plasma distribution after meat meal consumption, exception was iAUC for Leu (*p* < 0.05) after hemp consumption (see Fig. 4). In case of AAAs, ANOVA analysis showed that iAUC for plasma concentrations of Phe was significantly lower after buckwheat (*p* = 0.05) and green pea (*p* < 0.05); Tyr was significantly lower after buckwheat (*p* < 0.05) and lupin (*p* < 0.001) consumption when compared with meat. ANOVA analysis showed no significant differences in Trp plasma concentrations following plant-based meal when compared with meat (Fig. 4).The concentrations of di- and polyamines (spermidine, cadaverine, putresine) as well as tyramine and histamine were found to be significantly increased (*p* < 0.001) in 24 h plasma samples after fava bean meal consumption (Table S5).

The major phenylpropanoid‐derived metabolites have been shown to arise from microbial fermentation of protein [37]. Some of these metabolites have been shown to have beneficial effects for human health, including anti‐oxidant, anti‐inflammatory and anti‐cancer activities [38, 39], however, some of these compounds can be toxic, including acting as co‐carcinogens [40, 41]. On this study, the volunteers were fed high-protein meals, so it was also important to measure the main metabolites derived from the aromatic amino acids to assess potential differences in their plasma concentrations. Moreover, the 24-h plasma sample gives an indication of products produced by microbial metabolism. It was observed that only after consumption of the fava bean meal that there was a significant increase (*p* < 0.001) of phenylpropionic acid, phenylacetic acid and phenylpyruric acid in the 24-h plasma sample suggesting higher levels of microbial metabolism of phenylalanine (Table S4 A, ESM_1). After the consumption of the buckwheat meal, a significant increase (*p* < 0.05) of phenylpropionic acid and phenylacetic acid was observed in plasma at 3- and 5 h (Table S4 A, ESM_1). Again, only after consumption of the fava bean meals was a significant increase (*p* < 0.001) of plasma 4-hydroxyphenylacetic acid, 4-hydroxyphenylpyruric acid, 4-hydroxyphenyllactic acid and *p*-cresol in the 24-h plasma sample observed, indicating high microbial metabolism of tyrosine (Table S4 B, ESM_1). There was a significant effect of diet (*p* = 0.009) and time (0 vs 24 h, *p* = 0.025) on indole-3-propionic acid (IPA). Specifically, there were significant increases in indole-3-propionic acid concentrations in the 24-h plasma after lupin (*p* = 0.028), green pea (*p* = 0.026), and buckwheat (*p* = 0.034) meal consumption (Table S4 C, ESM_1). Following the consumption of the fava bean meal, there was a significant increase (*p* < 0.001) of indole-3-acetic acid, indole-3-carboxylic acid and indole-3-lactic acid in the 24-h plasma sample compared with baseline (Table S4 C, ESM_1). Following the consumption of the meat meal, there was significant increase (*p* < 0.01) of plasma indole-3-carboxylic acid in the 24-h plasma sample and indole-3-pyruvric acid throughout the 5-h postprandial period (Table S4 C, ESM_1).

The highest number of overall plasma metabolites with a significant increase in concentration (within 5 h and mainly at 3 h) was observed following consumption of the buckwheat meal (Table S5). Following the consumption of fava bean meals was observed the highest number of plasma metabolites with a significant change after 24 h (Table S5) suggesting that fava bean meal was metabolised late on GI tract at colon level. Ferulic acid was the metabolite with a significant increase at the 3 h plasma samples following the consumption of all the test meals apart from lupin meal, suggesting that it could be bioavailable from the wheat flour ingredient of the meals. In case of the lupin meals, the concentrations of ferulic acid in plasma at 180 min postprandially is comparable with the concentrations measured in the other test meals, but the increase is not significant (0 vs 180 min).

### Gastrointestinal hormones and their correlation with plasma plant metabolites

**Fig. 5:**
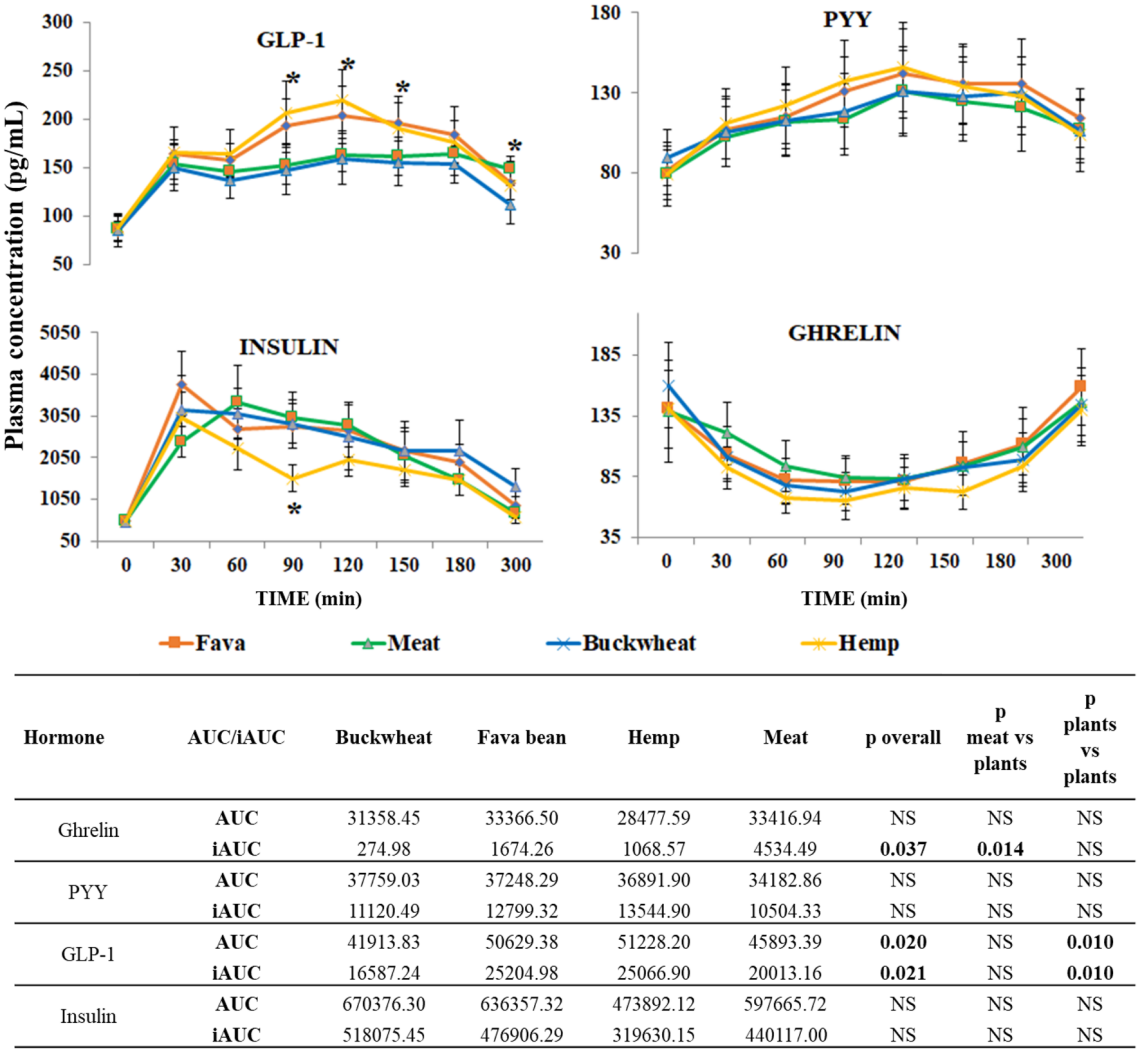
Plasma average concentrations (pg/ml ± SEM), *n* = 10 for ghrelin, PYY, GLP1 and insulin before (0 min) and 30, 60, 90, 120, 180 and 300 min after the fava bean, meat, buckwheat and hemp test meals. (*) GLP1 represents significant plasma concentration differences between fava bean and buckwheat (*p* = 0.017 at 90 min, *p* = 0.050 at 120 min and *p* = 0.037 at 150 min), hemp and buckwheat (*p* = 0.007 at 90 min, *p* = 0.011 at 120 min, and *p* = 0.051 at 150 min), and meat and buckwheat (*p* = 0.005 at 300 min). (*) Insulin represents significant plasma concentration differences at 90 min between hemp and buckwheat (*p* = 0.009), fava bean (*p* = 0.005) and meat (0.0122). AUC and iAUC for the gut hormones for the test meals for 300 min, overall ANOVA *p* values and ANOVA *p* values for the meat vs plants meals and plants vs plants meals

Overall ANOVA analysis found no significant effects of the test meals on the ghrelin plasma concentration for AUC (*p* = 0.266), and mild significance for iAUC (*p* = 0.037). ANOVA found no significant changes in ghrelin concentration with time and between plant-based meals. However, following consumption of the hemp meal, the lowest ghrelin concentrations (lowest AUC) in the volunteers’ plasma was recorded and a significant effect on iAUC (*p* = 0.014, ANOVA) when plant-based meals vs meat meal were compared (Fig. 5).

Overall ANOVA showed no significant time effect on PYY (*p* = 0.753), and no diet effect (*p* = 0.793), AUC (*p* = 0.400) and iAUC (*p* = 0.325). ANOVA analysis also found no significant changes in PYY concentration with time. After consumption of the hemp meal, plasma PYY had the lowest AUC in average and after consumption of buckwheat meal had the highest AUC amongst all the test meals (Fig. 5).

For GLP-1, overall ANOVA analysis found significant effect of diet (*p* = 0.0045) and time (*p* < 0.001) and between diet effects (*p* = 0.0120), with buckwheat vs hemp (*p* = 0.049) and buckwheat vs fava bean (*p* = 0.011). Significant effect on overall AUC iAUC (*p* = 0.02). ANOVA analysis found significant plasma concentration differences between the fava bean and buckwheat meals (*p* = 0.017 at 90 min, *p* = 0.050 at 120 min and *p* = 0.037 at 150 min), hemp and buckwheat meals (*p* = 0.007 at 90 min, *p* = 0.011 at 120 min, and *p* = 0.051 at 150 min), and the meat and buckwheat meals (*p* = 0.005 at 300 min). There were significant effects on AUC and iAUC (*p* = 0.01, ANOVA), with the AUC (51,228.20) after the hemp meal being significantly higher (Fig. 5).

Overall ANOVA analysis found a time effect (*p* = 0.007) for insulin but no effect on AUC and iAUC (*p* = 0.157 and *p* = 0.173). ANOVA analysis found a mild diet effect at 30 min (*p* = 0.048) and significant insulin plasma concentration differences at 90 min. Insulin concentration after the hemp meal was significantly lower when compared with the buckwheat (*p* = 0.009), fava bean meal (*p* = 0.005) and meat (*p* = 0.012) meals and had the lowest, but not significant, AUC and iAUC.

**Fig. 6:**
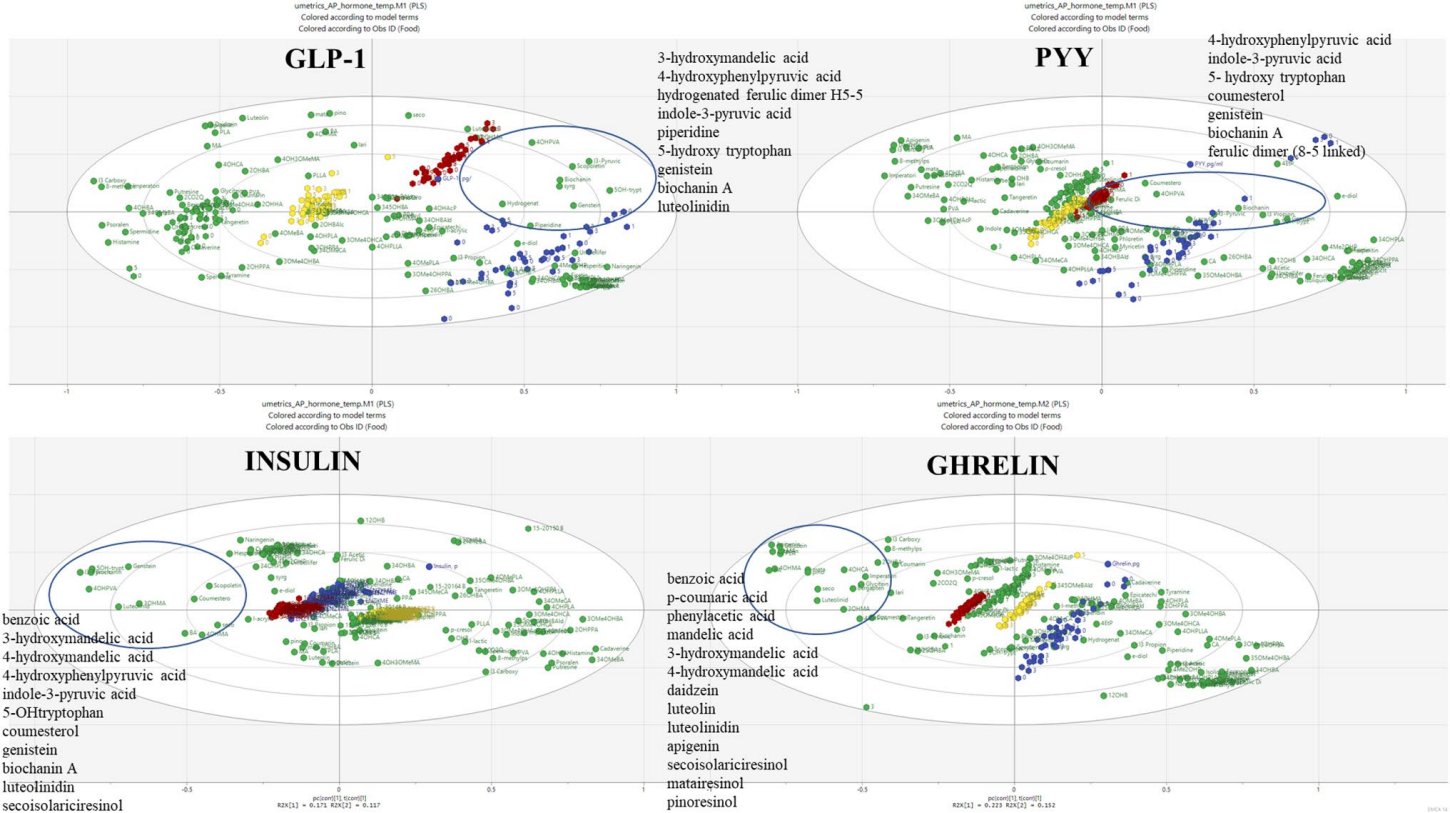
PLS DA analysis of each gastrointestinal hormone and the plant metabolites measured in plasma using LCMS/MS over 300 min highlighting in each case the group of metabolites identified as associated with the effect of hemp meal on the gut hormones

PLS DA analysis highlighted several plasma metabolites potentially associated with the gastrointestinal hormones measured (Fig. 6 and Tables S6 A–D). Plasma 4-hydroxyphenylpyruvic acid, indole-3-pyruvic acid, 5-hydoxy tryptophan, genistein and biochanin A were present in significantly higher amounts (*p* < 0.001) after hemp consumption (at 1 and 3 h) compared with meat and were positively associated with increased GLP-1 and PYY and negatively associated with increased insulin. Plasma 3-hydroxymandelic acid and luteolidin were positively associated with the effect of hemp on GLP-1 and ghrelin.

Secoisoresinol, benzoic acid, 4-hydroxy mandelic acid were found to be present in significantly higher amounts (*p* < 0.001) after hemp consumption (at 1 and 3 h) when compared with meat and were positively associated with the decrease of insulin and ghrelin (Tables S6 A–D). Similarly, significantly higher plasma concentrations (*p* < 0.001) of coumestrol were observed following hemp consumption when compared with meat and was associated with increased PYY and decreased Insulin concentrations in plasma. Furthermore, *p*-coumaric acid, daidzein, matairesinol (*p* < 0.01), phenyl acetic acid, luteolin, apigenin, and pinoresinol (*p* < 0.001) were detected at significantly higher amounts in plasma at 1 and 3 h after consumption of the hemp *vs* meat meals and were associated with decreased ghrelin.

### Plasma lipids, glucose, urea and homocysteine

There was no significant diet effect on HDL (*p* = 0.359) and LDL (*p* = 0.280) when analyzed with ANOVA, there was no significant difference between meals for the AUC and iAUC values for HDL, *p* = 0.865, and *p* = 0.291, respectively (ANOVA). There was no significant difference between meals for the AUC, and iAUC values for LDL, *p* = 0.064, and *p* = 0.298, respectively (ANOVA). Overall ANOVA analysis found significant diet and time effect on postprandial plasma NEFA (*p* < 0.001, ANOVA) over 300 min, plasma concentrations being significantly higher after consumption of hemp and lupin meals than other test meals. ANOVA found significant effect of diet on AUC for NEFA (*p* < 0.001, ANOVA), AUC for hemp and lupin meals being significantly higher than rest of the test meals and no significant effect on iAUC (*p* = 0.055). Significant effect on NEFA concentration at 90 min (*p* = 0.005, ANOVA), NEFA plasma concentration after the hemp meals was significantly higher than after meat and fava bean meal, and NEFA plasma concentration after the lupin meal were significantly higher than after the green pea, fava bean and meat meals; NEFA plasma concentration after the lupin and hemp meals was significantly higher than after the green pea, fava bean, buckwheat and meat meals at 120 min (*p* = 0.004, ANOVA), at 150 min (*p* < 0.001, ANOVA) and 180 min (*p* < 0.001, ANOVA); at 300 min, NEFA plasma concentration after the lupin meal were significantly higher than after the green pea, fava bean and buckwheat meals and NEFA plasma concentration after the hemp meal was significantly higher than the buckwheat and green pea meals (*p* = 0.018, ANOVA). Overall ANOVA analysis found no significant diet effect on triglycerides (*p* = 0.060), AUC (*p* = 0.409) but a significant time effect (*p* < 0.001) and a mild effect on iAUC (0.041), significantly higher after the lupin meal iAUC (174.0) in comparison with fava bean, meat and buckwheat meals. The hemp and lupin meals are the highest in fat, however, we consider that the supplementation of the diet with these meals which are also high in insoluble fibre (NSP) should be done for a prolonged period in order to see any effect of this nutrient or their metabolites on the metabolic health biomarkers such as NEFA.

Overall ANOVA analysis found significant effects on diet and time on plasma glucose concentrations (*p* < 0.001), these being significantly higher after consuming of the buckwheat meal ANOVA analysis found a significant effect of the test meals on AUC and iAUC for glucose (*p* = 0.010, *p* = 0.032, respectively), AUC after the buckwheat meal was significantly higher than all other meals, iAUC was significantly higher after the buckwheat meal in comparison with the green pea, meat and hemp meals. After consumption of the buckwheat meal, plasma concentrations for glucose were significantly higher at 60 min (*p* = 0.015, ANOVA), when compared with the lupin, hemp, green pea, and meat meals; at 120 min (*p* = 0.028, ANOVA), when compared with all the other meals; and at 150 min (*p* = 0.001, ANOVA), when compared with the hemp, fava bean, green pea and meat meals.

Overall ANOVA analysis found a significant diet (*p* < 0.010) and time (*p* < 0.001) effect on postprandial plasma urea over 300 min. ANOVA analysis found no significant effect of test meals on AUC and significant effect for iAUC for urea (*p* = 0.091, *p* = 0.035, respectively), iAUC was significantly higher after the meat and lupin meals in comparison with the green pea, fava bean and buckwheat meals. After consumption of the lupin meal. plasma urea concentrations were significantly higher at 150 and 180 min (*p* = 0.047, *p* = 0.015, respectively ANOVA), when compared with the buckwheat, fava bean and green pea meals; and at 300 min (*p* < 0.001, ANOVA), plasma concentration of urea were significantly higher after consumption of the meat meals in comparison with the buckwheat, fava bean, green pea, hemp meals.

Overall ANOVA analysis found significant diet and time effect on postprandial plasma HCys (*p* < 0.001) over 300 min, plasma HCys concentrations being significantly higher after the fava bean meal in comparison with all the other test meals. ANOVA found no significant effect of test meals on AUC and significant effect for iAUC for urea (*p* = 0.536, *p* < 0.001, respectively), iAUC was significantly higher after the fava bean meals in comparison with all the other meals. After consumption of the fava bean meals, plasma HCys concentration was significantly at 300 min (*p* = 0.031, ANOVA) when compared with all the other test meals.

### Satiety and hunger

**Fig. 7:**
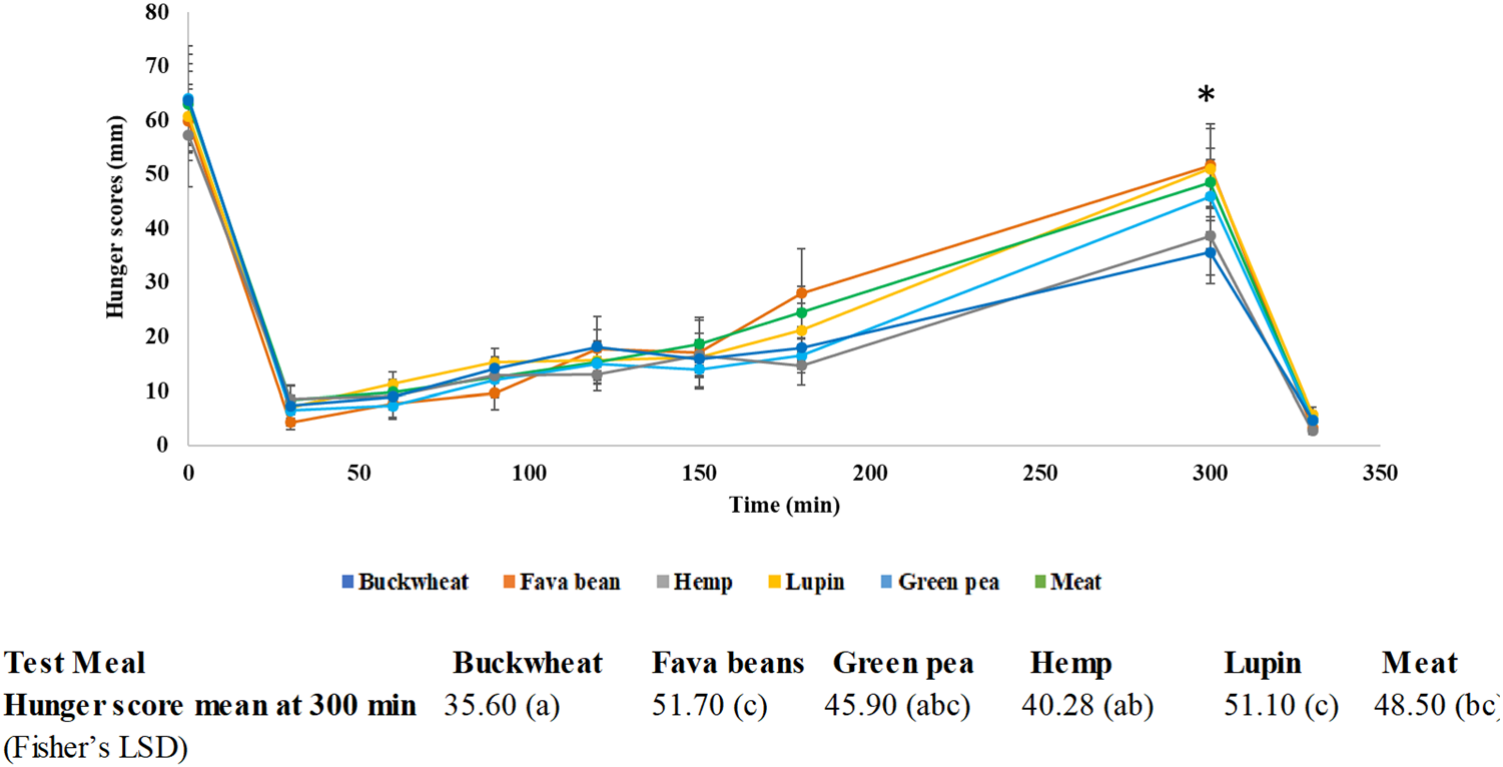
Average hunger score (mm ± SEM), *n* = 10, before (0 min) and 30, 60, 90, 120, 180 and 300 min after the test meals and at 330 min (after the ad libitum) lunch. ANOVA Fisher’s LSD analysis for hunger mean scores at 300 min after the test meals. (*) represents significant differences (*p* = 0.018) for the hunger scores between test meals

Significant differences were found for all the VAS measures (hunger, fullness, desire, quantity, thirst, preoccupation) with time, from baseline to post-lunch (*p* < 0.001), data not shown. There were no significant differences between the intervention meals for most of the variables measured. The largest and only significant difference between the intervention meals occurs with hunger at 300 min (*p* = 0.018), see Fig. 7. Hunger score was significantly lower at 300 min after the buckwheat meal (35.60) vs meat meal (48.50), lupin meal (51.10) and fava bean meal (51.70). There was no significant difference in consumption of ad libitum lunch (*p* = 0.977). The results showed mild significance (*p* = 0.04) for the effect of protein (with a coefficient of − 2.08) for the hunger VAS values for all intervention meals when adjusted for protein intake (using consumed protein amount as a covariate with ANCOVA). The source of the protein did not show significant difference for inducing increased satiety (*p* = 0.434).

## Discussion

The present study evaluates postprandial events related to satiety and protein metabolism following acute consumption of high-protein meals, comparing meat with five different sources of plant protein. The test meals were designed to deliver the same amount of protein (30 g) for each intervention. The test meals were found to be significantly different in terms of amino acid (as previously showed by ANOVA Fisher’s test analysis), fat and non-starch polysaccharides (presented on ESM). The test meals also had substantially different composition in terms of non-nutrient metabolites with clear segregation observed by principal component analysis for the buckwheat and hemp meals compared to the rest of the test meals. In general, the volunteers consumed significantly higher amounts of most of the individual amino acids from the meat meal, compared to the plant-based meals, the postprandial concentrations of these amino acids were not significantly higher. For the following amino acids: serine (Ser), glycine (Gly), aspartic acid (Asp), glutamic acid (Glu), threonine (Thr), alanine (Ala), valine (Val), tryptophan (Trp), glutamine (Gln) amount eaten does not reflect their postprandial availability suggesting that these plant-based sources could contribute towards daily requirements. This is important since Val, Thr and Trp are essential amino acids. Moreover, fava bean meal delivers significantly higher amounts of cysteine (Cys) postprandially compared to meat meal and lupin and significantly higher amounts of tyrosine (Tyr) compared to meat despite the volunteers having consumed the same or significantly lower amounts of these amino acids from these plant-based meals. Both the hemp and meat meals delivered similar amounts of Met postprandially again despite significantly lower levels of Met consumption from the hemp meals. These results demonstrate differences in the bioavailability of amino acids suggesting an important impact of the food matrix in plant-based compared to meat meals.

Amino acid profiles are emerging as novel biomarkers for predicting the risk of developing T2D [42]. Branched-chain amino acids (BCAAs) and to a lesser extent, the aromatic amino acids (AAAs); phenylalanine and tyrosine were associated with insulin resistance in men but not in women in the Young Finns Study [5]. The gluconeogenic amino acids (alanine, glutamine, or glycine) and several other amino acids (histidine, arginine, and tryptophan) did not show an association with insulin resistance. High plasma concentrations of BCAAs, regardless of the dietary source, have been associated with a high risk of developing T2D [43]. High dietary intakes of BCAAs was linked to T2D only when the blood levels for the BCAAs were observed to be high. This suggests that dietary protein rich in BCAAs may not cause T2D, however, nutritional strategies that help maintain a lower concentration of these amino acids could be used in T2D prevention. Therefore, assessing bioavailability from food sources is critical as this study showed that even though the volunteers consumed significantly higher amounts of BCAAs from the meat meal compared with all the plant-based meals, but postprandial availability was only lower for all BCAAs following buckwheat meal consumption and for Leu following hemp meal consumption (when compared with meat).

The results also showed that both phenylalanine and tyrosine iAUC are significantly lower following consumption of the buckwheat meal. In this case, the phenylalanine content of buckwheat was not significantly different from the other meals except for green pea. In the case of tyrosine, buckwheat was the only meal to have lower amounts than meat, whereas, tyrosine was not significantly different, yet in plasma after lupin consumption, tyrosine was found in significantly higher amounts. High concentrations of valine, leucine, isoleucine, phenylalanine, tyrosine, alanine, glutamate, ornithine, and lysine were associated with an increased risk of incident T2D, in a linear manner in a 5-year follow-up study [42]. The same study reported that high glutamine concentrations were associated with a decreased risk of T2D. Our results showed no significant differences in plasma glutamine when all meals were compared. It is very likely that the increased blood levels of BCAAs act as a marker of insulin dysfunction, but may not be the cause.

The present study also found significant increases in indole-3-propionic acid (IPA) concentrations in the 24-h plasma samples following consumption of the lupin, green pea and buckwheat meals. IPA produced from dietary tryptophan has also been found in cerebrospinal fluid [44, 45]. Recently, IPA was associated with a lower likelihood of developing T2D in people with impaired glucose tolerance [7]. The effect of IPA on lowering T2D risk might be mediated by direct effect of IPA on β-cell function [46]. In animal studies, when IPA was fed to rats for 6 weeks, fasting blood glucose and plasma insulin levels were lowered [47]. Therefore, foods that increase the circulatory concentration of IPA are potential candidates for the treatment of metabolic disorders associated with insulin resistance.

Indole 3-pyruvic acid and another tryptophan metabolite; 5-hydroxytryptophan (5-HTP) were present in significant amounts in plasma at 1 and 3 h following hemp meal consumption (compared with meat) and positively associated with GLP-1 (PLS-DA analysis). 5-HTP has been shown to counteract hunger-inducing hormones, working to suppress appetite [48, 49]. Hemp meal consumption increased the plasma concentration of GLP-1 at 90, 120, 150, and 300 min. It has been also shown that indole, another tryptophan metabolite is able to modulate the secretion of glucagon-like peptide-1 (GLP-1) from immortalized and primary mouse colonic L cells [11]. The beneficial effect of hemp on GLP-1 makes it an attractive ingredient for nutritional therapies to support T2D management. Other metabolites found in significantly higher concentrations in plasma following hemp consumption (compared with meat) included benzoic acid, 4-hydroxypyruvic- acid, 4-hydroxy and 3-hydroxy mandelic acids, genistein, luteolidin and biochanin A and these were also associated (PLS-DA analysis) with the gastrointestinal hormones modulation (increased GLP-1 and PYY and decreased insulin and ghrelin). Similarly, secoisoresinol, also present in significant amount in plasma following hemp consumption was associated (PLS-DA) with reductions in insulin and ghrelin. To our knowledge, there is no previous study linking these metabolites to the gastrointestinal hormones. Furthermore, from the test meals, hemp had the highest fibre and total fat content. Nutrients, including glucose, fatty acids, and dietary fiber, are known also to upregulate the transcription of the gene encoding GLP-1 [18].

The plant-based meals also delivered significant amount of some other phytophenols including high levels of ferulic (all meals except lupin) and salicylic acid (buckwheat and hemp). These phytophenols were previously shown to have anti‐inflammatory activity [50] and to inhibit peroxidase and lipoxygenase enzymes (salicylic acid) [51, 52]. Targeting inflammation also constitutes a potential strategy in the prevention and treatment of chronic diseases such as T2D and CVD [53]. Antioxidants are also known to reduce complications associated with T2D, induced by the oxidative stress by delaying glucose absorption [54, 55]. Salicylates have also been shown to improve glucose tolerance and hyperglycemia in individuals both with and without T2D [56] and clinical studies have confirmed that salicylates reduce fasting glucose and improve systemic glucose concentrations [57]. The additional presence of these molecules in plasma following consumption of test meals and in particular, buckwheat and hemp could promote these foods for chronic disease prevention and as their potential for developing functional food ingredients.

Meta-analysis of observational studies suggest that elevated homocysteine is an independent predictor of ischemic heart disease and stroke in healthy people [58] and associated with insulin resistance in patients with diabetes [59]. In addition, high circulatory levels of homocysteine could lead to osteoporosis and rheumatism, as well as neuronal pathologies, including Alzheimer's and Parkinson's diseases [60]. The present study showed significant effect on diet and time and between diets on homocysteine with buckwheat meal consumption delivering the lowest concentrations and fava bean meal was the highest of circulatory homocysteine. This suggests that buckwheat could help maintain low circulating levels of homocysteine. Treatments aiming to decrease homocysteine blood levels, have shown a potential effect in preventing stroke [61], therefore, foods able to decrease the circulatory levels of homocysteine, such as buckwheat, should be considered nutritional strategies to prevent pathologies such CVD and T2D. We also observed a trend on the postprandial plasma urea concentrations, as lupin and meat meals were increasing the plasma urea concentration with the buckwheat having an opposite effect. These findings are important as research shows that higher levels of urea may increase insulin resistance and suppress insulin secretion [62]. The beneficial effect of buckwheat on plasma urea should, therefore, be explored further.

The consumption of buckwheat test meal results in a steadier release (within normal physiological clinical response) of the postprandial glucose (with not significantly different 30 min peak and returning to initial values at 300 min). This could be because buckwheat flour contains, in general, a high starch content. A proportion of the starch becomes hydrolysis resistant during baking of the groats and flour. Therefore, heating of buckwheat flour results in the formation of indigestible starch or slowly digestible starch. Consumption of foods with high slowly digestible starch may result in a moderate release of glucose from the intestine into the blood. However, we did not observe any significant effect on GLP-1 or Insulin following the buckwheat meal consumption.

This study also assessed the satiety following the consumption of the six different high-protein meals. As mentioned before, apart from the protein content, the macronutrients and mineral and non-nutrient composition of the test meals were different (including the size of the meals). Since the protein content of the raw materials differed, the quantity used to deliver the same amount of protein in each meal varied. Results showed that the palatability of the plant-based test meals were reduced, and the volunteers consumed smaller proportions of those meals *vs* the meat meal (in highest quantities the meat and lupin test meals). Because of this consumption trend, the protein intake was therefore higher for the meat vs plant-based meals. However, the self-reported satiety was similar for all the meals. Furthermore, buckwheat seems to significantly increase satiety (*p* = 0.018) compared to the control, providing a feeling of fullness for longer, despite the decreased consumption of protein. The Fisher’s LSD analysis for hunger at 300 min also demonstrated that buckwheat meal was the only meal to differ from the meat meal. There was only a mild significance (*p* = 0.04) for the effect of protein for the hunger (VAS values) for all intervention meals (when adjusted for protein intake) and was independent of protein source (*p* = 0.434). Previous research showed that there were no significant differences in hunger scores between meat-based meals and plant-based meals with the same content of protein, fat and carbohydrates [12, 63]. However, some researchers showed higher satiety responses to consuming plant vs animal protein [64]. Since we only matched the meals on protein content, our results suggest that the amount of protein might not be the only component responsible for the observed satiety; otherwise lupin and meat meals would have produced higher satiety values compared to the hemp and buckwheat meals. There are probably other macronutrients that in combination with the protein could lead to an increase in satiety, most likely dietary fibre [65]. Among the test meals, hemp was the richest source of fibre (NSP), (24.4 g/portion, *p* < 0.001), this or the combination of protein and fiber could explain the low hunger scores recorded for the hemp meal, as reported by previous research [66].

### Study limitations

The present study has several limitations. A relatively small sample size of ten participants limits the conclusions that can be drawn from the study, suggesting the importance to replicate our findings in a larger sample size, however, this modest sample size does not weaken the significant differences which have been found. Additionally, the order the meals were consumed was not randomized, however, the interventions were properly spaced out (minimum 2 weeks between each) and, therefore, we do not estimate any carry over between treatments. Another limitation is that the volunteers were not consuming the entire test meals, but our analysis accounted for the varying protein intake by including a term for amount consumed.

## Conclusion

This work is the first comprehensive study assessing postprandial bioavailability of plant metabolites and protein from buckwheat, fava bean, green pea, hemp, lupin compared to meat. The results obtained promote plant-based foods as a good source of alternative protein to meat. The results also suggest that the postprandial amino acid profiles are not substantially affected by the source of protein, implying that there is a matrix effect impacting on postprandial availability. It is important that food/protein sources are not just judged on amino acid composition and that dietary contribution also considers the plant matrix effect on the availability of the amino acids. To our knowledge, this is also the first clinical evidence that: hemp-rich meals reduce postprandial insulin and increase GLP-1 in healthy human volunteers; furthermore hemp was considered the best source to decrease ghrelin concentration in plasma. The buckwheat meal was found to be best at reducing hunger and promoting satiety, as well as delivering the lowest levels of circulatory BCAAs. The study also identified a group of plant metabolites, specifically indole 3-pyruvic acid, 5-hydroxytryptophan, benzoic acid, 4-hydroxypyruvic acid, 4-hydroxymandelic acid, 3-hydroxy andelic acids, genistein, luteolidin, biochanin A, and secoisoresinol, which could be responsible for the hemp meal that could be responsible for the gastrointestinal hormone response.

The present work brings strong evidence that plants are viable sources of dietary protein and strengthens the current recommendations of increasing the consumption of plant-based foods.

## Supplementary Information

Below is the link to the electronic supplementary material.

Supplementary file1 (DOCX 583 KB)

## Data Availability

Yes.
