# Supplementary material for: Hemp and buckwheat are valuable sources of dietary amino acids, beneficially modulating gastrointestinal hormones and promoting satiety in healthy volunteers
Source: Eur J Nutr. 2021 Oct 30;61(2):1057–72. doi: 10.1007/s00394-021-02711-z (PMC8854285; doi:10.1007/s00394-021-02711-z)
Supplement: Supplementary file 1 — Supplementary file1 (DOCX 583 KB) [file 394_2021_2711_MOESM1_ESM.docx]

**Electronic Supplementary Material (ESM_1)**

**Hemp and Buckwheat are valuable sources of dietary amino acids, beneficially modulating gastrointestinal hormones and promoting satiety in healthy volunteers**

**Madalina Neacsu, Nicholas J. Vaughan, Salvatore Multari, Elisabeth Haljas, Lorraine Scobbie, Gary J. Duncan, Louise Cantlay, Claire Fyfe, Susan Anderson, Graham Horgan, Alexandra M. Johnstone and Wendy R. Russell**

The Rowett Institute, University of Aberdeen, Aberdeen, Scotland, UK, AB25 2ZD (MN, NJV, EH, SM, LS, GJD, LC, CF, SA, AMJ, WRR)

Biomathematics and Statistics Scotland, Aberdeen, Scotland, UK, AB25 2ZD (GH)

Corresponding author: Dr Madalina Neacsu, The Rowett Institute, Aberdeen, Scotland, UK, AB25 2ZD; E-mail: M.Neacsu@abdn.ac.uk; Fax +44 (0)1224 438698; Telephone +44 (0)1224 438760

**Table S1:** The test meals (A) and *ad libitum* (B) ingredients

| **A) Bread Rolls Ingredients** | |  | |  |  |  |  |  |  |  |  |  |  |
| --- | --- | --- | --- | --- | --- | --- | --- | --- | --- | --- | --- | --- | --- |
| **(g)** | | **Buckwheat** | | **Fava Bean** | | **Green Pea** | | **Hemp** | | | **Lupin** | **Wheat** | |
| **Bicarbonate soda** | | 2 | | 1 | | 2 | | 2 | | | 2 | 1 | |
| **Butter no added salt** | | 18 | | 18 | | 18 | | 18 | | | 18 | 9 | |
| **Quick Yeast** | | 3.2 | | 1.6 | | 3.2 | | 2.2 | | | 3.2 | 1.6 | |
| **Salt** | | 3 | | 1.5 | | 3 | | 3 | | | 3 | 1.5 | |
| **Sugar** | | 3 | | 1.5 | | 3 | | 3 | | | 3 | 1.5 | |
| **Warm Water** | | 150 | | 60 | | 120 | | 115 | | | 120 | 60 | |
| **Tesco Strong White Flour** | | 125 | | 125 | | 125 | | 125 | | | 125 | 125 | |
| **Plant protein flour** | | 100 | | 150 | | 136.5 | | 85.5 | | | 75 |  | |
| **Buckwheat grain** | | 66.5 | |  | |  | |  | | |  |  | |
| **Macronutrient content*** | | **Buckwheat** | | **Fava Bean** | | **Green Pea** | | **Hemp** | **Lupin** | | | **Meat** | |
| **Fat** | | 6 | | 4.9 | | 4.7 | | 7.88 | 8.62 | | | 6.87 | |
| **Protein** | | 12.19 | | 12.06 | | 13.32 | | 15.1 | 15.62 | | | 17.86 | |
| **Carbohydrate** | | 60.24 | | 49.72 | | 54.15 | | 47.75 | 35.66 | | | 36.98 | |
| **Fibre** | | 8.18 | | 10.31 | | 11.72 | | 7.49 | 8.32 | | | 1.6 | |
| **Saturated Fat** | | 2.5 | | 2.51 | | 2.81 | | 3.09 | 3.6 | | | 3.24 | |
| **Polyunsaturated Fat** | | 0 | | 0 | | 0 | | 0 | 1.82 | | | 0 | |
| **Sugars** | | 0 | | 0 | | 0 | | 0 | 0.91 | | | 0 | |
| **Starch** | | 2.29 | | 1.3 | | 4.21 | | 1.69 | 4.11 | | | 1.34 | |

* Macronutrient content of the intervention meals (in g/100g), obtained using McCance and Widdowson's food composition tables in NetWISP

| **B) *Ad libitum* meal ingredients** | **Weight (g)** | **Energy (kcal)** |
| --- | --- | --- |
| **Carrots, old, boiled in unsalted water** | 45 | 10.8 |
| **Beef, mince, stewed** | 59 | 122.8 |
| **Garlic puree** | 4.5 | 16.9 |
| **Oregano, dried, ground** | 0.7 | 2.1 |
| **white sugar** | 1.3 | 5.2 |
| **Beef stock cube** | 4.1 |  |
| **Basil, dried, ground** | 0.5 | 1.3 |
| **Onions, boiled in unsalted water** | 54.2 | 9.5 |
| **Peppers, capsicum, green, boiled in salted water** | 53 | 9.6 |
| **Chilli powder** | 0.6 | 0 |
| **Olive oil** | 13.9 | 122.9 |
| **Tomatoes, canned, whole contents** | 270 | 44.6 |
| **Baxter's Tomato Chutney** | 89 | 137.1 |
| **Lemon juice, fresh** | 2 | 0.1 |
| **Macaroni, boiled (penne)** | 363 | 317 |
| **TOTAL** | **960.8** | **800** |

**Results:**

**A**


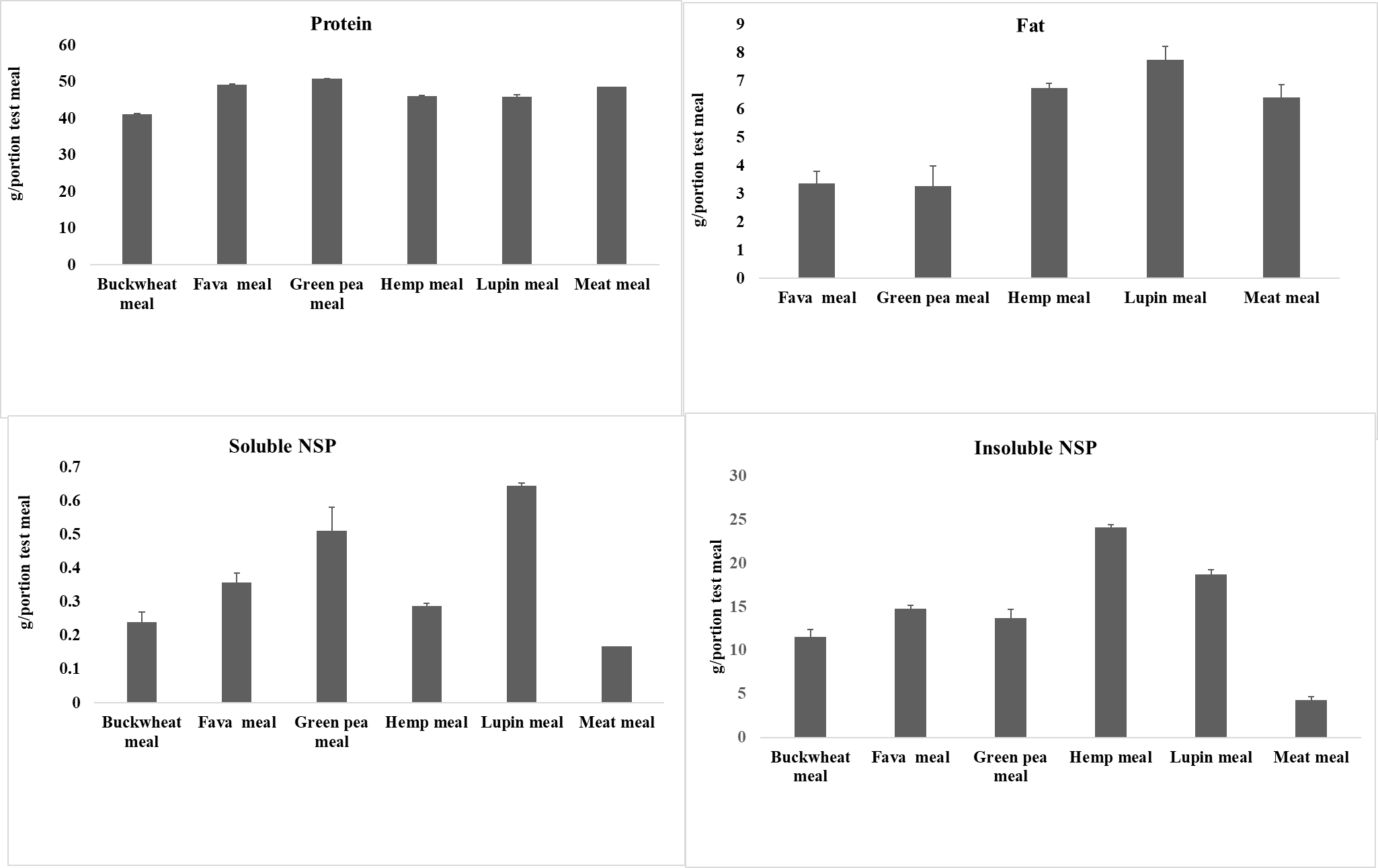


**B**

**D**

**C**

**Fig S 1 A, B, C, D** The macronutrient composition of the study test meals, the protein (A), fat (B), soluble (C) and non-starch polysaccharide (D) (soluble and insoluble) content (NSP)

**A**

**
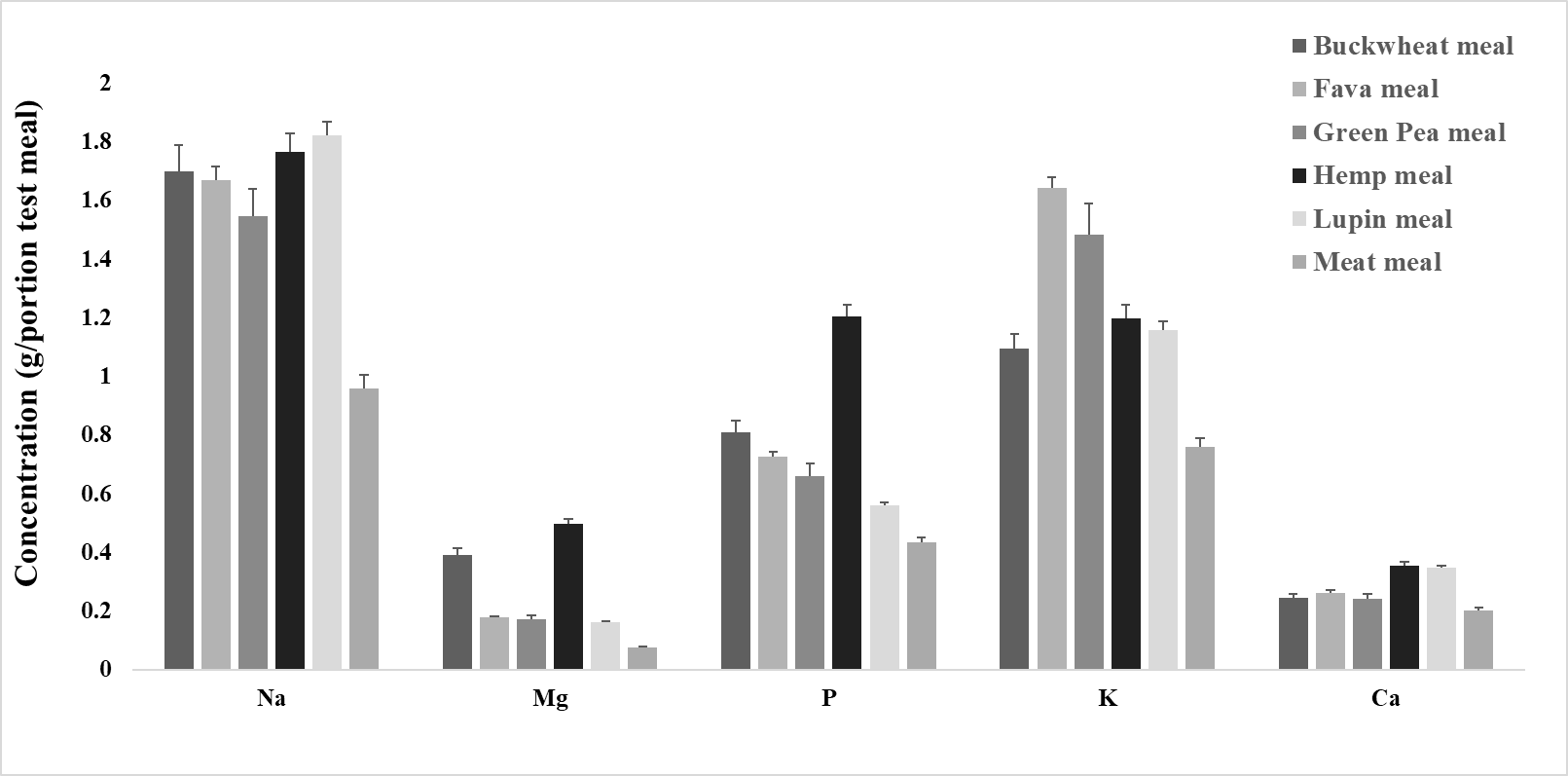
**

**
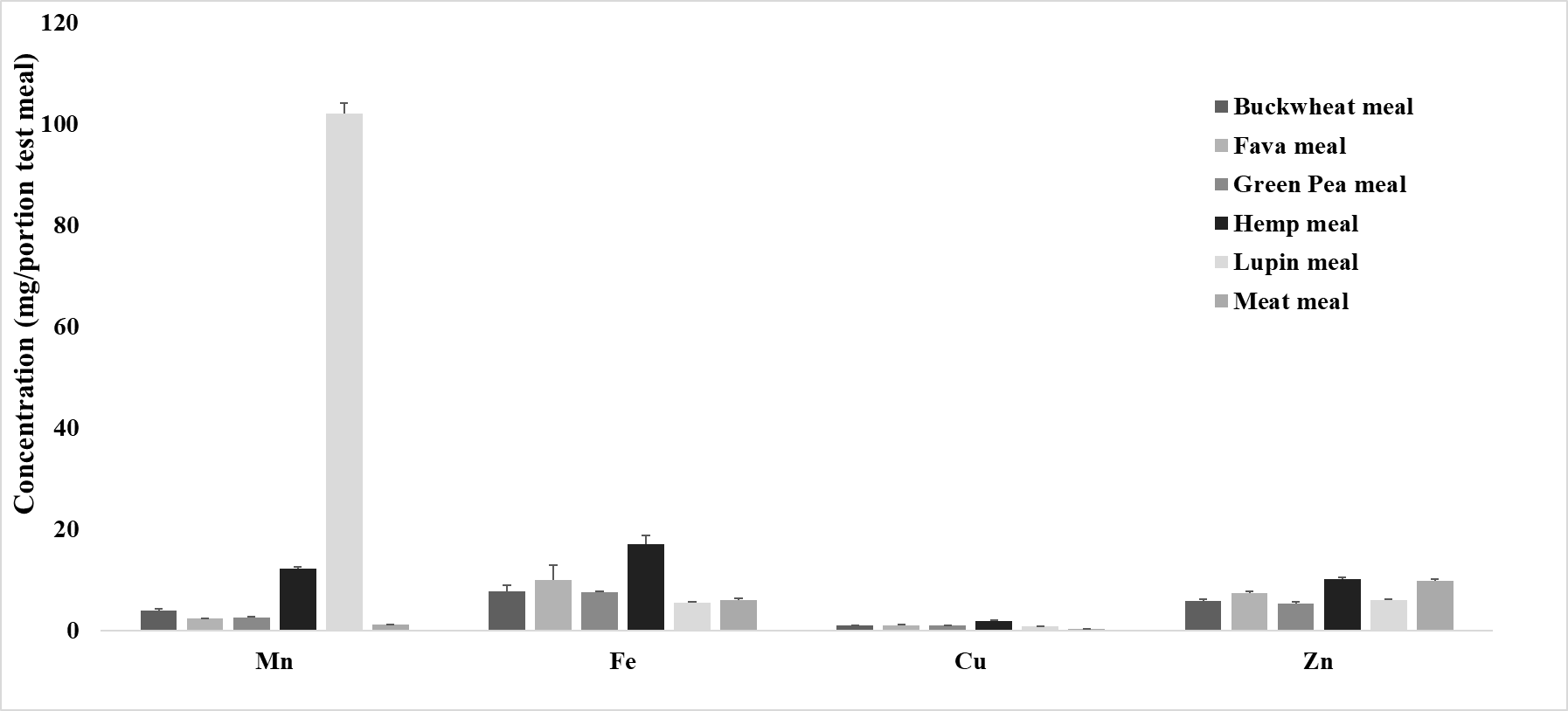
**

**B**

**
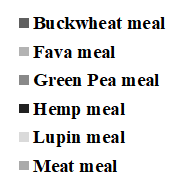

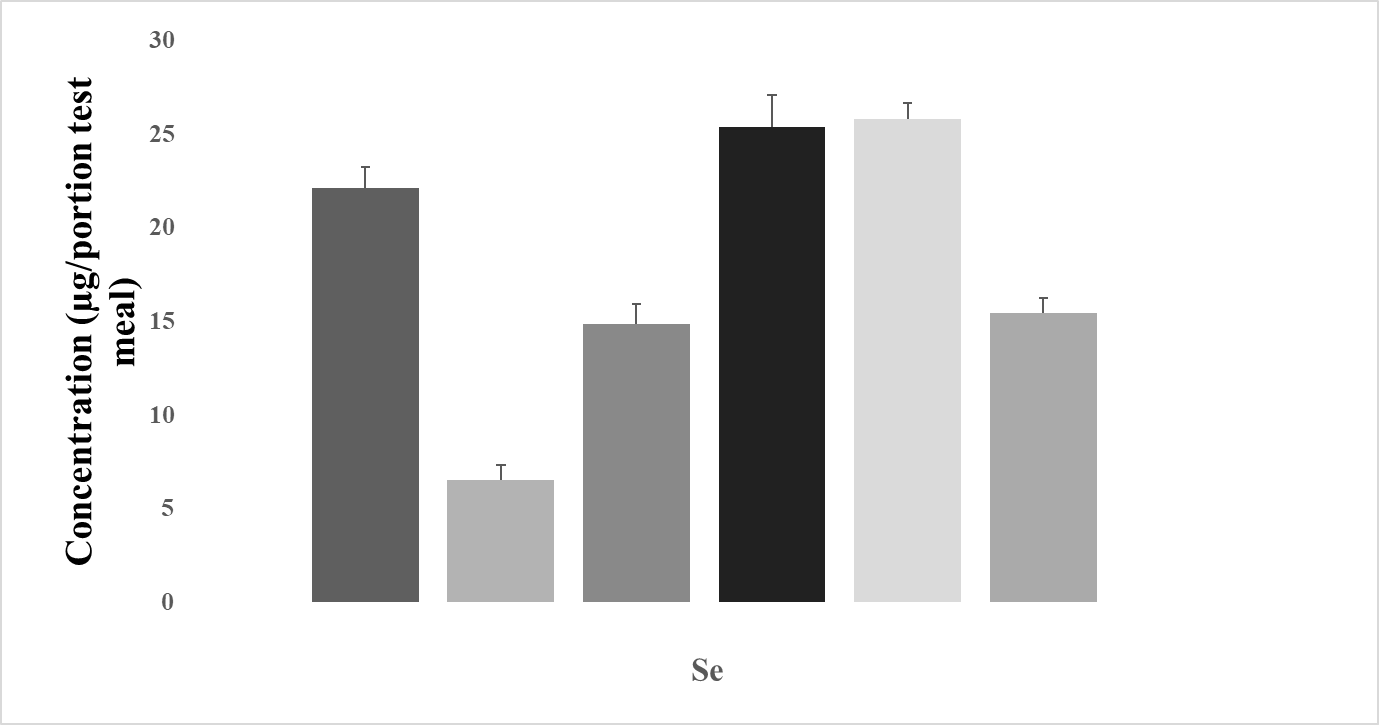
**

**C**

**Fig S 2 A, B, C** The micronutrient mineral composition of the study test meals, sodium (Na), magnesium (Mg), phosphorus (P), potassium (K), calcium (Ca) (A) in g/portion test meal, manganese (Mn), iron (Fe), cupper (Cu), zinc (Zn) (B) in mg/portion test meal, and selenium (Se) (C) in µg/portion test meal.

Table S2. A) Flavanols content of the test meals

|  | **green pea** | | **buckwheat** | | **hemp** | | **lupin** | | **fava bean** | | **wheat** | |
| --- | --- | --- | --- | --- | --- | --- | --- | --- | --- | --- | --- | --- |
|  | **free** | **bound** | **free** | **bound** | **free** | **bound** | **free** | **bound** | **free** | **bound** | **free** | **bound** |
| **catechin** | n/d | n/d | 10.31 ± 0.95 | n/d | 0.31 ± 0.03 | n/d | n/d | n/d | 0.66 ± 0.03 | n/d | n/d | n/d |
| **epicatechin** | n/d | n/d | 7.77 ± 0.56 | n/d | 0.10 ± 0.02 | n/d | n/d | 0.10 ± 0.00 | 0.41 ± 0.06 | n/d | n/d | n/d |

Data is given as mean ± standard deviation and is expressed as mg per serving DW of test bread. n/d = not detected (i.e. below the detection level). Gallocatechin, epigallocatechin, epigallocatechin gallate were not detected in any of the test meals

**Table S2.B) Anthocyanin content of the test breads.**

|  | **green pea** | **buckwheat** | **hemp** | **lupin** | **fava bean** | **wheat** |
| --- | --- | --- | --- | --- | --- | --- |
| **pelargonidin** | n/d | 33.82 ± 8.77 | n/d | n/d | n/d | n/d |
| **cyanidin** | n/d | 16.39 ± 2.33 | 3.73 ± 0.28 | n/d | 3.25 ± 0.20 | n/d |
| **delphinidin** | n/d | n/d | n/d | n/d | 4.06 ± 0.39 | n/d |

Data is given as mean ± standard deviation and is expressed as mg per serving DW of test bread. n/d = not detected (i.e. below the detection level). Petunidin, peonidin, malvinidin were not detected in any of the test meals

Table S2. C) Flavonoids content of the test meals

Other flavonoids (1); coumarins (2); isoflavonoids (3).

|  | **green pea** | **buckwheat** | **hemp** | **lupin** | **fava bean** | **wheat** |
| --- | --- | --- | --- | --- | --- | --- |
| **1** |  |  |  |  |  |  |
| **tangeretin** | 0.00 ± 0.00 | 0.00 ± 0.00 | 0.00 ± 0.00 | 0.00 ± 0.00 | 0.00 ± 0.00 | 0.00 ± 0.00 |
| **coumesterol** | n/d | 0.00 ± 0.00 | n/d | n/d | n/d | n/d |
| **luteolinidin** | n/d | 0.02 ± 0.00 | n/d | n/d | n/d | n/d |
| **luteolin** | n/d | 0.09 ± 0.01 | 0.06 ± 0.04 | 0.03 ± 0.00 | 0.02 ± 0.00 | 0.00 ± 0.00 |
| **glycitein** | 0.01 ± 0.00 | n/d | n/d | n/d | n/d | n/d |
| **isoliquiritigenin** | 0.00 ± 0.00 | 0.00 ± 0.00 | n/d | n/d | 0.00 ± 0.00 | n/d |
| **phloretin** | n/d | 0.00 ± 0.00 | n/d | n/d | n/d | n/d |
| **eriocitrin** | n/d | 0.00 ± 0.00 | n/d | n/d | n/d | n/d |
| **naringenin** | 0.01 ± 0.00 | 0.05 ± 0.01 | 0.00 ± 0.00 | 0.01 ± 0.00 | 0.01 ± 0.00 | 0.00 ± 0.00 |
| **naringin** | 0.00 ± 0.00 | 0.00 ± 0.00 | 0.00 ± 0.00 | 0.00 ± 0.00 | n/d | 0.00 ± 0.00 |
| **hesperitin** | 0.00 ± 0.00 | 0.00 ± 0.00 | 0.00 ± 0.00 | 0.00 ± 0.00 | 0.00 ± 0.00 | 0.00 ± 0.00 |
| **hesperidin** | 0.00 ± 0.00 | 0.01 ± 0.00 | 0.00 ± 0.00 | 0.00 ± 0.00 | 0.00 ± 0.00 | 0.00 ± 0.00 |
| **kaempferol** | 0.23 ± 0.02 | 0.08 ± 0.01 | 0.02 ± 0.02 | 0.02 ± 0.00 | 0.41 ± 0.05 | 0.01 ± 0.00 |
| **morin** | n/d | n/d | 0.01 ± 0.01 | 0.00 ± 0.00 | 0.00 ± 0.00 | n/d |
| **quercetin** | 0.08 ± 0.01 | 5.33 ± 0.23 | 0.08 ± 0.06 | 0.02 ± 0.00 | 0.51 ± 0.06 | 0.02 ± 0.01 |
| **quercetin-3-glucoside** | 0.01 ± 0.00 | 0.06 ± 0.00 | 0.00 ± 0.00 | 0.00 ± 0.00 | 0.02 ± 0.00 | 0.00 ± 0.00 |
| **quercitrin** | 0.00 ± 0.00 | 0.00 ± 0.00 | n/d | n/d | 0.00 ± 0.00 | n/d |
| **taxifolin** | 0.04 ±0.00 | 0.02 ± 0.00 | 0.01 ± 0.01 | n/d | 0.02 ± 0.00 | n/d |
| **myricetin** | 0.02 ± 0.01 | 0.01 ± 0.00 | 0.00 ± 0.00 | 0.00 ± 0.00 | 0.14 ± 0.02 | 0.00 ± 0.00 |
| **poncirin** | n/d | 0.00 ± 0.00 | n/d | n/d | n/d | n/d |
| **galangin** | 0.00 ± 0.00 | 0.00 ± 0.00 | n/d | n/d | n/d | n/d |
| **fisetin** | 0.00 ± 0.00 | n/d | n/d | n/d | n/d | n/d |
| **apigenin** | 0.00 ± 0.00 | 0.01 ± 0.00 | 0.02 ± 0.01 | 0.04 ± 0.00 | 0.01 ± 0.00 | 0.00 ± 0.00 |
| **tyrosol** | 0.34 ± 0.03 | 0.34 ± 0.02 | 0.38 ± 0.05 | 1.11 ± 0.14 | 0.30 ± 0.02 | 0.10 ± 0.01 |
| **hydroxytyrosol** | 0.00 ± 0.00 | 0.00 ± 0.00 | 0.02 ± 0.01 | 0.00 ± 0.00 | 0.01 ± 0.01 | 0.00 ± 0.00 |
| **isorhamnetin** | 0.10 ± 0.01 | 0.19 ± 0.01 | 0.13 ± 0.01 | 0.10 ± 0.01 | 0.25 ± 0.03 | 0.08 ± 0.02 |
| **2** |  |  |  |  |  |  |
| **coumarin** | 0.01 ± 0.01 | 0.02 ± 0.00 | 0.14 ± 0.10 | n/d | n/d | n/d |
| **psoralen** | n/d | 0.00 ± 0.00 | 0.00 ± 0.00 | n/d | n/d | n/d |
| **8-methylpsoralen** | n/d | n/d | 0.00 ± 0.00 | n/d | n/d | n/d |
| **umbelliferone** | 0.00 ± 0.00 | 0.02 ± 0.00 | 0.00 ± 0.00 | n/d | 0.00 ± 0.00 | n/d |
| **7,8-dihydroxy-6-methylcoumarin** | 0.00 ± 0.00 | n/d | n/d | n/d | 0.00 ± 0.00 | n/d |
| **scopoletin** | 0.00 ± 0.01 | 0.01 ± 0.00 | 0.01 ± 0.00 | 0.00 ± 0.00 | 0.01 ± 0.00 | 0.00 ± 0.00 |
| **3** |  |  |  |  |  |  |
| **genistein** | 0.00 ± 0.00 | 0.00 ± 0.00 | 0.00 ± 0.00 | 0.08 ± 0.01 | n/d | 0.00 ± 0.00 |
| **biochanin a** | 0.00 ± 0.00 | 0.00 ± 0.00 | 0.00 ± 0.00 | 0.00 ± 0.00 | 0.00 ± 0.00 | 0.00 ± 0.00 |
| **daidzein** | 0.00 ± 0.00 | 0.00 ± 0.00 | 0.00 ± 0.00 | 0.00 ± 0.00 | 0.00 ± 0.00 | 0.00 ± 0.00 |
| **formononetin** | 0.02 ± 0.01 | 0.04 ± 0.05 | 0.01 ± 0.01 | 0.00 ± 0.00 | 0.01 ± 0.00 | 0.00 ± 0.00 |

Data is given as mean ± standard deviation and is expressed as mg per serving DW of test bread. n/d = not detected (i.e. below the detection level). The following compounds were not detected in any of the samples: bergapten, neohesperidin, didymin, phloridzin, equol, neoeriocitrin, gossypin, resveratrol.

**Table S2. D) Content of phenolic acids and derivatives of the test meals**

Benzoic acids (1); benzaldehydes (2); cinnamic acids (3); benzenes and acetophenones (4); phenylacetic, phenylpyruvic and phenyllactic acids (5); phenolic dimers and lignans (6); others (7).

|  | **green pea** | | **buckwheat** | | **hemp** | | **lupin** | | **fava bean** | | **wheat** | |
| --- | --- | --- | --- | --- | --- | --- | --- | --- | --- | --- | --- | --- |
|  | **free** | **bound** | **free** | **bound** | **free** | **bound** | **free** | **bound** | **free** | **bound** | **free** | **bound** |
| **1** |  |  |  |  |  |  |  |  |  |  |  |  |
| **benzoic acid** | 0.50 ±  0.10 | 1.11 ±  0.14 | 0.69 ±  0.04 | 2.31 ±  0.10 | 0.56 ±  0.05 | 1.24 ±  0.14 | 0.44 ±  0.04 | 1.08 ±  0.10 | 0.36 ±  0.03 | 1.49 ±  0.17 | 0.22 ±  0.03 | 0.65 ±  0.10 |
| **salicylic acid** | 0.09 ±  0.01 | 0.07 ±  0.03 | 0.24 ±  0.05 | 0.77 ±  0.04 | 1.14 ±  0.03 | 0.24 ±  0.01 | n/d | n/d | 0.13 ±  0.02 | 0.14 ±  0.01 | 0.04 ±  0.01 | 0.00 ±  0.00 |
| ***p-*hydroxy-benzoic acid** | n/d | 0.36 ±  0.32 | n/d | 1.21 ±  0.05 | n/d | 0.26 ±  0.03 | n/d | 0.17 ±  0.02 | n/d | 0.33 ±  0.04 | n/d | n/d |
| **2,3-dihydroxy-benzoic acid** | n/d | 0.00 ±  0.01 | n/d | 0.40 ±  0.02 | n/d | n/d | n/d | n/d | n/d | 0.01 ±  0.01 | n/d | 0.01 ±  0.00 |
| **gentisic acid** | n/d | 0.04 ±  0.04 | n/d | 0.67 ±  0.03 | 0.03 ±  0.00 | 1.17 ±  0.08 | n/d | n/d | n/d | 0.23 ±  0.02 | n/d | n/d |
| **2,6-dihydroxy-benzoic acid** | n/d | n/d | n/d | n/d | n/d | n/d | n/d | n/d | 0.02 ±  0.00 | n/d | n/d | n/d |
| **protocatechuic acid** | 0.09 ±  0.02 | 0.42 ±  0.35 | 0.33 ±  0.04 | 0.65 ±  0.04 | 0.23 ±  0.01 | 0.65 ±  0.03 | n/d | n/d | 0.03 ±  0.01 | 0.07 ±  0.00 | 0.01 ±  0.00 | 0.01 ±  0.00 |
| **gallic acid** | n/d | n/d | 0.34 ±  0.05 | 0.60 ±  0.08 | n/d | n/d | n/d | n/d | n/d | n/d | n/d | n/d |
| **vanillic acid** | 0.10 ±  0.01 | 0.43 ±  0.17 | 0.13 ±  0.01 | 0.66 ±  0.01 | 0.21 ±  0.01 | 0.77 ±  0.04 | 0.08 ±  0.01 | 0.49 ±  0.04 | 0.13 ±  0.01 | 0.55 ±  0.02 | 0.04 ±  0.00 | 0.18 ±  0.01 |
| **syringic acid** | 0.03 ±  0.00 | 0.14 ±  0.02 | 0.04 ±  0.01 | 0.20 ±  0.01 | 0.06 ±  0.02 | 0.34 ±  0.03 | 0.01 ±  0.00 | 0.13 ±  0.01 | 0.03 ±  0.01 | 0.27 ±  0.01 | 0.02 ±  0.00 | 0.13 ±  0.01 |
| **2** |  |  |  |  |  |  |  |  |  |  |  |  |
| ***p-*hydroxy-benzaldehyde** | 0.16 ±  0.02 | n/d | 0.18 ±  0.06 | 0.84 ±  0.08 | 0.38 ±  0.09 | 0.60 ±  0.08 | 0.13 ±  0.03 | 0.15 ±  0.02 | n/d | n/d | 0.06 ±  0.01 | n/d |
| **protocatachaldehyde** | n/d | n/d | 0.14 ±  0.00 | 0.77 ±  0.08 | 0.25 ±  0.01 | 1.26 ±  0.04 | n/d | n/d | 0.02 ±  0.00 | 0.10 ±  0.04 | n/d | n/d |
| **3,4,5-trihydroxy-benzaldehyde** | n/d | n/d | n/d | n/d | n/d | n/d | n/d | n/d | n/d | 0.03 ±  0.03 | n/d | n/d |
| **vanillin** | 0.03 ±  0.00 | 0.05 ±  0.02 | 0.04 ±  0.00 | 0.12 ±  0.01 | 0.12 ±  0.00 | 0.57 ±  0.03 | 0.03 ±  0.00 | 0.14 ±  0.01 | 0.09 ±  0.00 | 0.26 ±  0.05 | 0.02 ±  0.00 | 0.05 ±  0.01 |
| **syringin** | 0.01 ±  0.00 | 0.01 ±  0.01 | 0.01 ±  0.00 | 0.02 ±  0.00 | 0.04 ±  0.00 | 0.23 ±  0.02 | 0.00 ±  0.00 | 0.01 ±  0.00 | 0.01 ±  0.00 | 0.01 ±  0.01 | 0.00 ±  0.00 | 0.00 ±  0.00 |
| **3** |  |  |  |  |  |  |  |  |  |  |  |  |
| **cinnamic acid** | 0.14 ±  0.04 | 0.24 ±  0.02 | 0.21 ±  0.04 | 0.37 ±  0.06 | 0.16 ±  0.01 | 0.24 ±  0.02 | 0.12 ±  0.03 | 0.19 ±  0.00 | 0.10 ±  0.00 | 0.28 ±  0.04 | 0.07 ±  0.01 | 0.13 ±  0.02 |
| ***p-*coumaric acid** | 0.07 ±  0.13 | n/d | 0.44 ±  0.13 | 0.64 ±  0.12 | 0.52 ±  0.04 | 3.50 ±  0.19 | n/d | n/d | 0.39 ±  0.03 | 2.07 ±  0.16 | n/d | 0.14 ±  0.03 |
| **caffeic acid** | 0.01 ±  0.00 | 0.07 ±  0.03 | 0.16 ±  0.03 | 1.16 ±  0.18 | 0.05 ±  0.00 | 0.13 ±  0.01 | n/d | 0.03 ±  0.00 | 0.03 ±  0.00 | 0.19 ±  0.01 | n/d | 0.06 ±  0.00 |
| **ferulic acid** | 0.34 ±  0.01 | 4.70 ±  2.13 | 0.21 ±  0.03 | 3.94 ±  0.31 | 0.30 ±  0.01 | 3.87 ±  0.19 | 0.15 ±  0.01 | 2.60 ±  0.31 | 0.39 ±  0.03 | 18.72 ±  0.71 | 0.31 ±  0.02 | 3.57 ±  0.25 |
| **sinapic acid** | 0.12 ±  0.01 | 1.51 ±  0.66 | 0.21 ±  0.01 | 1.39 ±  0.10 | 0.06 ±  0.01 | 0.90 ±  0.08 | 0.03 ±  0.01 | 0.16 ±  0.04 | 0.12 ±  0.01 | 2.00 ±  0.04 | 0.08 ±  0.01 | 0.66 ±  0.05 |
| **3,4-dimethoxy-cinnamic acid** | n/d | n/d | n/d | 0.02 ±  0.02 | n/d | n/d | n/d | n/d | 0.02 ±  0.00 | 0.05 ±  0.01 | n/d | n/d |
| **3,4,5-trimethoxy-cinnamic acid** | n/d | n/d | 0.01 ±  0.00 | 0.06 ±  0.00 | n/d | n/d | n/d | n/d | n/d | 0.00 ±  0.00 | n/d | n/d |
| **4-hydroxy-3-methoxy-cinnamaldehyde** | 0.02 ±  0.04 | n/d | 0.17 ±  0.15 | n/d | 0.91 ±  0.07 | 0.13 ±  0.01 | n/d | n/d | 0.07 ±  0.01 | n/d | 0.05 ±  0.00 | n/d |
| **4-hydroxy-3-methoxyphenylpropionic acid** | n/d | n/d | n/d | n/d | n/d | 0.04 ±  0.00 | n/d | 0.04 ±  0.01 | n/d | 0.03 ±  0.01 | n/d | 0.01 ±  0.00 |
| **4** |  |  |  |  |  |  |  |  |  |  |  |  |
| **phenol** | n/d | 0.32 ±  0.55 | n/d | 1.15 ±  0.19 | n/d | 4.49 ±  0.64 | n/d | 0.80 ±  0.14 | n/d | 1.04 ±  0.11 | n/d | n/d |
| **1,2-hydroxybenzene** | n/d | n/d | n/d | n/d | n/d | n/d | n/d | n/d | n/d | n/d | 0.04 ±  0.00 | n/d |
| **4-hydroxy-acetophenone** | 0.00 ±  0.00 | 0.01 ±  0.01 | n/d | 0.02 ±  0.00 | 0.01 ±  0.00 | 0.02 ±  0.00 | 0.01 ±  0.00 | 0.02 ±  0.00 | 0.00 ±  0.00 | 0.02 ±  0.00 | 0.00 ±  0.00 | 0.01 ±  0.00 |
| **4-hydroxy-3-methoxy-acetophenone** | n/d | 0.03 ±  0.03 | n/d | 0.07 ±  0.01 | 0.01 ±  0.00 | 0.09 ±  0.01 | n/d | 0.07 ±  0.00 | 0.01 ±  0.00 | 0.09 ±  0.01 | 0.01 ±  0.00 | 0.04 ±  0.00 |
| **4-hydroxy-3,5-dimethoxy-acetophenone** | 0.00 ±  0.00 | 0.08 ±  0.04 | n/d | 0.06 ±  0.01 | n/d | 0.06 ±  0.01 | n/d | 0.05 ±  0.01 | n/d | 0.33 ±  0.01 | 0.00 ±  0.00 | 0.07 ±  0.01 |
| **5** |  |  |  |  |  |  |  |  |  |  |  |  |
| **phenylacetic acid** | 0.81 ±  0.09 | 0.68 ±  0.07 | 1.02 ±  0.09 | 0.78 ±  0.07 | 0.68 ±  0.03 | 0.53 ±  0.05 | 0.50 ±  0.03 | 0.55 ±  0.05 | 0.42 ±  0.01 | 0.67 ±  0.10 | 0.36 ±  0.01 | 0.28 ±  0.02 |
| **3-hydroxy-phenylacetic acid** | n/d | n/d | n/d | 3.45 ±  0.31 | n/d | n/d | n/d | n/d | n/d | 0.29 ±  0.02 | n/d | n/d |
| **phenyl-**  **lactic acid** | 0.16 ±  0.01 | 0.02 ±  0.02 | 0.11 ±  0.01 | 0.43 ±  0.01 | 0.05 ±  0.01 | 0.02 ±  0.00 | 0.10 ±  0.01 | 0.02 ±  0.00 | 0.15 ±  0.01 | 0.06 ±  0.01 | 0.03 ±  0.00 | 0.01 ±  0.00 |
| **phenyl-**  **pyruvic acid** | 0.24 ±  0.02 | 0.01 ±  0.02 | 0.11 ±  0.01 | 0.03 ±  0.02 | 0.10 ±  0.00 | 0.07 ±  0.00 | 0.25 ±  0.03 | 0.07 ±  0.01 | 0.19 ±  0.03 | 0.14 ±  0.04 | 0.03 ±  0.01 | 0.02 ±  0.00 |
| **4-hydroxy-phenyl-**  **pyruvic acid** | 2.15 ±  0.55 | 1.60 ±  0.84 | 1.95 ±  0.24 | 2.42 ±  0.60 | 1.87 ±  0.36 | 2.21 ±  0.63 | 2.72 ±  0.50 | 1.60 ±  0.15 | 2.91 ±  0.46 | 1.85 ±  0.54 | 0.59 ±  0.07 | 0.84 ±  0.06 |
| **6** |  |  |  |  |  |  |  |  |  |  |  |  |
| **ferulic dimer (5-5 linked)** | n/d | 0.63 ±  0.37 | n/d | 0.56 ±  0.05 | n/d | 0.54 ±  0.06 | n/d | 0.73 ±  0.07 | n/d | 5.66 ±  0.14 | n/d | 0.58 ±  0.06 |
| **secoisoresinol** | n/d | 0.03 ±  0.03 | 0.01 ±  0.00 | n/d | 0.01 ±  0.00 | 0.18 ±  0.02 | n/d | 0.06 ±  0.01 | n/d | n/d | n/d | 0.04 ±  0.00 |
| **matairesinol** | n/d | 0.02 ±  0.01 | n/d | 0.02 ±  0.01 | n/d | 0.01 ±  0.01 | n/d | 0.02 ±  0.00 | n/d | 0.04 ±  0.01 | n/d | 0.01 ±  0.00 |
| **syringoresinol** | n/d | n/d | 0.48 ±  0.03 | n/d | 0.25 ±  0.23 | 3.79 ±  0.10 | n/d | n/d | n/d | n/d | n/d | n/d |
| **pinoresinol** | n/d | n/d | 0.04 ±  0.00 | n/d | 0.08 ±  0.01 | 0.17 ±  0.02 | n/d | n/d | 0.02 ±  0.02 | n/d | n/d | n/d |
| **lariciresinol** | n/d | n/d | 0.02 ±  0.04 | n/d | n/d | n/d | 0.05 ±  0.00 | n/d | n/d | n/d | n/d | n/d |
| **7** |  |  |  |  |  |  |  |  |  |  |  |  |
| **ethylferulate** | 0.01 ±  0.00 | n/d | 0.01 ±  0.00 | n/d | 0.01 ±  0.00 | n/d | n/d | n/d | 0.01 ±  0.00 | n/d | n/d | n/d |
| **coniferyl alcohol** | 0.05 ±  0.01 | n/d | 0.08 ±  0.02 | n/d | 0.09 ±  0.00 | n/d | 0.03 ±  0.02 | n/d | 0.14 ±  0.02 | n/d | 0.05 ±  0.01 | n/d |

Data is given as mean ± standard deviation and is expressed as mg per serving DW of test meal. n/d = not detected (i.e. below the detection level).

The following compounds were not detected in any of the samples reported in Table S2 A) to D).: 3-methoxybenzaldehyde, 3,4-dimethoxybenzaldehyde, 3,4,5-trimethoxybenzaldehyde, 2-hydroxycinnamyl alcohol, 3-methoxycinnamic acid, 4-methoxycinnamic acid, 2-hydroxyphenylpropionic acid, 3-hydroxyphenylpropionic acid, 4-hydroxyphenylpropionic acid, 3,4 dihydroxyphenylpropionic acid, hydrogenated ferulic dimer H5-5, ferulic dimer (8-8 linked), ferulic dimer (8-5 linked), resveratrol, gossypin, OH-mata, 4-hydroxyphenyllactic acid, chlorogenic acid, *o*-hydroxyhippuric acid, *m*-hydroxybenzoic acid, 2,4-dihydroxybenzoic acid, 3,5-dihydroxybenzoic acid, *o*-anisic acid, *m*-anisic acid, *p*-anisic acid, 3,4-dimethoxybenzoic acid, isovanillin, *o*-coumaric acid, *m*-coumaric acid, 3-methoxyphenylpropionic acid, 1,3-hydroxybenzene, 1,2,3-trihydroxybenzene, 3,4-dimethoxyacetophenone, 3,4,5-trimethoxyacetophenone, 4-hydroxyphenylacetic acid, 3,4-dihydroxyphenylacetic acid, 4-hydroxy-3-methoxyphenylacetic acid, 4-methoxyphenylacetic acid, mandelic acid, 3-hydroxymandelic acid, 4-hydroxymandelic acid, 3,4-dihydroxymandelic acid, 4-hydroxy-3-methoxymandelic acid, p-cresol, 4-ethylphenol, hydroxytyrosol, ellagic acid, e-diol, e-lac, 4-methylcatechol were not detected in any of the test meals

**Table S3.** The average (n=10) plasma concentration for each of the amino acid (in nmoles/ g plasma) after the consumption of test meals at time 30, 60, 90, 120, 150, 180 and 300 minutes andAUC, **iAUC** with average SEM. Where, histidine (His), serine (Ser), arginine (Arg), glycine (Gly), aspartic acid (Asp), glutamic acid (Glu), threonine (Thr), alanine (Ala), proline (Pro), lysine (Lys), tyrosine (Tyr), valine (Val), Isoleucine (ILeu), leucine (Leu), phenylalanine (Phe), methionine (Met) and cysteine (Cys). p values for ANOVA overall analysis, analysis for each test meal vs meat meal and meat meals vs plant-based meals and plant vs plant meals.

| **AA/time (min)** | **Buckwheat** | **p meat vs buckwheat** | **Fava** | **p meat vs Fava** | **Green pea** | **p meat vs Green Pea** | **Hemp** | **p meat vs Hemp** | **Lupin** | **p meat vs Lupin** | **Meat** | **p overall** | **p meat vs plants** | **p plants vs plants** |
| --- | --- | --- | --- | --- | --- | --- | --- | --- | --- | --- | --- | --- | --- | --- |
|  | **nmoles/ g plasma after the consumption of test meals** | | | |  |  |  |  |  |  |  |  |  |  |
| Ala 30 | 385.74 | 1.000 | 383.85 | 1.000 | 390.27 | 0.988 | 383.77 | 1.000 | 387.89 | 0.996 | 384.01 | 0.974 | 0.733 | 0.950 |
| Ala 60 | 405.49 | 0.959 | 417.21 | 1.000 | 436.00 | 0.743 | 423.41 | 0.992 | 423.92 | 0.987 | 416.55 | 0.355 | 0.603 | 0.265 |
| Ala 90 | 420.80 | 0.986 | 426.93 | 1.000 | 455.16 | 0.640 | 436.07 | 0.998 | 432.94 | 1.000 | 430.54 | 0.356 | 0.768 | 0.251 |
| Ala 120 | 409.25 | 0.547 | 410.24 | 0.705 | 438.38 | 1.000 | 432.25 | 1.000 | 422.89 | 0.943 | 434.73 | 0.292 | 0.354 | 0.259 |
| Ala 150 | 403.66 | 0.800 | 393.93 | 0.552 | 426.96 | 1.000 | 424.16 | 1.000 | 405.15 | 0.752 | 423.33 | 0.226 | 0.333 | 0.198 |
| Ala 180 | 397.53 | 0.756 | 392.32 | 0.669 | 404.15 | 0.954 | 407.27 | 0.967 | 372.71 | 0.016 | 418.36 | 0.038 | 0.020 | 0.145 |
| Ala 300 | 350.38 | 1.000 | 363.51 | 0.978 | 357.53 | 0.997 | 329.67 | 0.905 | 318.70 | 0.495 | 347.91 | 0.174 | 0.300 | 0.155 |
| **AUC** | **116981.81** | **0.933** | **117553.82** | **0.982** | **121585.48** | **0.999** | **119041.71** | **0.999** | **114610.01** | **0.431** | **######** | **0.335** | **0.228** | **0.369** |
| **iAUC** | **10570.81** | **0.738** | **12208.99** | **0.973** | **15307.79** | **1.000** | **12756.33** | **0.974** | **10684.80** | **0.632** | **14857.22** | **0.461** | **0.186** | **0.577** |
| Gly30 | 226.22 | 1.000 | 227.64 | 0.989 | 226.93 | 0.997 | 226.01 | 1.000 | 230.09 | 0.628 | 225.21 | 0.723 | 0.337 | 0.753 |
| Gly60 | 226.77 | 0.997 | 233.92 | 0.953 | 237.52 | 0.624 | 231.30 | 0.996 | 239.56 | 0.189 | 229.09 | 0.077 | 0.159 | 0.088 |
| Gly90 | 227.17 | 0.985 | 237.95 | 0.872 | 239.39 | 0.728 | 235.87 | 0.923 | 241.41 | 0.301 | 230.90 | 0.100 | 0.152 | 0.119 |
| Gly120 | 231.36 | 0.990 | 235.38 | 1.000 | 238.41 | 0.996 | 237.92 | 0.996 | 240.69 | 0.910 | 235.12 | 0.703 | 0.564 | 0.622 |
| Gly150 | 231.01 | 0.999 | 228.37 | 0.987 | 234.24 | 1.000 | 234.53 | 1.000 | 234.50 | 1.000 | 233.53 | 0.960 | 0.991 | 0.906 |
| Gly180 | 232.19 | 0.999 | 232.01 | 0.999 | 229.04 | 0.974 | 229.67 | 0.967 | 225.12 | 0.655 | 234.95 | 0.747 | 0.234 | 0.871 |
| Gly300 | 229.55 | 0.348 | 230.99 | 0.392 | 224.09 | 0.759 | 210.98 | 1.000 | 213.16 | 0.999 | 210.38 | 0.190 | 0.318 | 0.167 |
| **AUC** | **68856.51** | **0.999** | **69534.51** | **0.980** | **69324.06** | **0.989** | **68352.42** | **1.000** | **68622.21** | **1.000** | **68235.24** | **0.979** | **0.704** | **0.961** |
| **iAUC** | **3130.23** | **1.000** | **3588.94** | **1.000** | **4136.53** | **0.996** | **3180.16** | **1.000** | **3553.02** | **1.000** | **3476.06** | **0.977** | **0.957** | **0.941** |
| Val30 | 224.80 | 0.826 | 233.31 | 0.606 | 232.47 | 0.710 | 230.85 | 0.893 | 231.73 | 0.679 | 228.22 | 0.036 | 0.258 | 0.030 |
| Val60 | 226.65 | 0.045 | 242.84 | 0.999 | 247.09 | 0.787 | 239.70 | 1.000 | 245.69 | 0.802 | 240.71 | <0.001 | 0.952 | <0.001 |
| Val90 | 224.56 | 0.002 | 246.46 | 0.997 | 252.24 | 0.553 | 246.64 | 0.986 | 251.80 | 0.363 | 243.92 | <0.001 | 0.559 | <0.001 |
| Val120 | 223.85 | 0.003 | 246.32 | 1.000 | 254.00 | 0.922 | 253.85 | 0.858 | 258.79 | 0.269 | 247.78 | <0.001 | 0.442 | <0.001 |
| Val150 | 219.23 | <0.001 | 242.05 | 0.915 | 255.41 | 0.960 | 257.52 | 0.749 | 256.92 | 0.775 | 249.56 | <0.001 | 0.792 | <0.001 |
| Val180 | 218.88 | <0.001 | 241.90 | 0.520 | 251.52 | 0.974 | 254.42 | 0.996 | 250.75 | 0.896 | 258.11 | <0.001 | 0.065 | <0.001 |
| Val300 | 209.04 | <0.001 | 226.09 | 0.008 | 239.22 | 0.200 | 233.51 | 0.011 | 227.41 | <0.001 | 258.29 | <0.001 | <0.001 | 0.004 |
| **AUC** | **65773.21** | **<0.001** | **71275.93** | **0.346** | **73693.62** | **0.996** | **73226.91** | **0.924** | **73032.60** | **0.842** | **74389.90** | **<0.001** | **0.018** | **<0.001** |
| **iAUC** | **2244.71** | **<0.001** | **6730.27** | **0.619** | **8303.53** | **0.991** | **7986.31** | **0.924** | **8626.46** | **0.997** | **9147.50** | **<0.001** | **0.073** | **<0.001** |
| Leu30 | 125.55 | 0.835 | 136.24 | 0.267 | 134.14 | 0.535 | 130.03 | 0.998 | 135.81 | 0.077 | 129.02 | 0.001 | 0.138 | <0.001 |
| Leu60 | 125.27 | 0.010 | 144.51 | 1.000 | 148.57 | 0.919 | 136.96 | 0.661 | 152.58 | 0.270 | 143.42 | <0.001 | 0.884 | <0.001 |
| Leu90 | 120.83 | <0.001 | 148.02 | 1.000 | 151.84 | 0.939 | 141.09 | 0.786 | 161.49 | 0.020 | 146.86 | <0.001 | 0.797 | <0.001 |
| Leu120 | 116.71 | <0.001 | 146.91 | 0.998 | 151.40 | 1.000 | 144.79 | 0.924 | 170.07 | 0.006 | 150.02 | <0.001 | 0.632 | <0.001 |
| Leu150 | 111.00 | <0.001 | 143.34 | 0.878 | 150.94 | 1.000 | 144.38 | 0.784 | 168.62 | 0.059 | 151.94 | <0.001 | 0.805 | <0.001 |
| Leu180 | 109.69 | <0.001 | 144.58 | 0.838 | 146.43 | 0.877 | 137.57 | 0.124 | 160.69 | 0.985 | 156.03 | <0.001 | 0.056 | <0.001 |
| Leu300 | 98.86 | <0.001 | 127.22 | 0.010 | 128.52 | 0.006 | 115.88 | <0.001 | 136.07 | 0.018 | 155.39 | <0.001 | <0.001 | <0.001 |
| **AUC** | **33888.10** | **<0.001** | **41789.24** | **0.464** | **42548.86** | **0.713** | **39999.46** | **0.002** | **45611.75** | **0.857** | **44400.42** | **<0.001** | **0.005** | **<0.001** |
| **iAUC** | **1148.29** | **<0.001** | **6993.52** | **0.603** | **7552.54** | **0.752** | **5476.32** | **0.013** | **10987.05** | **0.711** | **9408.52** | **<0.001** | **0.035** | **<0.001** |
| Ileu30 | 66.03 | 0.974 | 71.16 | 0.460 | 70.37 | 0.496 | 69.45 | 0.652 | 72.23 | 0.011 | 67.28 | 0.001 | 0.020 | 0.003 |
| Ileu60 | 66.77 | 0.019 | 77.08 | 1.000 | 80.62 | 0.877 | 75.23 | 0.980 | 83.68 | 0.121 | 77.08 | <0.001 | 0.666 | <0.001 |
| Ileu90 | 65.23 | <0.001 | 80.26 | 1.000 | 83.83 | 0.827 | 79.29 | 1.000 | 90.04 | 0.004 | 79.84 | <0.001 | 0.292 | <0.001 |
| Ileu120 | 64.02 | <0.001 | 79.40 | 0.977 | 84.84 | 0.997 | 83.25 | 1.000 | 96.50 | 0.002 | 82.97 | <0.001 | 0.210 | <0.001 |
| Ileu150 | 61.27 | <0.001 | 77.07 | 0.670 | 85.78 | 1.000 | 84.54 | 1.000 | 96.96 | 0.016 | 85.04 | <0.001 | 0.528 | <0.001 |
| Ileu180 | 61.01 | <0.001 | 78.34 | 0.589 | 84.22 | 0.933 | 81.80 | 0.551 | 93.17 | 0.969 | 89.61 | <0.001 | 0.153 | <0.001 |
| Ileu300 | 56.87 | <0.001 | 68.05 | 0.010 | 76.12 | 0.097 | 69.44 | <0.001 | 78.51 | 0.082 | 89.10 | <0.001 | <0.001 | <0.001 |
| **AUC** | **18581.44** | **<0.001** | **22394.26** | **0.217** | **23940.14** | **0.934** | **22979.71** | **0.127** | **25770.52** | **0.611** | **24721.89** | **<0.001** | **0.063** | **<0.001** |
| **iAUC** | **1215.38** | **<0.001** | **4467.29** | **0.278** | **5911.71** | **0.928** | **5012.79** | **0.161** | **7855.31** | **0.594** | **6744.38** | **<0.001** | **0.108** | **<0.001** |
| Pro30 | 178.28 | 0.956 | 184.72 | 0.238 | 181.95 | 0.539 | 180.97 | 0.518 | 180.30 | 0.630 | 175.05 | 0.283 | 0.056 | 0.623 |
| Pro60 | 187.13 | 0.243 | 199.85 | 1.000 | 202.89 | 0.998 | 205.92 | 0.884 | 206.67 | 0.812 | 200.19 | 0.021 | 0.626 | 0.012 |
| Pro90 | 191.82 | 0.044 | 210.82 | 1.000 | 218.54 | 0.864 | 216.58 | 0.914 | 221.10 | 0.463 | 210.90 | <0.001 | 0.539 | <0.001 |
| Pro120 | 196.81 | 0.015 | 214.96 | 0.984 | 220.61 | 1.000 | 224.54 | 0.977 | 226.41 | 0.898 | 220.09 | 0.001 | 0.903 | <0.001 |
| Pro150 | 193.84 | 0.002 | 213.36 | 0.918 | 222.61 | 1.000 | 225.85 | 0.944 | 227.35 | 0.846 | 220.61 | <0.001 | 0.873 | <0.001 |
| Pro180 | 194.37 | 0.006 | 213.90 | 0.847 | 214.72 | 0.858 | 223.83 | 1.000 | 218.88 | 0.966 | 224.60 | 0.008 | 0.128 | 0.008 |
| Pro300 | 176.69 | 0.200 | 198.69 | 0.988 | 195.88 | 1.000 | 185.13 | 0.802 | 185.82 | 0.831 | 193.55 | 0.040 | 0.136 | 0.047 |
| **AUC** | **55978.73** | **0.007** | **61012.52** | **0.999** | **61618.18** | **1.000** | **62017.68** | **1.000** | **61743.38** | **1.000** | **61593.84** | **0.002** | **0.452** | **<0.001** |
| **iAUC** | **8955.35** | **0.007** | **13946.19** | **0.999** | **14570.86** | **1.000** | **15016.62** | **1.000** | **14738.93** | **1.000** | **14597.22** | **0.002** | **0.443** | **<0.001** |
| Met30 | 32.32 | 0.963 | 31.09 | 0.409 | 31.71 | 0.698 | 33.82 | 0.939 | 31.91 | 0.683 | 33.08 | 0.071 | 0.467 | 0.048 |
| Met60 | 32.14 | 0.079 | 30.38 | 0.008 | 32.22 | 0.101 | 35.87 | 1.000 | 31.89 | 0.014 | 35.78 | <0.001 | 0.013 | 0.002 |
| Met90 | 31.37 | 0.021 | 29.21 | 0.001 | 30.87 | 0.010 | 36.48 | 1.000 | 30.71 | <0.001 | 36.22 | <0.001 | 0.002 | <0.001 |
| Met120 | 29.46 | 0.001 | 26.22 | <0.001 | 27.88 | <0.001 | 37.20 | 1.000 | 28.82 | <0.001 | 37.10 | <0.001 | <0.001 | <0.001 |
| Met150 | 28.02 | 0.001 | 24.06 | <0.001 | 25.99 | <0.001 | 36.46 | 1.000 | 26.12 | <0.001 | 36.81 | <0.001 | <0.001 | <0.001 |
| Met180 | 27.04 | <0.001 | 22.74 | <0.001 | 23.08 | <0.001 | 34.47 | 0.475 | 22.92 | <0.001 | 38.26 | <0.001 | <0.001 | <0.001 |
| Met300 | 22.53 | <0.001 | 18.41 | <0.001 | 18.42 | <0.001 | 27.12 | <0.001 | 18.05 | <0.001 | 35.61 | <0.001 | <0.001 | <0.001 |
| **AUC** | **8435.05** | **<0.001** | **7492.73** | **<0.001** | **7757.79** | **<0.001** | **10089.94** | **0.384** | **7740.47** | **<0.001** | **10831.30** | **<0.001** | **<0.001** | **<0.001** |
| **iAUC** | **220.21** | **0.003** | **89.70** | **0.004** | **279.34** | **0.005** | **1356.94** | **0.670** | **171.85** | **<0.001** | **1892.93** | **<0.001** | **<0.001** | **0.010** |
| Ser30 | 93.06 | 0.963 | 99.81 | 0.021 | 97.54 | 0.342 | 95.55 | 0.935 | 97.05 | 0.291 | 94.28 | 0.002 | 0.090 | 0.002 |
| Ser60 | 93.00 | 0.561 | 101.61 | 0.785 | 103.01 | 0.464 | 100.23 | 0.901 | 102.65 | 0.307 | 97.61 | 0.005 | 0.181 | 0.004 |
| Ser90 | 90.77 | 0.183 | 102.30 | 0.528 | 105.20 | 0.077 | 101.95 | 0.380 | 103.71 | 0.075 | 97.17 | <0.001 | 0.047 | <0.001 |
| Ser120 | 88.92 | 0.144 | 97.70 | 1.000 | 102.01 | 0.627 | 104.43 | 0.098 | 103.78 | 0.125 | 96.78 | <0.001 | 0.064 | <0.001 |
| Ser150 | 88.35 | 0.201 | 96.14 | 1.000 | 99.85 | 0.815 | 102.66 | 0.164 | 102.51 | 0.145 | 95.69 | <0.001 | 0.086 | <0.001 |
| Ser180 | 89.56 | 0.196 | 96.32 | 1.000 | 94.18 | 0.940 | 100.99 | 0.861 | 98.05 | 1.000 | 97.49 | 0.036 | 0.893 | 0.019 |
| Ser300 | 86.41 | 0.703 | 93.75 | 0.957 | 90.22 | 1.000 | 89.38 | 0.995 | 88.05 | 0.899 | 90.86 | 0.288 | 0.346 | 0.257 |
| **AUC** | **26871.52** | **0.107** | **29119.85** | **0.963** | **29053.10** | **0.974** | **29483.40** | **0.581** | **29266.48** | **0.775** | **28545.27** | **0.001** | **0.412** | **<0.001** |
| **iAUC** | **896.28** | **0.384** | **2532.31** | **0.959** | **2800.67** | **0.778** | **2971.48** | **0.431** | **2764.32** | **0.640** | **2005.31** | **0.005** | **0.218** | **0.004** |
| Thr30 | 118.97 | 0.955 | 124.01 | 0.930 | 124.29 | 0.871 | 121.04 | 1.000 | 125.87 | 0.317 | 121.21 | 0.070 | 0.328 | 0.054 |
| Thr60 | 117.24 | 0.343 | 125.84 | 0.999 | 131.35 | 0.469 | 123.91 | 1.000 | 130.70 | 0.350 | 124.50 | 0.002 | 0.452 | 0.001 |
| Thr90 | 115.49 | 0.264 | 125.83 | 0.999 | 131.25 | 0.561 | 123.68 | 1.000 | 130.24 | 0.505 | 124.22 | 0.003 | 0.557 | 0.002 |
| Thr120 | 110.91 | 0.023 | 120.34 | 0.949 | 127.04 | 0.990 | 124.34 | 1.000 | 129.59 | 0.688 | 124.42 | <0.001 | 0.968 | <0.001 |
| Thr150 | 108.84 | 0.030 | 116.40 | 0.758 | 124.32 | 1.000 | 121.86 | 0.999 | 126.19 | 0.973 | 123.26 | 0.005 | 0.634 | 0.002 |
| Thr180 | 107.46 | 0.006 | 115.78 | 0.441 | 118.94 | 0.737 | 118.07 | 0.461 | 120.20 | 0.723 | 126.24 | 0.023 | 0.018 | 0.090 |
| Thr300 | 100.91 | 0.001 | 112.42 | 0.376 | 112.61 | 0.324 | 103.42 | 0.002 | 109.33 | 0.026 | 124.42 | <0.001 | <0.001 | 0.130 |
| **AUC** | **32958.40** | **0.004** | **35496.56** | **0.736** | **36531.35** | **0.993** | **35265.73** | **0.368** | **36546.49** | **0.985** | **37153.38** | **0.004** | **0.046** | **0.008** |
| **iAUC** | **779.53** | **0.026** | **2101.96** | **0.656** | **3171.47** | **0.998** | **2088.04** | **0.401** | **3033.39** | **0.976** | **3582.35** | **0.031** | **0.064** | **0.054** |
| Phe30 | 59.11 | 0.981 | 62.56 | 0.071 | 62.18 | 0.076 | 60.07 | 0.618 | 61.00 | 0.183 | 58.18 | 0.028 | 0.024 | 0.094 |
| Phe60 | 62.38 | 0.835 | 65.43 | 0.979 | 69.08 | 0.044 | 64.96 | 0.993 | 68.33 | 0.037 | 64.19 | <0.001 | 0.079 | <0.001 |
| Phe90 | 63.54 | 0.190 | 68.35 | 0.981 | 72.63 | 0.017 | 67.75 | 0.997 | 71.24 | 0.040 | 67.13 | <0.001 | 0.133 | <0.001 |
| Phe120 | 63.63 | 0.023 | 68.33 | 0.998 | 73.64 | 0.150 | 69.33 | 1.000 | 73.07 | 0.114 | 69.18 | <0.001 | 0.421 | <0.001 |
| Phe150 | 63.25 | 0.013 | 67.70 | 0.992 | 74.20 | 0.027 | 69.99 | 0.952 | 72.58 | 0.088 | 68.76 | <0.001 | 0.219 | <0.001 |
| Phe180 | 63.58 | 0.165 | 68.62 | 1.000 | 72.84 | 0.303 | 68.20 | 1.000 | 69.48 | 0.989 | 68.40 | 0.002 | 0.987 | <0.001 |
| Phe300 | 58.46 | 0.755 | 60.49 | 0.999 | 64.33 | 0.807 | 57.76 | 0.463 | 59.73 | 0.951 | 61.40 | 0.055 | 0.220 | 0.051 |
| **AUC** | **18453.33** | **0.074** | **19562.08** | **1.000** | **20695.77** | **0.023** | **19410.61** | **1.000** | **19993.42** | **0.520** | **19452.21** | **<0.001** | **0.576** | **<0.001** |
| **iAUC** | **2174.06** | **0.050** | **3268.40** | **1.000** | **4389.63** | **0.030** | **3227.23** | **1.000** | **3787.09** | **0.425** | **3209.48** | **<0.001** | **0.516** | **<0.001** |
| Asp30 | 1.67 | 0.979 | 1.60 | 0.999 | 2.14 | 0.137 | 1.76 | 0.823 | 1.73 | 0.874 | 1.50 | 0.178 | 0.131 | 0.244 |
| Asp60 | 1.86 | 0.997 | 1.58 | 0.840 | 2.67 | 0.341 | 1.83 | 0.990 | 1.86 | 0.995 | 2.00 | 0.040 | 0.737 | 0.022 |
| Asp90 | 1.58 | 1.000 | 1.68 | 1.000 | 1.86 | 0.981 | 2.10 | 0.484 | 1.76 | 0.997 | 1.65 | 0.401 | 0.343 | 0.377 |
| Asp120 | 2.01 | 1.000 | 1.75 | 0.971 | 2.43 | 0.942 | 2.07 | 1.000 | 2.24 | 0.995 | 2.07 | 0.638 | 0.755 | 0.513 |
| Asp150 | 1.94 | 1.000 | 1.77 | 0.989 | 1.97 | 1.000 | 2.06 | 1.000 | 2.25 | 0.977 | 2.02 | 0.875 | 0.774 | 0.789 |
| Asp180 | 1.90 | 0.171 | 1.70 | 0.112 | 2.05 | 0.427 | 2.05 | 0.245 | 2.39 | 0.804 | 2.78 | 0.122 | 0.022 | 0.467 |
| Asp300 | 2.06 | 0.951 | 1.98 | 0.899 | 2.13 | 0.993 | 1.84 | 0.417 | 2.00 | 0.766 | 2.28 | 0.525 | 0.105 | 0.828 |
| **AUC** | **567.81** | **0.553** | **527.81** | **0.265** | **644.08** | **1.000** | **590.50** | **0.744** | **624.06** | **0.986** | **652.28** | **0.200** | **0.193** | **0.226** |
| **iAUC** | **61.90** | **0.217** | **25.03** | **0.069** | **81.93** | **0.539** | **61.37** | **0.124** | **115.11** | **0.863** | **153.86** | **0.072** | **0.020** | **0.285** |
| Cys30 | 251.35 | 0.882 | 254.23 | 0.329 | 253.83 | 0.368 | 252.07 | 0.640 | 253.84 | 0.157 | 248.73 | 0.180 | 0.021 | 0.690 |
| Cys60 | 246.28 | 0.984 | 250.19 | 0.366 | 249.61 | 0.445 | 249.77 | 0.230 | 250.97 | 0.058 | 244.58 | 0.055 | 0.008 | 0.395 |
| Cys90 | 241.21 | 0.987 | 247.15 | 0.114 | 244.71 | 0.445 | 246.05 | 0.083 | 244.94 | 0.179 | 239.55 | 0.034 | 0.008 | 0.237 |
| Cys120 | 237.95 | 0.896 | 244.86 | 0.048 | 241.05 | 0.391 | 239.76 | 0.457 | 241.04 | 0.177 | 234.77 | 0.061 | 0.014 | 0.303 |
| Cys150 | 234.01 | 0.928 | 242.21 | 0.020 | 236.44 | 0.563 | 238.51 | 0.086 | 236.98 | 0.228 | 231.18 | 0.018 | 0.007 | 0.128 |
| Cys180 | 233.27 | 0.955 | 241.68 | 0.075 | 232.44 | 0.991 | 234.09 | 0.840 | 234.69 | 0.706 | 230.12 | 0.116 | 0.113 | 0.166 |
| Cys300 | 228.39 | 0.792 | 255.36 | 0.015 | 233.82 | 1.000 | 237.49 | 0.996 | 236.42 | 1.000 | 235.13 | <0.001 | 0.765 | <0.001 |
| **AUC** | **71338.58** | **1.000** | **74418.83** | **0.015** | **72046.77** | **0.918** | **72422.40** | **0.563** | **72433.01** | **0.505** | **71142.96** | **0.014** | **0.062** | **0.023** |
| **iAUC** | **37.13** | **1.000** | **964.11** | **0.039** | **59.65** | **1.000** | **145.91** | **0.991** | **138.84** | **0.991** | **-5.43** | **0.032** | **0.414** | **0.021** |
| Glu30 | 26.93 | 1.000 | 26.05 | 0.997 | 30.47 | 0.965 | 29.53 | 0.989 | 28.63 | 1.000 | 27.76 | 0.788 | 0.674 | 0.695 |
| Glu60 | 26.80 | 0.257 | 25.59 | 0.296 | 29.60 | 0.764 | 28.38 | 0.375 | 26.49 | 0.103 | 34.76 | 0.120 | 0.007 | 0.839 |
| Glu90 | 26.07 | 0.679 | 26.38 | 0.853 | 29.41 | 0.996 | 33.26 | 0.989 | 29.42 | 0.989 | 31.29 | 0.388 | 0.617 | 0.293 |
| Glu120 | 31.85 | 1.000 | 26.68 | 0.971 | 31.94 | 1.000 | 33.50 | 0.991 | 32.64 | 0.998 | 31.00 | 0.844 | 0.651 | 0.770 |
| Glu150 | 28.09 | 0.991 | 29.73 | 1.000 | 29.28 | 1.000 | 32.63 | 0.992 | 33.67 | 0.945 | 30.51 | 0.738 | 0.637 | 0.644 |
| Glu180 | 29.32 | 0.841 | 27.16 | 0.735 | 25.56 | 0.431 | 31.26 | 0.955 | 36.59 | 0.997 | 34.78 | 0.256 | 0.484 | 0.197 |
| Glu300 | 34.18 | 0.848 | 26.93 | 1.000 | 26.82 | 0.999 | 27.46 | 1.000 | 29.30 | 1.000 | 28.67 | 0.638 | 0.862 | 0.503 |
| **AUC** | **8943.74** | **0.979** | **8187.39** | **0.712** | **8549.81** | **0.857** | **9212.31** | **0.998** | **9530.11** | **1.000** | **9490.31** | **0.673** | **0.608** | **0.578** |
| **iAUC** | **983.76** | **1.000** | **322.87** | **0.768** | **540.24** | **0.899** | **576.95** | **0.853** | **1470.18** | **0.903** | **1059.04** | **0.209** | **0.834** | **0.133** |
| Lys30 | 181.78 | 0.686 | 190.14 | 0.994 | 194.41 | 0.575 | 183.55 | 0.846 | 188.70 | 1.000 | 187.63 | 0.030 | 0.709 | 0.016 |
| Lys60 | 180.82 | 0.060 | 195.06 | 1.000 | 209.39 | 0.283 | 184.13 | 0.107 | 195.67 | 1.000 | 196.99 | <0.001 | 0.152 | <0.001 |
| Lys90 | 178.63 | 0.018 | 195.43 | 0.994 | 208.99 | 0.633 | 182.22 | 0.029 | 193.24 | 0.866 | 199.19 | <0.001 | 0.028 | <0.001 |
| Lys120 | 174.36 | 0.001 | 189.32 | 0.569 | 205.18 | 0.986 | 180.00 | 0.004 | 190.81 | 0.399 | 201.06 | <0.001 | 0.002 | <0.001 |
| Lys150 | 170.78 | 0.002 | 180.89 | 0.169 | 201.78 | 1.000 | 174.39 | 0.002 | 183.66 | 0.072 | 202.44 | <0.001 | <0.001 | 0.002 |
| Lys180 | 168.08 | <0.001 | 179.35 | 0.054 | 192.88 | 0.461 | 164.55 | <0.001 | 170.42 | <0.001 | 209.72 | <0.001 | <0.001 | 0.023 |
| Lys300 | 148.17 | <0.001 | 153.09 | 0.006 | 163.85 | 0.051 | 135.80 | <0.001 | 143.53 | <0.001 | 189.68 | <0.001 | <0.001 | 0.029 |
| **AUC** | **50635.34** | **<0.001** | **53686.23** | **0.032** | **57457.41** | **0.856** | **50290.27** | **<0.001** | **52495.15** | **<0.001** | **59290.73** | **<0.001** | **<0.001** | **<0.001** |
| **iAUC** | **1888.53** | **<0.001** | **4322.17** | **0.170** | **6866.21** | **0.935** | **2060.26** | **<0.001** | **3469.47** | **0.004** | **8205.86** | **<0.001** | **<0.001** | **0.005** |
| Arg30 | 96.60 | 0.997 | 99.59 | 0.824 | 101.11 | 0.542 | 101.87 | 0.226 | 106.55 | 0.003 | 95.02 | 0.005 | 0.003 | 0.059 |
| Arg60 | 99.62 | 1.000 | 107.01 | 0.730 | 115.65 | 0.023 | 114.03 | 0.013 | 118.56 | <0.001 | 100.16 | <0.001 | <0.001 | <0.001 |
| Arg90 | 101.29 | 0.999 | 111.87 | 0.633 | 117.46 | 0.107 | 119.49 | 0.009 | 120.86 | 0.002 | 103.10 | <0.001 | <0.001 | 0.002 |
| Arg120 | 102.10 | 0.990 | 109.15 | 0.987 | 119.40 | 0.161 | 124.43 | 0.003 | 125.51 | <0.001 | 105.30 | <0.001 | <0.001 | <0.001 |
| Arg150 | 97.83 | 0.832 | 104.56 | 1.000 | 116.27 | 0.417 | 122.52 | 0.014 | 121.64 | 0.014 | 104.66 | <0.001 | 0.006 | <0.001 |
| Arg180 | 97.41 | 0.542 | 106.22 | 1.000 | 111.93 | 0.977 | 117.37 | 0.413 | 114.87 | 0.668 | 107.32 | 0.014 | 0.266 | 0.011 |
| Arg300 | 92.63 | 1.000 | 93.00 | 1.000 | 96.24 | 0.995 | 96.96 | 0.977 | 96.00 | 0.990 | 92.77 | 0.947 | 0.542 | 0.940 |
| **AUC** | **29079.53** | **0.958** | **30786.14** | **0.997** | **32565.35** | **0.475** | **33479.06** | **0.053** | **33438.93** | **0.041** | **30132.33** | **<0.001** | **0.012** | **0.004** |
| **iAUC** | **3658.72** | **0.933** | **5405.79** | **0.998** | **7125.20** | **0.503** | **8109.97** | **0.049** | **8073.74** | **0.038** | **4810.53** | **<0.001** | **0.012** | **0.002** |
| His30 | 74.14 | 0.938 | 75.19 | 1.000 | 76.53 | 0.992 | 76.27 | 0.996 | 75.68 | 1.000 | 75.59 | 0.691 | 0.905 | 0.556 |
| His60 | 76.12 | 0.105 | 79.56 | 0.991 | 81.66 | 0.994 | 79.93 | 0.996 | 80.02 | 0.998 | 80.67 | 0.047 | 0.395 | 0.032 |
| His90 | 75.93 | 0.013 | 78.31 | 0.326 | 80.48 | 0.910 | 80.72 | 0.892 | 79.77 | 0.557 | 82.47 | 0.027 | 0.036 | 0.068 |
| His120 | 74.51 | <0.001 | 77.91 | 0.023 | 79.02 | 0.068 | 82.49 | 0.773 | 79.96 | 0.063 | 84.70 | <0.001 | 0.001 | 0.002 |
| His150 | 73.23 | <0.001 | 75.08 | <0.001 | 78.14 | 0.026 | 81.94 | 0.630 | 77.91 | 0.003 | 84.52 | <0.001 | <0.001 | <0.001 |
| His180 | 73.84 | <0.001 | 75.42 | 0.005 | 76.81 | 0.014 | 81.08 | 0.255 | 75.52 | <0.001 | 85.97 | <0.001 | <0.001 | 0.043 |
| His300 | 71.90 | 0.040 | 74.97 | 0.673 | 75.62 | 0.812 | 72.75 | 0.053 | 70.22 | <0.001 | 78.36 | 0.001 | <0.001 | 0.095 |
| **AUC** | **22154.72** | **<0.001** | **22818.58** | **0.008** | **23258.45** | **0.080** | **23629.79** | **0.221** | **22754.55** | **<0.001** | **24459.45** | **<0.001** | **<0.001** | **0.008** |
| **iAUC** | **885.22** | **<0.001** | **1624.62** | **0.051** | **1816.69** | **0.117** | **2157.32** | **0.291** | **1499.01** | **0.002** | **2898.11** | **<0.001** | **<0.001** | **0.027** |
| Gln30 | 517.71 | 1.000 | 526.15 | 0.986 | 525.88 | 0.984 | 526.92 | 0.942 | 537.68 | 0.368 | 518.21 | 0.422 | 0.205 | 0.499 |
| Gln60 | 533.48 | 1.000 | 560.11 | 0.560 | 570.91 | 0.140 | 560.92 | 0.264 | 576.46 | 0.018 | 534.34 | 0.007 | 0.011 | 0.035 |
| Gln90 | 535.54 | 0.968 | 558.89 | 0.984 | 571.76 | 0.654 | 559.24 | 0.948 | 579.14 | 0.216 | 547.30 | 0.070 | 0.197 | 0.070 |
| Gln120 | 527.75 | 0.223 | 547.73 | 0.961 | 566.17 | 1.000 | 570.01 | 0.991 | 582.09 | 0.703 | 562.04 | 0.019 | 0.859 | 0.010 |
| Gln150 | 527.37 | 0.219 | 532.61 | 0.558 | 560.68 | 1.000 | 566.43 | 0.995 | 569.38 | 0.977 | 559.82 | 0.023 | 0.842 | 0.012 |
| Gln180 | 523.17 | 0.214 | 544.61 | 0.979 | 555.05 | 1.000 | 558.37 | 1.000 | 553.79 | 1.000 | 556.46 | 0.172 | 0.477 | 0.126 |
| Gln300 | 509.95 | 0.447 | 533.70 | 1.000 | 537.29 | 1.000 | 525.94 | 0.952 | 533.52 | 0.999 | 538.26 | 0.456 | 0.304 | 0.459 |
| **AUC** | **156677.51** | **0.366** | **162190.02** | **1.000** | **165316.99** | **0.991** | **164891.02** | **0.992** | **######** | **0.897** | **163196.87** | **0.075** | **0.866** | **0.042** |
| **iAUC** | **8265.85** | **0.447** | **12686.12** | **0.999** | **15571.90** | **0.996** | **15567.14** | **0.991** | **17194.44** | **0.855** | **13918.55** | **0.093** | **0.809** | **0.054** |
| Tyr30 | 59.77 | 0.994 | 61.89 | 0.329 | 62.54 | 0.082 | 61.58 | 0.200 | 63.87 | <0.001 | 59.13 | <0.001 | <0.001 | 0.012 |
| Tyr60 | 61.24 | 0.843 | 65.92 | 0.936 | 69.89 | 0.072 | 66.59 | 0.631 | 73.00 | <0.001 | 63.70 | <0.001 | 0.002 | <0.001 |
| Tyr90 | 60.40 | 0.224 | 68.79 | 0.485 | 71.64 | 0.020 | 69.75 | 0.069 | 77.93 | <0.001 | 64.82 | <0.001 | <0.001 | <0.001 |
| Tyr120 | 58.36 | 0.034 | 67.91 | 0.987 | 72.17 | 0.197 | 70.96 | 0.253 | 81.60 | <0.001 | 66.09 | <0.001 | <0.001 | <0.001 |
| Tyr150 | 56.80 | 0.018 | 66.51 | 1.000 | 72.27 | 0.196 | 71.18 | 0.215 | 82.19 | <0.001 | 65.74 | <0.001 | <0.001 | <0.001 |
| Tyr180 | 56.07 | 0.015 | 66.18 | 1.000 | 70.49 | 0.850 | 69.22 | 0.942 | 78.83 | <0.001 | 66.71 | <0.001 | 0.056 | <0.001 |
| Tyr300 | 50.53 | 0.015 | 60.67 | 0.998 | 63.15 | 1.000 | 58.49 | 0.825 | 68.04 | 0.381 | 62.30 | <0.001 | 0.824 | <0.001 |
| **AUC** | **16982.35** | **0.009** | **19377.65** | **1.000** | **20381.68** | **0.398** | **19822.87** | **0.820** | **22190.08** | **<0.001** | **19161.40** | **<0.001** | **0.008** | **<0.001** |
| **iAUC** | **762.30** | **0.047** | **2388.95** | **1.000** | **3172.20** | **0.774** | **3034.30** | **0.800** | **5263.80** | **<0.001** | **2411.43** | **<0.001** | **0.006** | **<0.001** |
| Trp30 | 54.25 | 0.553 | 53.80 | 0.796 | 54.68 | 0.487 | 54.12 | 0.433 | 55.70 | 0.130 | 50.14 | 0.205 | 0.013 | 0.932 |
| Trp60 | 55.36 | 0.994 | 54.90 | 1.000 | 55.92 | 0.950 | 55.71 | 0.946 | 56.79 | 0.557 | 54.53 | 0.591 | 0.171 | 0.770 |
| Trp90 | 54.27 | 0.999 | 54.39 | 1.000 | 54.63 | 1.000 | 55.48 | 0.994 | 55.01 | 1.000 | 54.83 | 0.961 | 0.832 | 0.916 |
| Trp120 | 54.03 | 0.984 | 52.20 | 0.623 | 53.28 | 0.876 | 54.74 | 1.000 | 54.08 | 0.978 | 55.09 | 0.696 | 0.471 | 0.647 |
| Trp150 | 54.47 | 0.995 | 49.19 | 0.039 | 51.39 | 0.245 | 54.04 | 0.947 | 51.89 | 0.223 | 55.36 | 0.023 | 0.075 | 0.035 |
| Trp180 | 54.17 | 0.963 | 48.76 | 0.091 | 45.60 | <0.001 | 52.65 | 0.534 | 49.93 | 0.046 | 56.03 | <0.001 | 0.005 | 0.005 |
| Trp300 | 50.52 | 0.994 | 44.83 | 0.067 | 45.27 | 0.047 | 48.16 | 0.411 | 43.64 | <0.001 | 51.60 | <0.001 | 0.001 | 0.006 |
| **AUC** | **16024.47** | **1.000** | **15039.23** | **0.216** | **14993.86** | **0.098** | **15832.99** | **0.946** | **15325.43** | **0.235** | **16155.71** | **0.032** | **0.068** | **0.055** |
| **iAUC** | **1122.55** | **0.993** | **365.84** | **0.266** | **646.40** | **0.511** | **1096.66** | **0.978** | **732.03** | **0.482** | **1334.43** | **0.217** | **0.147** | **0.284** |

**Table S4. A)** Plasma concentration of Phe metabolites: benzoic acid, phenylpropionic acid, phenylacetic acid, phenylpyruric acid and phenyllactic acid as averages (n=10, ng/ml ± SD) at 0, 1, 3, 5 and 24 hour following the consumption of test meals and the statistical (ttest) evaluation of their trend over time

| **Meal** | **Timepoint** | **Phenylalanine plasma metabolites** **ng/ml ± SD (p value)** | | | | |
| --- | --- | --- | --- | --- | --- | --- |
|  |  | **benzoic acid** | **phenylpropionic acid** | **phenylacetic acid** | **phenylpyruvic acid** | **phenyllactic acid** |
| **Buckwheat** | **0H** | 5248±419.12 | 502.6±40.69 | 6254±414.41 | 7986±2279.98 | 9826±1404.09 |
|  | **1H (0 vs 1H)** | 5416±311.95(NS) | **568.8±81.19(<0.05)** | **6772±404.77(<0.05)** | 8698±3100.77(NS) | 10264±1578.46(NS) |
|  | **3H (0 vs 3H)** | 5498±262.03(NS) | **549.2±57.29(<0.05)** | **6852±433.2(<0.01)** | 7984±2793.74(NS) | 10240±1773.63(NS) |
|  | **5H (0 vs 5H)** | 5328±143.98(NS) | 501±59.29(NS) | **6620±351.76(<0.05)** | 8234±3154.1(NS) | 9790±1378.19(NS) |
|  | **24H (0 vs 24H)** | 5304±320.8(NS) | 529.6±93.79(NS) | 6396±647.99(NS) | 8262±2385.93(NS) | 9558±1673.23(NS) |
| **Fava bean** | **0H** | 1284.6±205.67 | 495.8±112.07 | 3392±491.55 | 5580±1455.23 | 9710±1071.13 |
|  | **1H (0 vs 1H)** | 1247±222.3(NS) | 522.8±104.07(NS) | 3440±525.95(NS) | 5132±1852.09(NS) | 9826±1378.41(NS) |
|  | **3H (0 vs 3H)** | 1255.2±114.4(NS) | 2395.6±4406.31(NS) | 3516±532.98(NS) | 6006±1630.16(NS) | 9088±1182.58(NS) |
|  | **5H (0 vs 5H)** | 1167.8±181.97(NS) | 481.4±91.61(NS) | 3434±399.34(NS) | 6474±1659.99(NS) | 9380±894.48(NS) |
|  | **24H (0 vs 24H)** | 1189.2±289.35(NS) | **678±85.53(<0.001)** | **3990±376.09(<0.01)** | **8880±1936.71(<0.001)** | **8540±450.09(<0.01)** |
| **Green pea** | **0H** | 3498±277.04 | 464.6±80.18 | 7580±541.27 | 12260±2203.19 | 15674±803.77 |
|  | **1H (0 vs 1H)** | 3480±228.62(NS) | 464.4±90.45(NS) | 7720±327.82(NS) | 11970±3407.69(NS) | 15820±722.03(NS) |
|  | **3H (0 vs 3H)** | 3464±132.26(NS) | 443±27.51(NS) | 7592±575.52(NS) | 11762±4038.79(NS) | 15754±1248.93(NS) |
|  | **5H (0 vs 5H)** | 3522±182.93(NS) | 436.4±34.26(NS) | 7996±594.55(NS) | 11914±2895.13(NS) | 16104±1374.62(NS) |
|  | **24H (0 vs 24H)** | 3578±191.94(NS) | 491.8±75.85(NS) | 7670±432.69(NS) | 11330±4243.6(NS) | 16158±938.06(NS) |
| **Hemp** | **0H** | 8538±632.21 | 303.6±29.71 | 7064±818.66 | 6396±748.91 | 10984±698.49 |
|  | **1H (0 vs 1H)** | 8118±440.05(NS) | 314.4±43.14(NS) | 6690±804.89(NS) | 5714±1554.38(NS) | 10554±1269.61(NS) |
|  | **3H (0 vs 3H)** | 8434±566.71(NS) | 308±30.8(NS) | 7140±1003.68(NS) | 6414±639.59(NS) | 11002±1272.95(NS) |
|  | **5H (0 vs 5H)** | 8375.56±507.82(NS) | 295.78±38.67(NS) | 6880±900(NS) | 5326.67±1432.97(NS) | 10740±1304.88(NS) |
|  | **24H (0 vs 24H)** | 8462.22±283.8(NS) | 318.67±54.21(NS) | 6875.56±527.43(NS) | 6540±629.44(NS) | 10906.67±546.26(NS) |
| **Lupin** | **0H** | 1418.8±127.82 | 477.8±61.71 | 3852±244.99 | 5144±1451.12 | 8114±1440.06 |
|  | **1H (0 vs 1H)** | 1321±177.34(NS) | 492.4±77.36(NS) | 4028±310.58(NS) | 6176±1955.96(NS) | 7666±911.27(NS) |
|  | **3H (0 vs 3H)** | 1308.4±108.95(NS) | 470.8±41.97(NS) | 4068±332.96(NS) | 5359±2132.58(NS) | 7868±855.32(NS) |
|  | **5H (0 vs 5H)** | 1337.6±77.83(NS) | 448.6±45.78(NS) | 3962±300.58(NS) | 5651.8±2400.21(NS) | 8060±1289.17(NS) |
|  | **24H (0 vs 24H)** | 1360.2±144.07(NS) | 477.2±82.4(NS) | 3948±298.02(NS) | 5268±2187.02(NS) | 7754±1095.57(NS) |
| **Meat** | **0H** | 1809±204.45() | 1137.2±139.16 | 5102±348.26 | 11224±3321.74 | 13620±1164.55 |
|  | **1H (0 vs 1H)** | 1839.4±215.37(NS) | 1199.6±167.93(NS) | 5264±297.52(NS) | **13550±814.28(<0.05)** | 14276±1167.17(NS) |
|  | **3H (0 vs 3H)** | 1810.2±143.72(NS) | 1175.2±168.38(NS) | 5294±708.11(NS) | 12256±3623.33(NS) | **14714±1030.56(<0.05)** |
|  | **5H (0 vs 5H)** | 1684.2±123.32(NS) | 1081.6±170.64(NS) | 5130±289.1(NS) | 12614±2869.05(NS) | 14228±1137.61(NS) |
|  | **24H (0 vs 24H)** | 1862.4±170.93(NS) | 1170±109.39(NS) | 5300±221.31(NS) | 11970±3056.99(NS) | 14640±1280.31(NS) |

**Table S4. B)** Plasma concentration of Tyr metabolites: p-hydroxybenzoic acid, 4-hydroxyphenylpropionic acid, 4-hydroxyphenylacetic acid, 4-hydroxyphenylpyruric acid, 4-hydroxyphenyllactic acid and p-cresol as averages (n=10, ng/ml ± SD) at 0, 1, 3, 5 and 24 hour following the consumption of test meals and the statistical (ttest) evaluation of their trend over time.

| **Meal** | **Timepoint** | **Tyrosine plasma metabolites ng/ml ± SD ( p value)** | | | | | |
| --- | --- | --- | --- | --- | --- | --- | --- |
|  |  | **p-hydroxybenzoic acid** | **4-hydroxyphenylpropionic acid** | **4-hydroxyphenylacetic acid** | **4-hydroxyphenylpyruvic acid** | **4-hydroxyphenyllactic acid** | **p-cresol** |
| **Buckwheat** | **0H** | 3004±303.87 | nd | 2460±283 | 34.6±109.41 | 1117±112.61 | 38542±17974.62 |
|  | **1H (0 vs 1H)** | 3234±220.92(NS) | nd | 2630±341.53(NS) | 13.96±44.15(NS) | 1277.2±268.5(NS) | 37980±17269.38(NS) |
|  | **3H (0 vs 3H)** | **3304±306.35(<0.05)** | nd | 2698±361.78(NS) | 171.24±416.7(NS) | 1281±257.07(NS) | 35406±15078.15(NS) |
|  | **5H (0 vs 5H)** | 3234±230.18(NS) | nd | 2580±327.41(NS) | nd | 1204.2±121.63(NS) | 36234±14736.06(NS) |
|  | **24H (0 vs 24H)** | 3050±194.19(NS) | nd | 2480±280.95(NS) | 43.26±93.1(NS) | 1186.2±127.03(NS) | 34594±16197.26(NS) |
| **Fava bean** | **0H** | 2378±188.43 | nd | 2804±1616.96 | 11636±4370.02 | 1702.8±402.31 | 4224±1603.66 |
|  | **1H (0 vs 1H)** | 2486±149.38(NS) | nd | 2857.4±1544.62(NS) | 10506±4803.42(NS) | 1786.2±375.88(NS) | 3916±1908.43(NS) |
|  | **3H (0 vs 3H)** | 2394±171.54(NS) | nd | 2807.8±1632.9(NS) | 10634±3911.93(NS) | 1695.2±336.31(NS) | 3721.4±1605.59(NS) |
|  | **5H (0 vs 5H)** | 2363.6±171.65(NS) | nd | 3016±1344.81(NS) | 10494±4885.53(NS) | 1716.4±404.19(NS) | 6616.2±10663.05(NS) |
|  | **24H (0 vs 24H)** | 2169.4±301.75(NS) | nd | **5634±632.11(<0.001)** | **3874±618.1(<0.001)** | **2374±261.63(<0.001)** | **18018±9857.73(<0.001)** |
| **Green pea** | **0H** | 2772±196 | nd | 3412±267.37 | nd | 1248.2±159.17 | 846±912.33 |
|  | **1H (0 vs 1H)** | 2814±131(NS) | nd | 3478±199.21(NS) | nd | 1260.6±104.59(NS) | 835±938.01(NS) |
|  | **3H (0 vs 3H)** | 2760±156.35(NS) | nd | 3366±198.9(NS) | nd | 1244.2±118.79(NS) | 902.4±1002.99(NS) |
|  | **5H (0 vs 5H)** | 2812±170.28(NS) | nd | 3502±185.1(NS) | nd | 1290.8±184.31(NS) | 671.4±915.25(NS) |
|  | **24H (0 vs 24H)** | 2772±126.21(NS) | nd | 3508±234.42(NS) | nd | 1279.6±193.94(NS) | 775.2±1421.1(NS) |
| **Hemp** | **0H** | 2536±197.94 | nd | 1558.2±147.17 | 38480±11909.73 | 929±91.87 | 3060.2±1370.21 |
|  | **1H (0 vs 1H)** | 2392±109.63(NS) | nd | 1494.2±44.18(NS) | 38500±9620.93(NS) | 873.8±95.75(NS) | 3149.6±1659.61(NS) |
|  | **3H (0 vs 3H)** | 2510±147.95(NS) | nd | 1514.8±174.57(NS) | 47800±12256.52(NS) | 926.8±90.13(NS) | 2795.6±1473.98(NS) |
|  | **5H (0 vs 5H)** | 2464.44±143.8(NS) | nd | 1532.89±47.59(NS) | 46755.56±10162.57(NS) | 985.56±78.8(NS) | 2868.67±1650.85(NS) |
|  | **24H (0 vs 24H)** | 2500±105.83(NS) | nd | 1553.78±102.6(NS) | 44555.56±17950.7(NS) | 949.33±63.53(NS) | 2423.78±1182.38(NS) |
| **Lupin** | **0H** | 2070.8±154.9 | nd | 2596±244.19 | nd | 1911±254.87 | 21465±21402.47 |
|  | **1H (0 vs 1H)** | 2100.8±143.33(NS) | nd | 2570±198.94(NS) | nd | 1794.4±253.29(NS) | 19510±18577.76(NS) |
|  | **3H (0 vs 3H)** | 2150±90.06(NS) | nd | 2672±136.69(NS) | nd | 2024.4±239.59(NS) | 18402±19053.84(NS) |
|  | **5H (0 vs 5H)** | 2106.2±118.67(NS) | nd | 2580±254.21(NS) | nd | 1946±288.69(NS) | 17388±18057.68(NS) |
|  | **24H (0 vs 24H)** | 2095.2±274.38(NS) | nd | 2542±209.86(NS) | nd | 1933.8±255.95(NS) | 19184±17570.17(NS) |
| **Meat** | **0H** | 2828±144.28 | nd | 3586±283.64 | nd | 2095.8±221.54 | 850.18±521.07 |
|  | **1H (0 vs 1H)** | 2866±124.03(NS) | nd | 3776±263.62(NS) | nd | 2090±214.95(NS) | 713.8±349.1(NS) |
|  | **3H (0 vs 3H)** | 2992±203.79(NS) | nd | 3896±397.97(NS) | nd | 2298±434.86(NS) | 873.8±663.68(NS) |
|  | **5H (0 vs 5H)** | 2876±139.46(NS) | nd | 3566±294.25(NS) | nd | 2108.4±169.01(NS) | 616.6±169.13(NS) |
|  | **24H (0 vs 24H)** | **2958±105.18(<0.05)** | nd | 3722±334.33(NS) | nd | 2143.8±217.87(NS) | 698.4±355.9(NS) |

**Table S4 C)** plasma concentration of Trp metabolites: indole-3-acetic acid, indole-3-propionic acid, indole-3-carboxylic acid, indole-3-pyruric acid, indole-3-lactic acid and indole 4 methyl as averages (n=10, ng/ml ± SD) at 0, 1, 3, 5 and 24 hour following the consumption of test meals and the statistical (ttest) evaluation of their trend over time.

| **Meal** | **Timepoint** | **Tryptophan plasma metabolites ng/ml ± SD ( p value)** | | | | | |
| --- | --- | --- | --- | --- | --- | --- | --- |
|  |  | **indole-3-acetic acid** | **indole-3-propionic acid acid** | **indole-3-carboxylic acid** | **indole-3-pyruvic acid** | **indole-3-methyl** | **indoe-3-lactic acid** |
| **Buckwheat** | **0H** | 669±75.71 | 130.34±40.46 | 291±26.64 | 11800±2670.26 | nd | 1852.8±1744.38 |
|  | **1H (0 vs 1H)** | 722.6±69.37(NS) | 141.66±52.09(NS) | 313.2±20.94(NS) | 12650±3693.48(NS) | nd | 1552.2±828.86(NS) |
|  | **3H (0 vs 3H)** | 715.4±96.84(NS) | 133.86±54.84(NS) | 312±23.61(NS) | 13470±2515.66(NS) | nd | 1746±1041.42(NS) |
|  | **5H (0 vs 5H)** | 657.2±71.6(NS) | 123.6±54.26(NS) | 299.8±15.16(NS) | 12678±2369.33(NS) | nd | 1829.4±997.03(NS) |
|  | **24H (0 vs 24H)** | 670.6±42.08(NS) | **208.92±100.85(<0.05)** | 296.6±16.44(NS) | 11610±2433.2(NS) | nd | 1401±298.43(NS) |
| **Fava bean** | **0H** | 954±116.58 | 263.5±199.61 | 114.72±16.34 | 380200±95193.14 | nd | 737±276.19 |
|  | **1H (0 vs 1H)** | 959.2±135.32(NS) | 275.32±207.68(NS) | 116.1±15.44(NS) | 386000±90037.03(NS) | nd | 723.2±286.18(NS) |
|  | **3H (0 vs 3H)** | 894.8±132.66(NS) | 268.74±216.29(NS) | 114.22±15.61(NS) | 345200±83948.13(NS) | nd | 776.8±289.02(NS) |
|  | **5H (0 vs 5H)** | 855±123.37(NS) | 237.96±180.14(NS) | 119.24±15.8(NS) | 393600±106093.46(NS) | 13.62±43.07(NS) | 759.2±369.14(NS) |
|  | **24H (0 vs 24H)** | **1077.2±126.02(<0.05)** | 324.7±184.92(NS) | **150.74±15.33(<0.001)** | 371000±39924.93(NS) | nd | **1740±667.06(<0.001)** |
| **Green pea** | **0H** | 815.4±81.48 | 179.24±108 | 250.6±17.08 | 25020±41916.26 | nd | 538.6±49.15 |
|  | **1H (0 vs 1H)** | 830.2±56.4(NS) | 175.28±112.63(NS) | 254.2±12.73(NS) | 55460±52645.23(NS) | nd | 586.8±241.87(NS) |
|  | **3H (0 vs 3H)** | 765.2±67.73(NS) | 166.84±109.84(NS) | 245.6±13.88(NS) | 28980±38121.03(NS) | nd | 790±530.9(NS) |
|  | **5H (0 vs 5H)** | **739.4±53.44(<0.05)** | 159.7±98.99(NS) | 250±14.88(NS) | 51380±38480.18(NS) | nd | 547.4±164.31(NS) |
|  | **24H (0 vs 24H)** | 792.8±69.6(NS) | **306.5±127.46(<0.05)** | 246.8±12.48(NS) | 36980±49126(NS) | nd | 799.4±566.42(NS) |
| **Hemp** | **0H** | 648.2±74.82 | 104.8±73.16 | 201.2±12.19 | 647800±146483.07 | nd | 906±295.71 |
|  | **1H (0 vs 1H)** | 626±79.77(NS) | 98.28±66.31(NS) | **189.38±9.4(<0.05)** | 619200±162295.2(NS) | nd | 918.4±330.36(NS) |
|  | **3H (0 vs 3H)** | 634.8±93.01(NS) | 91.54±65.01(NS) | 195.96±12.56(NS) | 774600±179051.95(NS) | nd | 878.8±209.97(NS) |
|  | **5H (0 vs 5H)** | 599.11±53.26(NS) | 94.56±73.91(NS) | 191.38±12.01(NS) | 768444.44±138136.81(NS) | nd | 963.33±277.83(NS) |
|  | **24H (0 vs 24H)** | 633.56±80.48(NS) | 178.02±118.65(NS) | 196.76±7.21(NS) | 743777.78±238282.28(NS) | nd | 917.56±262.88(NS) |
| **Lupin** | **0H** | 607±69.26 | 178.98±84.78 | 144.6±10.89 | 6620±14146.05 | nd | 693.8±89.47 |
|  | **1H (0 vs 1H)** | 617.4±60.02(NS) | 181.6±88.07(NS) | 149.34±8.05(NS) | 9460±15990.01(NS) | nd | 650.8±86.8(NS) |
|  | **3H (0 vs 3H)** | 597.4±47(NS) | 151.42±69.9(NS) | 148.62±7.23(NS) | 9680±15866.16(NS) | nd | 800.8±350.25(NS) |
|  | **5H (0 vs 5H)** | 548.4±62.99(NS) | 138.34±64.87(NS) | 146.62±7.45(NS) | 13180±17483.31(NS) | nd | 749.4±346.84(NS) |
|  | **24H (0 vs 24H)** | 567.8±47.96(NS) | **303.12±141.59(<0.05)** | 142.18±11.21(NS) | 9840±21051.64(NS) | nd | 703.8±172.25(NS) |
| **Meat** | **0H** | 631.2±63.6 | 165.34±81.65 | 211.8±12.31 | 30460±8034.4 | nd | 1645.2±583.18 |
|  | **1H (0 vs 1H)** | 645.4±52.8(NS) | 167.4±86.24(NS) | 219±7.2(NS) | **43900±12738.83(<0.05)** | nd | 1381.2±238.23(NS) |
|  | **3H (0 vs 3H)** | 646.2±53.37(NS) | 153.24±75.4(NS) | **223.6±12.89(<0.05)** | **54820±14589.33(<0.001)** | nd | 1395.4±278.05(NS) |
|  | **5H (0 vs 5H)** | 596±60.21(NS) | 140.38±78.79(NS) | 215±8.76(NS) | **43620±10042.78(<0.01)** | nd | 1252.6±199.42(NS) |
|  | **24H (0 vs 24H)** | 649±55.85(NS) | 155.48±63.43(NS) | **226.8±6.05(<0.01)** | 272100±754731.71(NS) | nd | 1686.4±1100.7(NS) |

**Table S5.** Plasma concentration (ng/mL± SD, n=10) of plant metabolites with a significant change (t test) from baseline (0 h) and 1, 3, 5 h and 24 h after consumption of the intervention meals: buckwheat, fava bean, green pea, hemp, lupin and meat.

| **Metabolite** | **Plasma concentration ng/mL ± SD ( p value, as T test vs baseline)** | | | | |
| --- | --- | --- | --- | --- | --- |
|  | **0H** | **1 H** | **3H** | **5H** | **24H** |
| **BUCKWHEAT MEAL** | | | | | |
| ferulic acid | 39.36±9.98 | **50.38±6.93(0.01)** | **50.46±6.19(<0.01)** | 44.11±10.95(NS) | 40.54±7.98(NS) |
| salicylic acid | 134.34±27.1 | **186.82±52.74(<0.05)** | **274.2±37.65(<0.001)** | **323.8±55.16(<0.001)** | **287.48±217.95(<0.05)** |
| vanillic acid | 725±95.42 | 805±83.58(NS) | **837±64.14(<0.01)** | **802.4±63.95(<0.05)** | 737±75.6(NS) |
| syringic acid | 53.34±6.14 | 57.66±10.69(NS) | **62.16±5.48(<0.01)** | 57.08±6.33(NS) | 50.66±4.29(NS) |
| 3,4-dimethoxybenzoic acid | 23.16±4.17 | **27.16±3.44(<0.05)** | **26.96±3.35(<0.05)** | 26.1±3.25(NS) | 24.14±2.69(NS) |
| daidzein | 63.5±4.38 | 66.52±5.66(NS) | **68.62±5.13(<0.05)** | 64.72±4.71(NS) | 66.18±5.08(NS) |
| 3,4-dimethoxycinnamic acid | 28.4±2.1 | **31.54±4.09(<0.05)** | 29.56±3.34(NS) | 27.86±2.75(NS) | 27.54±7.03(NS) |
| 2-hydroxyphenylpropionic acid | 25.82±2.09 | 28.06±2.81(NS) | **28.7±2.33(<0.01)** | 27.92±2.39(NS) | 27.18±3.2(NS) |
| quinadilic acid | 19.12±6 | 19.37±5.51(NS) | 18.61±5.45(NS) | 18.75±5.33(NS) | **32.67±15.79(<0.05)** |
| o-hydroxyhippuric acid | 8.39±7.38 | 17.7±15.47(NS) | **33.63±12.68(<0.001)** | **53.82±15.2(<0.001)** | **57.7±44.35(<0.01)** |
| phenol | 16906±4835.5 | 21936±6815.26(NS) | **29600±6637.27(<0.001)** | **37940±8551.05(<0.001)** | 28872±20416.77(NS) |
| mandelic acid | 11022±948.1 | **12042±768.81(<0.05)** | **12478±684.07(0.001)** | **11812±556.03(<0.05)** | 11308±1135.54(NS) |
| **FAVA BEAN MEAL** | | | | | |
| cinnamic acid | 78±8.03 | 76.22±15.28(NS) | 90.26±26.23(NS) | 78.1±16.58(NS) | **99.6±13.03(<0.001)** |
| ferulic acid | 48.5±9.78 | 57.96±14.87(NS) | **58.88±10.16(<0.05)** | 55.28±9.81(NS) | 50.04±15.03(NS) |
| 2,5-dihydroxybenzoic acid | 45.32±12.38 | 50.02±11.12(NS) | 47.58±14.1(NS) | 44.14±13.95(NS) | **20.24±6.59(<0.001)** |
| p-hydroxybenzaldehyde | 67.3±28.61 | 61.84±26.92(NS) | 65.02±30.09(NS) | 75.2±34.23(NS) | **133.16±14.83(<0.001)** |
| protocatachaldehyde | 0.64±2.02 | 0.78±2.45(NS) | 0.57±1.8(NS) | 1.12±2.41(NS) | **6.29±1.68(<0.001)** |
| ferulic dimer (8-5 linked) | 142.52±28.49 | 150.2±32.6(NS) | 150.86±33.62(NS) | 137.32±41.56(NS) | **65.38±6.09(<0.001)** |
| hydrogenated Ferulic Dimer H5-5 | 14.68±1.8 | 14.69±1.79(NS) | 14.87±3.12(NS) | 14.29±2.53(NS) | **9.98±0.7(<0.001)** |
| spermidine | 3670±538.33 | 3836±475.89(NS) | 3746±724.92(NS) | 3740±641.11(NS) | **5078±338.95(<0.001)** |
| cadaverine | 4400±911.9 | 4578±1056.09(NS) | 4782±720.27(NS) | 4678±735.84(NS) | **5468±469.63(<0.01)** |
| putresceine | 10536±2505.65 | 10868±2629.39(NS) | 10574±2319.45(NS) | 10978±2904.82(NS) | **14718±1056.93(<0.001)** |
| 8-methylpsoralen | 43.88±7.54 | 43.44±7.18(NS) | 44.3±10.15(NS) | 45.12±7.89(NS) | **64.88±5.59(<0.01)** |
| bergapten | 0.62±0.81 | 0.59±0.95(NS) | 0.69±0.91(NS) | 0.61±1.02(NS) | **0±0(<0.05)** |
| tangeretin | 4.29±0.92 | 4.29±1.11(NS) | 4.5±1.04(NS) | 4.13±0.95(NS) | **3.57±0.53(<0.05)** |
| coumesterol | 12.58±4.65 | 12.47±4.65(NS) | 13.21±4.93(NS) | 12.2±6.99(NS) | **0±0(<0.001)** |
| isoliquiritigenin | 2.15±0.9 | 2.41±1(NS) | 1.82±1.02(NS) | 2.37±1.14(NS) | **3.34±0.57(<0.01)** |
| imperatorin | 14.09±3.23 | 14.84±3.39(NS) | 15.13±4.39(NS) | 15.74±3.68(NS) | **23.88±2.42(<0.001)** |
| taxifolin | 21.02±2.95 | 21.23±3.98(NS) | 22.39±4.74(NS) | 22.19±3.71(NS) | **16.51±2.82(<0.01)** |
| genstein | 156.46±29.3 | 169±31.89(NS) | 157.58±29.73(NS) | 174.42±31.71(NS) | **234.22±40.19(<0.001)** |
| scopoletin | 75.94±10.02 | 78.96±9.19(NS) | 80.56±14.26(NS) | 77.1±12.97(NS) | **63.22±6.14(<0.01)** |
| 7,8-dihydroxy-6-methyl coumarin | 41.59±9.85 | 44.61±11.85(NS) | 41.81±11.47(NS) | 38.82±14.03(NS) | **19±5.13(<0.001)** |
| phloridzin | 129.14±24.78 | 127.62±24.62(NS) | 123.38±23.54(NS) | 134.46±19.61(NS) | **175.96±28.87(<0.01)** |
| fisetin | 13.24±9.43 | 18.73±6.93(NS) | 15.57±8.55(NS) | 13.2±9.41(NS) | **0±0(<0.001)** |
| isorhamnetin | 557.8±95.09 | 580.2±83.42(NS) | 591±120.9(NS) | 650.6±121.58(NS) | **855.2±82.67(<0.001)** |
| formononetin | 2213.2±305.51 | 2337.6±396.65(NS) | 2322.4±367.05(NS) | 2492±439.92(NS) | **3512±404(<0.001)** |
| secoisolariciresinol | 57.72±10.7 | 58.5±12.03(NS) | 57.96±10.41(NS) | 54.72±10.94(NS) | **43.04±3.68(<0.001)** |
| hydroxymatairesinol | 189.64±72.38 | 198.12±75.26(NS) | 196.58±75.2(NS) | 170.04±93.05(NS) | **0±0(<0.001)** |
| 2-hydroxyphenylpropionic acid | 26.44±2.75 | 26.74±3.23(NS) | **23.91±2.64(<0.05)** | 24.04±3.62(NS) | **22.21±2.78(<0.01)** |
| 3,4-dihydroxyphenylacetic acid | 157.54±62.63 | 202.88±108.33(NS) | 157.92±81.19(NS) | 153.54±91.78(NS) | **9.5±30.04(<0.001)** |
| 1,2-hydroxybenzene | 160.14±64.07 | 169.58±57(NS) | 158.86±36.86(NS) | 141.1±37.2(NS) | **86.68±30.4(<0.01)** |
| 4-hydroxyacetophenone | 0.44±1.38 | 0.63±1.99(NS) | 0.63±2(NS) | 1.2±2.57(NS) | **5.76±0.88(<0.001)** |
| 4-hydroxy-3-methoxyacetophenone | 6.25±8.07 | 5.01±8.23(NS) | 4±6.76(NS) | 5.88±7.84(NS) | **14.19±3.17(<0.01)** |
| tyramine | 7278±2146.66 | 7742±1906.05(NS) | 7612±1824.16(NS) | 7838±2159.45(NS) | **10366±1155.47(<0.001)** |
| 4-methylcatechol | 14.11±11.1 | 16.41±13.95(NS) | 12.45±8.29(NS) | 12.58±9.74(NS) | **1.3±4.12(<0.01)** |
| histamine | 357.2±73.61 | 378.6±77.05(NS) | 386.2±50.81(NS) | 391.2±67.61(NS) | **499.2±60.34(<0.001)** |
| 5-OHtryptophan | 14.31±3.98 | 18±9.34(NS) | 18.63±5.71(NS) | 14.53±3.53(NS) | **18.09±4.06(<0.05)** |
| **GREEN PEA MEAL** | | | | | |
| ferulic acid | 61.8±11.45 | 67.08±19.27(NS) | **72.52±6.58(<0.05)** | 61.64±18(NS) | 64.04±7.1(NS) |
| 2-hydroxyphenylpropionic acid | 25.24±2.47 | 26.36±3.24(NS) | **27.96±3.21(<0.05)** | 26.6±2.33(NS) | 26.96±1.32(NS) |
| 4-methoxy phenyl acetic acid | 35.68±3.62 | **40.82±3.36(<0.01)** | **40.82±4.81(<0.05)** | **39.9±4.66(<0.05)** | **40.3±4.9(<0.05)** |
| coumarin | 8.56±1.36 | 7.92±0.71(NS) | **7.47±0.77(<0.05)** | 7.98±0.66(NS) | 9.4±4.49(NS) |
| **HEMP MEAL** | | | | | |
| p-coumaric acid | 11.83±2.02 | **14.18±2.51(<0.05)** | **17.34±3.2(<0.001)** | **14.41±2.82(<0.05)** | 12.88±3.43(NS) |
| ferulic acid | 24.72±2.14 | **31.94±3.66(<0.001)** | **35.14±4.46(<0.001)** | **31.29±2.83(<0.001)** | 25.16±2.05(NS) |
| salicylic acid | 158.54±40.17 | **216.74±55.5(<0.05)** | **232.12±54.89(<0.01)** | **199.62±39.65(<0.05)** | 160.58±44.19(NS) |
| isorhamnetin | 17.83±2.59 | 20.56±7.78(NS) | 18.6±2.43(NS) | **21.45±4.68(<0.05)** | 17.32±2.17(NS) |
| **LUPIN MEAL** | | | | | |
| p-hydroxybenzaldehyde | 153.44±23.36 | **178.2±23.24(<0.05)** | 204.46±91.78(NS) | **178.32±20.86(<0.05)** | 214.8±131.85(NS) |
| isoliquiritigenin | 1.74±0.33 | 1.71±0.29(NS) | **2.06±0.34(<0.05)** | 1.79±0.33(NS) | 1.8±0.25(NS) |
| biochanin A | 127.04±7.17 | 122.64±18.32(NS) | 126.96±16.73(NS) | 126.28±10.65(NS) | **119.22±7.11(<0.05)** |
| 3-hydroxyphenylpropionic acid | 31.6±10.24 | 29.57±11.5(NS) | **23.03±5.22(<0.05)** | **20.22±5.17(<0.01)** | 42.03±26.96(NS) |
| **MEAT MEAL** | | | | | |
| ferulic acid | 60.98±5.97 | **80.7±10.25(<0.001)** | **80.6±6.09(<0.001)** | **68.56±7.97(<0.05)** | **68.22±4.31(<0.01)** |
| sinapic acid | 36.5±5.15 | 36.76±5.25(NS) | 39.06±4.18(NS) | 37.84±3.82(NS) | 37.34±3.4(NS) |
| vanillic acid | 896.2±57.88 | **951.4±52.52(<0.05)** | **974.8±89.16(<0.05)** | 916±72.14(NS) | 936±83.21(NS) |
| syringic acid | 102.12±10.63 | 105.74±12.48(NS) | **113.3±12.33(<0.05)** | 102.7±8.11(NS) | 102.72±13.68(NS) |
| 8-methylpsoralen | 65.42±5.61 | 70.6±5.66(NS) | 69.6±6(NS) | 66.7±7.9(NS) | **71.46±6.69(<0.05)** |
| scopoletin | 77.96±5.27 | **84.64±6.34(<0.05)** | **87.98±6.18(0.001)** | 81.42±7.11(NS) | 81.88±7.37(NS) |
| matairesinol | 63.66±3.69 | 66.74±6.66(NS) | **71.18±4.15(<0.001)** | 67.28±4.22(NS) | 65.16±4.98(NS) |
| syringaresinol | 220.02±24.88 | 229.52±52.09(NS) | 234.78±24.39(NS) | 246.4±37.5(NS) | **247±30.89(<0.05)** |
| pinoresinol | 248.8±20.05 | 262.4±16.91(NS) | **278±17.69(<0.01)** | 262.2±7.91(NS) | **272±25.19(<0.05)** |
| 4-hydroxy-3-methoxyphenylpropionic acid | 1625.2±110.82 | 1680.4±81.31(NS) | **1772.8±174.37(<0.05)** | 1652.4±140.87(NS) | 1658.8±115.33(NS) |
| mandelic acid | 3194±134.35 | 3278±155.33(NS) | **3530±338.26(<0.01)** | 3274±224.11(NS) | 3320±238.89(NS) |

**Table S6 A)** Several plasma metabolites highlighted by PLS DA analysis potentially associated with the GLP1 significantly different after hemp vs meat meals consumption at 60 and 180 minutes

| **Plasma metabolites correlated with GLP-1** | **TEST MEALS** | | **P values (ttest)** | **TEST MEALS** | | **P values (ttest)** |
| --- | --- | --- | --- | --- | --- | --- |
|  | **60 mins** | | **60 mins** | **180 mins** | | **180 mins** |
|  | **Hemp** | **Meat** | **Hemp vs Meat** | **Hemp** | **Meat** | **Hemp vs Meat** |
| **3-hydroxymandelic acid** | 8.05±1.53 | 0±0 | <0.001 | 8.514±1.31 | 2.428±5.12 | <0.01 |
| **4-hydroxyphenylpyruvic acid** | 38500±9620 | 0±0 | <0.001 | 47800±12256 | 0±0 | <0.001 |
| **hydrogenated Ferulic Dimer H5-5** | 13.14±1.39 | 15.57±3.35 | <0.05 | 13.302±1.45 | 15.534±2.66 | <0.05 |
| **indole-3-pyruvic acid** | 619200±162295 | 43900±12738 | <0.001 | 774600±179051 | 54820±14589 | <0.001 |
| **piperidine** | 525±59.13 | 1298±77.67 | <0.001 | 550±49.68 | 1248.6±123.5 | <0.001 |
| **5-OHtryptophan** | 19.64±2.97 | 0.46±1.44 | <0.001 | 18.588±2.66 | 0±0 | <0.001 |
| **genstein** | 160.24±15.87 | 82.8±21.3 | <0.001 | 164.46±8.34 | 80.42±16.76 | <0.001 |
| **biochanin A** | 104.38±10.5 | 55.12±11.38 | <0.001 | 111.38±9.25 | 54.62±12.32 | <0.001 |
| **luteolinidin** | 16.46±1.96 | 0±0 | <0.001 | 15.172±5.58 | 0±0 | <0.001 |

**Table S6 B)** Several plasma metabolites highlighted by PLS DA analysis potentially associated with the Insulin significantly different after hemp vs meat meals consumption at 60 and 180 minutes

| **Plasma metabolites correlated with Insulin** | **TEST MEALS** | | **P values (ttest)** | **TEST MEAL** | | **P values (ttest)** |
| --- | --- | --- | --- | --- | --- | --- |
|  | **60 mins** | | **60 mins** | **180 mins** | | **180 mins** |
|  | **Hemp** | **Meat** | **Hemp vs Meat** | **Hemp** | **Meat** | **Hemp vs Meat** |
| **benzoic acid** | 8118±440.05 | 1839±215.37 | <0.001 | 8434±566.71 | 1810±143.72 | <0.001 |
| **3-hydroxymandelic acid** | 8.05±1.53 | 0±0 | <0.001 | 8.51±1.31 | 2.43±5.12 | <0.01 |
| **4-hydroxymandelic acid** | 2680±296.95 | 603.4±68.39 | <0.001 | 2902±331.92 | 655.2±141 | <0.001 |
| **4-hydroxyphenylpyruvic acid** | 38500±9620 | 0±0 | <0.001 | 47800±12256 | 0±0 | <0.001 |
| **indole-3-pyruvic acid** | 619200±162295 | 43900±12738 | <0.001 | 774600±179051 | 54820±14589 | <0.001 |
| **5-OHtryptophan** | 19.64±2.97 | 0.46±1.44 | <0.001 | 18.59±2.66 | 0±0 | <0.001 |
| **coumesterol** | 21.91±9.18 | 0±0 | <0.001 | 23.29±8.05 | 0±0 | <0.001 |
| **genstein** | 160.24±15.87 | 82.8±21.3 | <0.001 | 164.46±8.34 | 80.42±16.76 | <0.001 |
| **biochanin A** | 104.38±10.5 | 55.12±11.38 | <0.001 | 111.38±9.25 | 54.62±12.32 | <0.001 |
| **luteolinidin** | 16.46±1.96 | 0±0 | <0.001 | 15.17±5.58 | 0±0 | <0.001 |
| **secoisolariciresinol** | 194.5±23.56 | 136.76±24.7 | <0.001 | 203.26±26.04 | 142.76±21.75 | <0.001 |

**Table S6 C)** Several plasma metabolites highlighted by PLS DA analysis potentially associated with the Ghrelin significantly different after hemp vs meat meals consumption at 60 and 180 minutes

| **Plasma metabolites correlated with Ghrelin** | **TEST MEALS** | | **P values (ttest)** | **TEST MEAL** | | **P values (ttest)** |
| --- | --- | --- | --- | --- | --- | --- |
|  | **60 mins** | | **60 mins** | **180 mins** | | **180 mins** |
|  | **Hemp** | **Meat** | **Hemp vs Meat** | **Hemp** | **Meat** | **Hemp vs Meat** |
| **benzoic acid** | 8118±440.05 | 1839±215.37 | <0.001 | 8434±566.71 | 1810±143.72 | <0.001 |
| **p-coumaric acid** | 14.18±2.51 | 3.28±10.37 | <0.01 | 17.34±3.2 | 4.86±11.07 | <0.01 |
| **phenylacetic acid** | 6690±804.89 | 5264±297.52 | <0.001 | 7140±1003 | 5294±708.11 | <0.001 |
| **mandelic acid** | 10998±714.76 | 3278±155.33 | <0.001 | 11402±755.86 | 3530±338.26 | <0.001 |
| **3-hydroxymandelic acid** | 8.05±1.53 | 0±0 | <0.001 | 8.51±1.31 | 2.43±5.12 | <0.01 |
| **4-hydroxymandelic acid** | 2680±296.95 | 603.4±68.39 | <0.001 | 2902±331.92 | 655.2±141 | <0.001 |
| **daidzein** | 80.58±9.73 | 64.46±14.57 | <0.01 | 81.62±6.6 | 63.58±9.46 | <0.001 |
| **luteolin** | 2670±193.74 | 1861±195.26 | <0.001 | 2708±145.82 | 1843±190.01 | <0.001 |
| **luteolinidin** | 16.46±1.96 | 0±0 | <0.001 | 15.17±5.58 | 0±0 | <0.001 |
| **apigenin** | 2556±145.08 | 1763±252.63 | <0.001 | 2702±195.83 | 1741±199.62 | <0.001 |
| **secoisolariciresinol** | 194.5±23.56 | 136.76±24.7 | <0.001 | 203.26±26.04 | 142.76±21.75 | <0.001 |
| **matairesinol** | 77.24±8.28 | 66.74±6.66 | <0.01 | 81.2±12.91 | 71.18±4.15 | <0.05 |
| **pinoresinol** | 319±39.51 | 262.4±16.91 | <0.001 | 331.4±39.2 | 278±17.69 | <0.001 |

**Table S6 D)** Several plasma metabolites highlighted by PLS DA analysis potentially associated with the PYY significantly different after hemp vs meat meals consumption at 60 and 180 minutes

| **Plasma metabolites correlated with PYY** | **TEST MEALS** | | **P values (ttest)** | **TEST MEAL** | | **P values (ttest)** |
| --- | --- | --- | --- | --- | --- | --- |
|  | **60 mins** | | **60 mins** | **180 mins** | | **180 mins** |
|  | **Hemp** | **Meat** | **Hemp vs Meat** | **Hemp** | **Meat** | **Hemp vs Meat** |
| **4-hydroxyphenylpyruvic acid**  **indole-3-pyruvic acid**  **5-OHtryptophan**  **coumesterol**  **genstein**  **biochanin A**  **ferulic dimer (8-5 linked)** | 38500±9620 | 0±0 | <0.001 | 47800±12256 | 0±0 | <0.001 |
|  | 619200±162295 | 43900±12738 | <0.001 | 774600±179051 | 54820±14589 | <0.001 |
|  | 19.64±2.97 | 0.46±1.44 | <0.001 | 18.59±2.66 | 0±0 | <0.001 |
|  | 21.91±9.18 | 0±0 | <0.001 | 23.29±8.05 | 0±0 | <0.001 |
|  | 160.24±15.87 | 82.8±21.3 | <0.001 | 164.46±8.34 | 80.42±16.76 | <0.001 |
|  | 104.38±10.5 | 55.12±11.38 | <0.001 | 111.38±9.25 | 54.62±12.32 | <0.001 |
|  | 18.67±2.13 | 75.32±10.22 | <0.001 | 19.57±2.52 | 76.6±8.14 | <0.001 |

**Plasma lipids, glucose, urea and homocysteine profile:** Plasma average concentrations (pg/ml ± SEM), n=10 for HDL, LDL, NEFA, triglycerides, glucose, urea and homocysteine before (0 minutes) and 30, 60, 90, 120, 180 and 300 minutes after the fava bean, lupin, meat, green pea, hemp and buckwheat meals are presented in Fig S3.

**HDL:** There was no significant diet effect on HDL (p=0.359) and LDL (p=0.280) when analyzed with ANOVA, there was no significant difference between meals for the AUC and iAUC values for HDL, p=0.865, and p=0.291 respectively (ANOVA).

**LDL:** There was no significant difference between meals for the AUC, and iAUC values for LDL, p=0.064, and p=0.298 respectively (ANOVA).

**NEFA:** Overall ANOVA analysis found significant diet and time effect on postprandial plasma NEFA (p<0.001, ANOVA) over 300 minutes, plasma concentrations being significantly higher after consumption of hemp and lupin meals than other test meals. ANOVA found significant effect of diet on AUC for NEFA (p<0.001, ANOVA), AUC for hemp and lupin meals being significantly higher than rest of the test meals; and no significant effect on iAUC (p=0.055). Significant effect on NEFA concentration at 90 minutes (p=0.005, ANOVA), NEFA plasma concertation after hemp meals was significantly higher than after meat and fava bean meals, and NEFA plasma concentration after lupin significantly higher than after green pea, fava bean and meat meals; NEFA plasma concentration after lupin and hemp meals significantly higher than after green pea, fava bean, buckwheat and meat meals at 120 minutes (p=0.004, ANOVA), at 150 minutes (p<0.001, ANOVA) and 180 minutes (p<0.001, ANOVA); at 300 minutes NEFA plasma concentration after lupin meal significantly higher than after green pea, fava bean, buckwheat and NEFA plasma concentration after hemp meal was significantly higher than buckwheat and green pea (p=0.018, ANOVA).

**Triglycerides:** Overall ANOVA found no significant diet effect on triglycerides (p=0.060), AUC (p=0.409) but a significant time effect (p<0.001) and a mild effect on iAUC (0.041), significantly higher after lupin meals iAUC (174.0) in comparison with fava bean, meat and buckwheat meals.

**Glucose:** Overall ANOVA found significant effect on diet and time on plasma glucose concentrations (p<0.001), these being significantly higher after consuming buckwheat meals. The ANOVA found a significant effect of test meals on AUC and iAUC for glucose (p=0.010, p=0.032 respectively), AUC after the buckwheat meal was significantly higher than all other meals, iAUC was significantly higher after buckwheat meal in comparison with green pea, meat and hemp meals. After consumption of buckwheat meals, plasma concentrations for glucose were significantly higher at 60 minutes (p=0.015, ANOVA), when compared with lupin, hemp, green pea, and meat meals; at 120 minutes (p=0.028, ANOVA), when compared with all the other meals; and at 150 minutes (p=0.001, ANOVA), when compared with hemp, fava bean, green pea and met meals.

**Urea:** Overall ANOVA found a significant diet (p<0.010) and time (p<0.001) effect on postprandial plasma urea over 300 minutes. ANOVA found no significant effect of test meals on AUC and significant effect for iAUC for urea (p=0.091, p=0.035 respectively), iAUC was significantly higher after meat and lupin meals in comparison with green pea, fava bean and buckwheat meals. After consumption of lupin meals. plasma urea concentrations were significantly higher at 150 and 180 minutes (p=0.047, p=0.015 respectively ANOVA), when compared with buckwheat, fava bean and green pea; and at 300 minutes (p<0.001, ANOVA), plasma concentration of urea were significantly higher after consumption of meat meals in comparison with buckwheat, fava bean, green pea, hemp meals.

**Homocysteine:** Overall ANOVA analysis found significant diet and time effect on postprandial plasma HCys (p<0.001) over 300 minutes, plasma HCys concentrations being significantly higher after fava bean meal in comparison with all the other test meals. ANOVA found no significant effect of test meals on AUC and significant effect for iAUC for urea (p=0.536, p<0.001 respectively), iAUC was significantly higher after fava bean meals in comparison with all the other meals. After consumption of fava bean meals plasma HCys concentration was significantly at 300 min (p=0.031, ANOVA) when compared with all the other test meals.


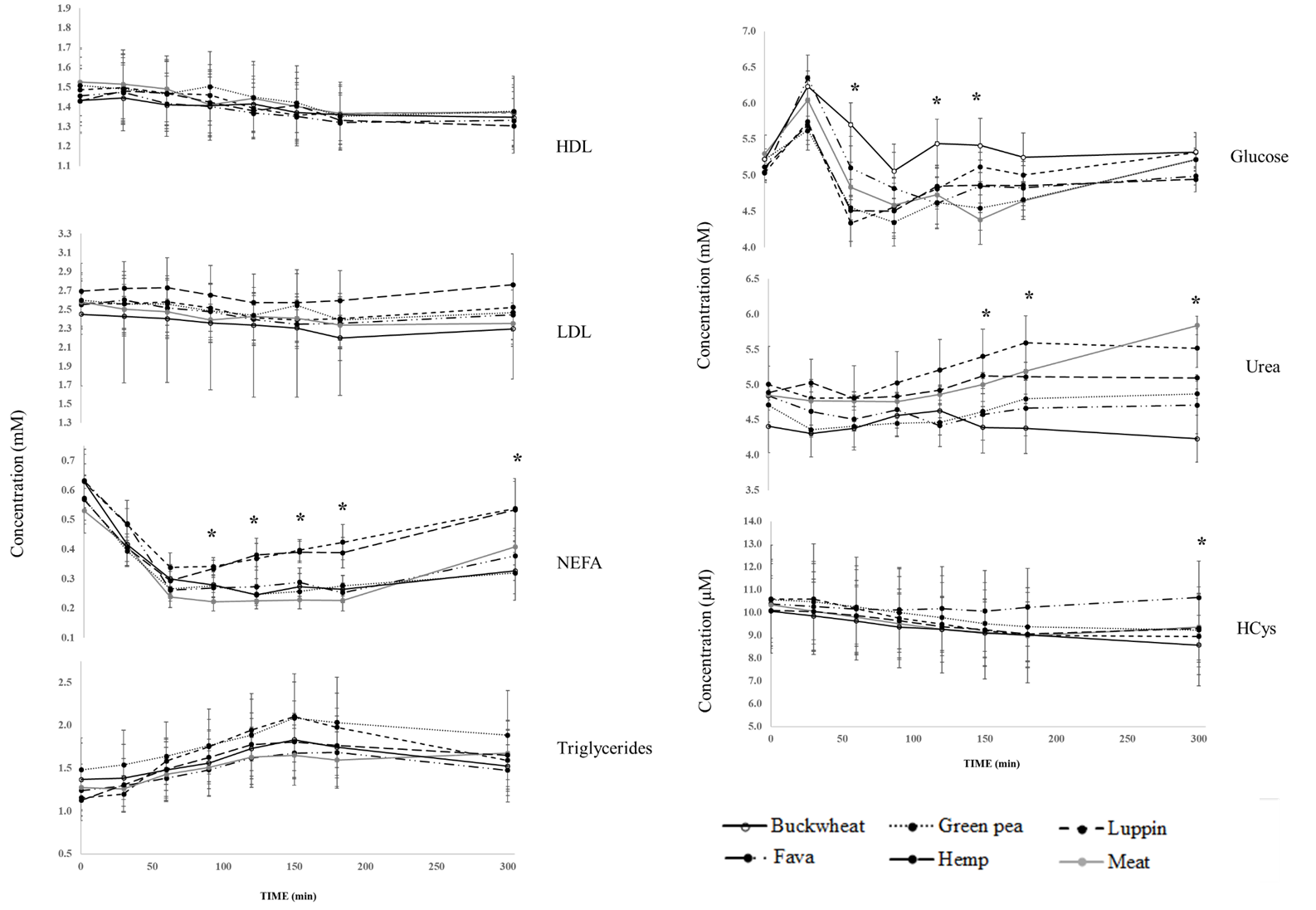


**Figure S 3.** Plasma average concentrations (pg/ml ± SEM), n=10 for HDL, LDL, NEFA, triglycerides, glucose, urea and homocysteine before (0 min) and 30, 60, 90, 120, 180 and 300 min after the fava bean, lupin, meat, green pea, hemp and buckwheat test meals. (*) NEFA represents significant effect on NEFA concentration at 90 min (p=0.005), at 120 min (p=0.004), 150 min (p<0.001), 180 min (p<0.001) and at 300 min (p=0.018). (*) Glucose represents ssignificant effect on glucose concentration at 60 min (p=0.015), at 120 min (p=0.028) and at 150 min (p=0.001). (*) Urea represents significant effect on urea concentration at 150 min (p=0.047), at 180 min (p=0.015), and at 300 min (p<0.001). (*) HCys represents significant differences in HCys concentrations at 300 min (p=0.031).

*****

*****
